# Lactones with Methylcyclohexane Systems Obtained by Chemical and Microbiological Methods and Their Antimicrobial Activity

**DOI:** 10.3390/molecules20023335

**Published:** 2015-02-16

**Authors:** Małgorzata Grabarczyk, Katarzyna Wińska, Wanda Mączka, Anna K. Żołnierczyk, Barbara Żarowska, Mirosław Anioł

**Affiliations:** 1 Department of Chemistry, University of Environmental and Life Sciences, Norwida 25, Wrocław 50-375, Poland; E-Mails: katarzyna.winska@up.wroc.pl (K.W.); wanda_m19@o2.pl (W.M.); anna.zolnierczyk@up.wroc.pl (A.K.Z.); miroslaw.aniol@up.wroc.pl (M.A.); 2 Department of Biotechnology and Food Microbiology, University of Environmental and Life Sciences, Chełmońskiego 37/41, Wrocław 51-630, Poland; E-Mail: barbara.zarowska@up.wroc.pl

**Keywords:** lactones, biotransformations, hydrolytic dehalogenation, *Fusarium* species, antimicrobial activity

## Abstract

Eight new lactones (δ-chloro-, δ-bromo- and δ-iodo-γ-lactones), each with a methylcyclohexane ring, were obtained by chemical means from (4-methylcyclohex-2-en-1-yl) acetic acid or (6-methylcyclohex-2-en-1-yl) acetic acid. Whole cells of ten fungal strains (*Fusarium* species, *Syncephalastrum racemosum* and *Botrytis cinerea*) were tested on their ability to convert these lactones into other products. Some of the tested fungal strains transformed chloro-, bromo- and iodolactone with a methyl group at C-5 into 2-hydroxy-5-methyl-9-oxabicyclo[4.3.0]nonan-8-one during hydrolytic dehalogenation. When the same lactones had the methyl group at C-3, no structural modifications of halolactones were observed. In most cases, the optical purity of the product was low or medium, with the highest rate for chlorolactone (45.4%) and iodolactone (45.2% and 47.6%). All of the obtained compounds were tested with reference to their smell. Seven halolactones and the hydroxylactone obtained via biotransformation of halolactones with 5-methylcyclohexane ring were examined for their antimicrobial activity. These compounds were capable of inhibiting growth of some bacteria, yeasts and fungi.

## 1. Introduction

Modern life sciences are focused on the search for new bioactive compounds of natural origin. It is reported that many plants used in traditional medicine systems contain a variety of compounds responsible for their healing properties. A large part of these compounds are sesquiterpenoid hydroxylactones [1,2,3,4,5,6], substances of high biological activity. Hydroxylactones exhibit a number of biological properties, showing e.g., hepaprotective [7], cytotoxic [8,9,10,11,12,13,14], antiplasmodial [15], antifungal [16], antibacterial [17], antitumor and anti-inflammatory activity [18], acting as phytotoxic agents [19], and having an inhibitory effect on NO production [20] and against activity of some enzymes [21].

However, the isolation of such compounds from plants is often complicated, because their content is usually not impressive. Therefore, it is necessary to look for alternative methods of the preparation of more potentially effective bioactive molecules. One such method is the use of microorganisms capable of introducing a hydroxy group into a molecule of, for example, a lactone. Halogenolactones or saturated lactones are useful compounds for this purpose [22,23,24,25,26,27,28,29].

As part of our ongoing research on the biotransformation of halolactones with a methyl-substituted cyclohexane rings [30,31,32,33,34,35], in this paper we present further examples of a biohydroxylation of chloro-, bromo- and iodolactones containing a methylcyclohexane ring. We examined the effect of the methyl group position in the cyclohexane ring on the ability of microorganisms to replace the halogen atom with a hydroxy group.

## 2. Results and Discussion

### 2.1. Synthesis of Substrates

**Scheme 1:**
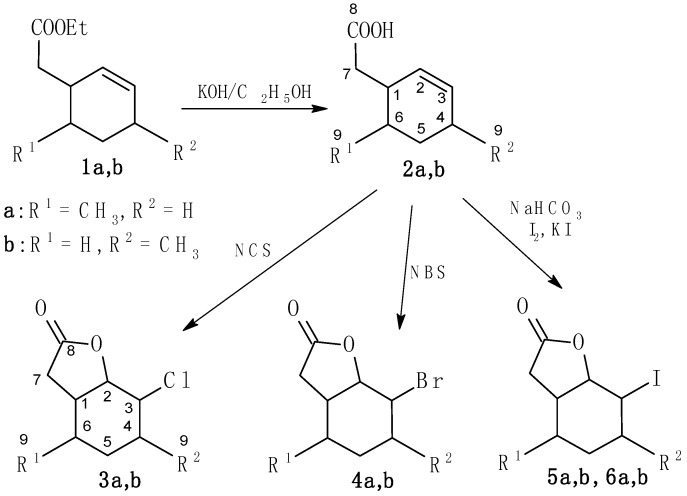
Synthesis of halolactones **3**–**6**.

In our research on compounds with a methylcyclohexane ring two intermediate products—γ,δ-unsaturated esters **1a** and **1b**—were obtained during earlier syntheses [36]. Following basic hydrolysis, these esters gave γ,δ-unsaturated acids **2a** [37] or **2b**, which were substrates for the next step. New chlorolactones **3a**,**b**, known bromolactone **4a** [38], and a new bromolactone **4b** were synthesized by chloro- or bromolactonization in THF [31]. Moreover, new iodolactones **5a**,**b** and **6a**,**b** were obtained by iodolactonization of acid **2**, according to the previously described procedure [39] (Scheme 1).

Chloro-**3a**,**b**, bromo-**4a**,**b** and iodolactones-**5a**,**b** were subjected to a screening biotransformation. Iodolactone **6a** was generated in small quantities, insufficient to carry out the biotransformation, so we decided to investigate only its biological activity. As for iodolactone **6b**, the amount obtained was only sufficient to determine its structure.

### 2.2. Biotransformation of Halolactones

One of our aims was to identify fungal strains capable of conducting a hydrolytic dehalogenation of these halolactones. The following strains were selected: *Fusarium culmorum* AM10, *Fusarium avenaceum* AM11, *Fusarium oxysporum* AM13*, Fusarium tricinctum* AM16, *Fusarium semitectum* AM20, *Fusarium equiseti* AM22, *Fusarium scirpi* AM1199*, Fusarium solani* AM203, *Syncephalastrum racemosum* AM105, and *Botrytis cinerea* AM235. Progress of all the screening transformations was monitored by using standard techniques (TLC and GC). The results of this step are displayed in Table 1.

**Table 1:** Hydrolytic dehalogenation of halolactones **3a**,**b**–**5a**,**b** after 7 days of incubation.

Entry	Microorganism	Chlorolactone		Bromolactone		Iodolactone	
		3a	3b	4a	4b	5a	5b
1	*Fusarium culmorum* AM10	(++)	(‒)	(++)	(‒)	(+++)	(‒)
2	*Fusarium avenaceum* AM11	(‒)	(‒)	(+++)	(‒)	(++++)	(‒)
3	*Fusarium oxysporum* AM13	(‒)	(‒)	(+)	(‒)	(+++)	(‒)
4	*Fusarium tricinctum* AM16	(‒)	(‒)	(‒)	(‒)	(‒)	(‒)
5	*Fusarium semitectum* AM20	(‒)	(‒)	(+)	(‒)	(+)	(‒)
*6*	*Fusarium equiseti* AM22	(+++)	(‒)	(++++)	(‒)	(++++)	(‒)
7	*Syncephalastrum racemosum* AM105	(‒)	(‒)	(‒)	(‒)	(+++)	(‒)
8	*Fusarium scirpi* AM199	(‒)	(‒)	(+++)	(‒)	(+++)	(‒)
9	*Fusarium solani* AM203	(++)	(‒)	(++)	(‒)	(+++)	(‒)
10	*Botrytis cinerea* AM235	(‒)	(‒)	(‒)	(‒)	(++)	(‒)

Conversions: (−): 0%–35%; (+): 45%–51%; (++): 53%–68%; (+++): 73%–89%; (++++): 90%–96%.

Halolactones **3a**–**5a** were transformed into a single product in all the biotransformations. The highest degree of conversion (over 90%) was observed for bromolactone **4a** (entry 6) and iodolactone **5a** (entry 2, 6). Some of the other microorganisms (entries 1, 8, 9 and 10 for **4a**, entries 1, 3, 7, 8, 9 and 10 for **5a**) transformed these two substrates with a good yield (over 53%). Chlorolactone **3a** was converted in good yield (over 53%) by only three microorganisms (entries 1, 6, 9). Iodolactone **6a** and **6b** were not subjected to the biotransformation, as they were produced in insufficient amounts for this step. The screening biotransformation was only used for a selection of fungal strains capable of converting the substrates into a product with a yield exceeding 50%. During the screening transformations, only the degree of conversion was evaluated.

The same ten fungal strains were investigated regarding their ability to transform halolactones **3b**–**5b** into other derivatives. Unfortunately, the tested strains were not capable of converting these substrates into any products.

Tests on the stability of lactones **3a**, **b**–**5a**, **b** were also conducted. The control was carried out by adding each of the lactones to a sterile medium containing 3 g of glucose and 1 g of peptobac in 100 mL of water and shaking for a week. Studied samples were prepared similarly as in the case of the screening procedure. We found that only the substrate was present in the reaction mixture, indicating that the hydrolytic dehalogenation of lactones **3a**, **b**–**5a**, **b** did not occur without the microbial participation.

**Figure 1:**
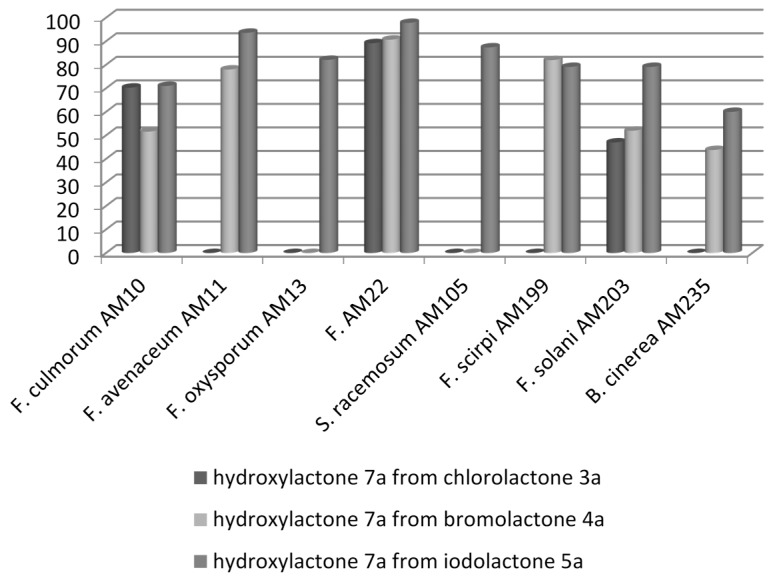
Results (by GC) of preparative biotransformations of lactones **3a**–**5a**.

For the next step, e.g., the preparative scale biotransformations, fungal strains that had been selected during the screening procedure were used. The results of these transformations are presented in Figure 1.

As expected, the best results were obtained when *F. equiseti* AM22 (degree of conversion 89.2% for **3a**, 90.7% for **4a**, and 97.8% for **5a**) was used as a biocatalyst. Very good results were also observed for *F. avenaceum* AM11 (degree of conversion 78.0% for **4a** and 93.6% for **5a**), *F. oxysporum* AM13 (degree of conversion 82.1% for **5a**) and *S. racemosum* AM105 (degree of conversion 87.4% for **5a**). From the microbial perspective, the best substrate for the biotransformation was iodolactone **5a**, converted into hydroxylactone **7a** by eight strains, and the worst was chlorolactone **3a**, transformed by only three strains. Data presented above pointed to the conclusion that the microorganisms used for the biotransformation of halolactones were characterized by substrate specificity.

**Scheme 2:**
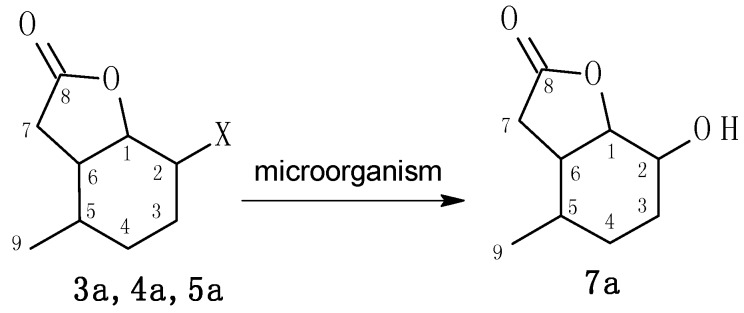
Biotransformations of halolactones **3a**–**5a**.

During all of the preparative biotransformations, a single product, hydroxylactone **7a**, was obtained (Scheme 2).

One of the aims of the described experiments was to obtain a product with high optical purity. Because of that we evaluated both the enantiomeric excess and optical rotation for every hydroxylactone obtained during the preparative biotransformations of halolactones **3a**–**5a**. The enantiomeric excess was determined using GC with a chiral column (Beta Dex, 30 m × 0.25 mm × 0.25 μm), and the optical rotation was measured in chloroform solutions. More details on the quantities of hydroxylactone **7a** and the data on its optical purity are presented in Table 2, Table 3 and Table 4.

**Table 2:** Data on hydroxylactone **7a** obtained during preparative biotransformations of lactone **3a**.

Entry	Strain	Yield (g)/(%)	ee	$\alpha_{D}^{20}$
1	*F.* *culmorum* AM10	0.017/18.3	45.4	‒33.344 (*c* = 0.69, CHCl_3_)
2	*F.* *equiseti* AM22	0.038/41.8	34.3	‒23.175 (*c* = 0.88, CHCl_3_)
3	*F. solani* AM203	0.012/13.3	17.9	+14.509 (*c* = 0.46, CHCl_3_)

**Table 3:** Data on hydroxylactone **7a** obtained during preparative biotransformations of lactone **4a**.

Entry	Strain	Yield (g)/(%)	ee	$\alpha_{D}^{20}$
1	*F.* *culmorum* AM10	0.024/32.4	32.4	‒22.477 (*c* = 0.85, CHCl_3_)
2	*F.* *avenaceum* AM11	0.032/10.4	10.4	+9.765 (*c* = 1.65, CHCl_3_)
3	*F.* *equiseti* AM22	0.024/18.0	32.5	‒17.042 (*c* = 0.61, CHCl_3_)
4	*F. scirpi* AM199	0.028/41.2	38.5	‒27.339 (*c* = 0.87, CHCl_3_)
5	*F. solani* AM203	0.034/5.6	46.6	+4.234 (*c* = 0.85, CHCl_3_)
6	*B. cinerea* AM235	0.021/20.2	28.9	‒19.213 (*c* = 0.94, CHCl_3_)

**Table 4:** Data on hydroxylactone **7a** obtained during preparative biotransformations of lactone **5a**.

Entry	Strain	yield (g)/(%)	ee	$\alpha_{D}^{20}$
1	*F.* *culmorum* AM10	0.016/26.7	45.2	‒32.244 (*c* = 0.89, CHCl_3_)
2	*F.* *avenaceum* AM11	0.018/29.7	5.7	‒6.598 (*c* = 0.80, CHCl_3_)
3	*F.* *oxysporum* AM13	0.035/57.2	35.3	+37.211 (*c* = 0.59, CHCl_3_)
4	*F.* *equiseti* AM22	0.036/58.5	15.1	‒17.740 (*c* = 0.97, CHCl_3_)
5	*S. racemosum* AM105	0.031/50.7	24.0	‒19.714 (*c* = 0.48, CHCl_3_)
6	*F. scirpi* AM199	0.030/49.6	47.6	‒34.134 (*c* = 0.65, CHCl_3_)
7	*F. solani* AM203	0.013/21.4	3.7	0 (*c* = 0.72, CHCl_3_)
8	*B. cinerea* AM235	0.014/23.2	45.2	‒32.196 (*c* = 0.49, CHCl_3_)

As a result of microbial dehydrodehalogenation, hydroxylactone **7a** was obtained with medium or low enantiomeric excess (ee). The best results were achieved using iodolactone **5a** as a substrate. Three microorganisms, *F. culmorum* AM10 (Table 4, entry 1), *F. scirpi* AM199 (Table 4, entry 6) and *B. cinerea* AM235 (Table 4, entry 8) were capable of transforming this compound with moderate enantiomeric excess (45.2% or 47.6%). Similar results were obtained for chlorolactone **3a** transformed by *F. culmorum* AM10 (ee = 45.4%) (Table 2, entry 1) and bromolactone converted by *F. solani* AM203 (ee = 46.6%) (Table 3, entry 5).

The majority of the microorganisms formed the (‒)-enantiomer of hydroxylactone **7a**. Interestingly, some of them, such as *F. avenaceum* AM11 for bromolactone **4a** (Table 3, entry 2), *F. oxysporum* AM13 for iodolactone **5a** (Table 4, entry 3) and *F. solani* AM203 for chlorolactone **3a** (Table 2, entry 3) and bromolactone **4a** (Table 3, entry 5) were able to form (+)-enantiomer.

### 2.3. Biological Tests

The synthetic halolactones **3a**, **b**–**5a**, **b** and **6a** and the microbiologically obtained hydroxylactone **7a** were subjected to biological tests. These tests were designed to identify the lactones capable of inhibiting or reducing growth of the selected microorganisms. Those microorganisms included an indicator bacterium *E. coli,* potentially pathogenic bacteria *S. aureus* and *B. subtilis*, a pathogenic bacterium *C. albicans* and *Fusarium linii* and *Aspergillus niger* that are capable of producing mycotoxins. The effects of the halolactones **3a**–**6a** on the growth of bacteria, yeast and fungi are presented in Table 5 and Table 6.

**Table 5:** The effects of the halolactones **3a**–**6a** on the growth of bacteria, yeast and fungi.

Entry	Strain	OD Control	max.OD of 3a	max.OD of 4a	max.OD of 5a	max.OD of 6a	max.OD of 7a
1	*E. coli*	1.56	1.53 ^a^	1.57 ^a^	1.46 ^a^	1.50 ^a^	1.50
2	*S. aureus*	1.55	1.20	1.56	1.58	1.22	1.45
3	*B. subtilis*	1.37	1.39 ^b^	1.38 ^b^	1.40 ^b^	1.40	1.56
4	*C. albicans*	1.96	1.56 ^c^	0.41	0.95	1.80 ^c^	1.90
5	*S. cerevisiae*	2.30	0.80	0.70	1.37	1.67	0.37
6	*Y. lipolytica*	1.40	0.38	0.49	0.89	1.05 ^c^	1.41
7	*F. linii*	1.96	0.32	0.36	0.45	1.51	2.45
8	*A. niger*	3.0	2.00 ^d^	0.81	1.22	1.16	2.71

Notes: ^a^ rapid elimination of bacterial cells (reduced OD after 15–20 h of the culture); ^b^ longer lag phase (up to 3 h) and biphasic growth; ^c^ great elongation of the lag-phase, up to 25–28 h; ^d^ longer lag-phase, up to 10 h.

**Table 6:** The effects of the halolactones **3b**–**5b** on the growth of bacteria, yeast and fungi.

Entry	Strain	OD Control	Max.OD of 3b	Max.OD of 4b	Max.OD of 5b
1	*E. coli*	1.56	1.56	0.72	1.46
2	*S. aureus*	1.55	1.00	0.84	1.30
3	*B. subtilis*	1.4	1.00 ^a^	1.20 ^a^	1.20
4	*C. albicans*	1.96	1.82	1.85	0.58
5	*S. cerevisiae*	2.30	0.70	0.70	1.11
6	*Y. lipolytica*	1.39	0.39	0.36	1.00 ^b^
7	*F. linii*	2.05	0.35	0.37	0.31
8	*A. niger*	3.0	0.48	0.36	2.41 ^c^

Notes: ^a^ biphasic growth; ^b^ great elongation of the lag-phase; ^c^ longer lag phase up to 10 h.

The results, presented in Table 5, indicate that some of the tested lactones showed complete growth inhibition mainly against *Y. lipolytica* (Entry 6) and *F. linii* (Entry 7) strains. Rapid elimination of bacterial cells was observed for the *E. coli* strain (Entry 1). Some of the lactones were also capable of extending the lag-phase of microorganisms—*B. subtilis* (Entry 3) and *C. albicans* (Entry 4). In the case of compounds **3a**–**5a** biphasic growth for *B. subtilis* (Entry 3) was observed.

Data presented in Table 6 indicates that some of the tested lactones showed complete growth inhibition, mainly against *Y. lipolytica* (Entry 6) and *A. niger* (Entry 8) strains. Iodolactone **5a** was able to elongate the lag-phase of *Y. lipolytica* (Entry 6), *F. linii* (Entry 7) and *A. niger* (Entry 8). Similarly as in the case of lactones **3a**–**5a** (Table 5) biphasic growth for *B. subtilis* (Entry 3) was also observed.

### 2.4. Discussion

The performed syntheses yielded eight racemic halo-γ-halolactones **3a**, **b**–**6a**, **b**, consisting of a methylcyclohexane ring connected to a lactone ring. Their structures were determined based on their spectral data (^1^H-NMR, ^13^C-NMR, COSY, HMQC and IR), confirmed by GC-MS and elemental analysis.

#### 2.4.1. Characterization of Compounds **3a**–**6a** with 5-Methylcyclohexane Rings 

The spectroscopic data indicated that acid **2a** was a diastereoisomeric *cis-trans* (82%:18%) mixture. The coupling constant between the H-6 proton and the CH_3_-9 methyl group protons was equal to 6.6 Hz, which suggested an equatorial orientation of this methyl group. Wide multiplets coming from the H-6 proton and H-1 proton indicated their axial orientation. When this acid was subjected to chloro- or bromolactonization the creation of only one lactone (**3a** or **4a**) was observed, while the iodolactone was created as a mixture of compounds **5a** and **5b**. Such result can be explained by different mechanisms of chloro-, bromo- and iodolactonization. According to Snider [40] and Jayaraman [41] during chloro- and bromolactonization the double bond is attacked by bromine leading to the formation of a bromonium ion. In the process of iodolactonization the complex of iodine and double bond is attacked by the carboxylate anion.

**Figure 2:**
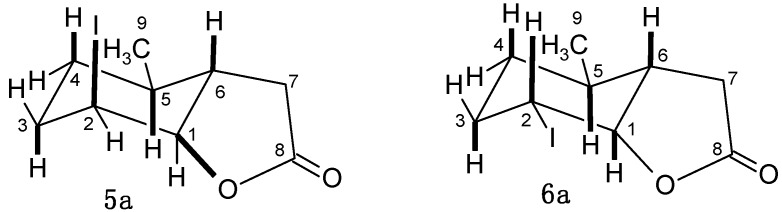
Structures of iodolactones **5a** and **6a**.

The absorption bands at 1778, 1784, 1784 and 1784 cm^−1^ in the IR spectra for these lactones indicated that the lactone ring present in their molecules was a γ-lactone. As claimed in the earlier works [31,33,34], in similar compounds the cyclohexane ring exists in the chair conformation. The analysis of the ^1^H-NMR spectra of lactones **3a**, **4a** and **5a** indicated that in these lactones the cyclohexane ring also adopted the chair conformation. Signals from the H-1 proton (4.62, 4.63, 4.76 ppm) and H-2 proton (4.61, 4.61, 4.69 ppm) looking like singlets suggested that these protons were located in a *trans*-diequatorial positions. The wide multiplet of the H-6 proton suggested its axial position. This also implied that the halogen atom and C-O bond were in *trans*-diaxial positions. In the case of iodolactone **6a**, the cyclohexane ring was also in a chair conformation, but the coupling constants of the H-1 proton (dd, *J* = 9.9 and 7.3 Hz) and H-2 proton (ddd, *J =* 13.7, 9.9 and 4.4 Hz) suggested their *trans*-diaxial positions. It means that in this case, the halogen atom and C-O bond were in *trans* diequatorial positions. Doublet of doublet of doublet (*J =* 13.7, 7.4 and 7.4 Hz), coming from H-6 proton, also indicated its axial position. The wide multiplet corresponding to the H-5 proton suggested its axial orientation, which indicated that the methyl group CH_3_-9 was in an equatorial position in the moleculesof halolactones **3a**–**6a** (Figure 2).

**Figure 3:**
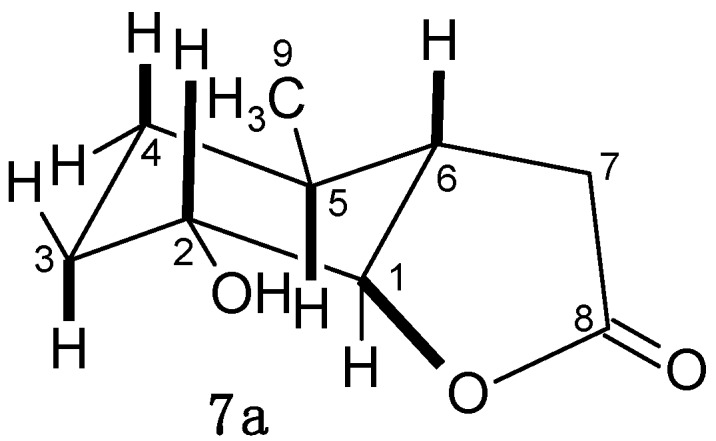
Structure of hydroxylactones **7a**.

Only one product was obtained during all the preparative scale biotransformations performed on lactones **3a**, **4a** and **5a**. Their structures were established based on the spectral data. The IR spectra showed that the γ-lactone ring (absorption band at 1769 cm^−1^) was retained in the product during the biotransformation. The presence of a hydroxy group in the molecule was suggested by the strong and broad band found at 3305 cm^−1^. Analysis of NMR spectra (^1^H, ^13^C, COSY and HMQC) of halolactones **3a**–**5a** and the microbiologically obtained hydroxylactone **7a** was helpful in determining the differences between these molecules. Major difference was observed for the H-1 and H-2 protons. The signal of the H-1 proton changed from a singlet (in the substrates) into a triplet (*J =* 3.8 Hz), while the signal of the H-2 proton became doublet of doublets of doublets with coupling constants *J =* 11.6, 4.7 and 3.8 Hz. This means that the H-1 proton retained its equatorial position (as in the substrates), but the H-2 proton changed its position from equatorial in the substrates into axial in the products (Figure 3).

**Scheme 3:**
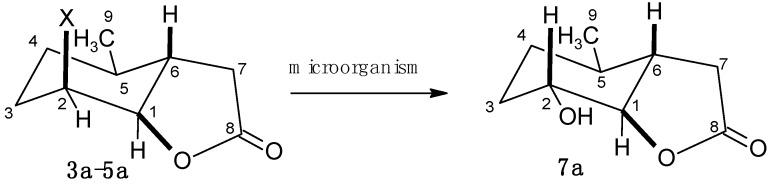
Equatorial location of the hydroxy group in lactone **7a** as the stereochemical consequence of microbial hydrolytic dehalogenation (similar to a S_N_2 mechanism) of chloro-**3a**, bromo-**4a** and iodolactone **5a** proceeding with inversion at C-2.

The data presented above suggests that the halogen atom present in the substrate molecule was replaced by a hydroxy group during a hydrolytic dehalogenation similar to a S_N_2 mechanism. Such a mechanism was observed earlier for similar halolactones subjected to hydrolytic dehalogenation by the same microorganisms [31,33,34], and also for enzymes from *Absidia cylindrospora* culture that catalyze hydrolytic dehalogenations in iodolactones [30]. We assume that the hydroxylactone **7a** was formed as a result of the hydrolytic dehalogenation which mechanism is similar to the one described for haloalkane dehalogenase [42]. According to this mechanism one of the oxygens of the aspartate carboxylate group of the enzyme attacked the carbon C-2 to which the halogen was bound and replaced it by a S_N_2 substitution mechanism. During the next step the covalent alkyl-enzyme intermediate, to which the halide was still bound, was hydrolyzed by a molecule of water. This hydrolysis afforded the final product with an equatorial hydroxy group at C-2 (Scheme 3).

Knowing from the earlier experiments [33,34] that the microbiologically obtained hydroxylactones would be characterized by significant enantiomeric purity, the enantiomeric excess and optical rotation for hydroxylactones obtained during all preparative biotransformation were checked. As shown in Table 3, the enantiomeric excess was usually low or moderate. The best results (enantiomeric excess over 45%) were obtained for hydroxylactone **7a** obtained from chlorolactone **3a**, when *F. culmorum* AM10 was used as a biocatalyst and iodolactone **5a** was transformed by *F. culmorum* AM10, *S. racemosum* AM105 and *B. cinerea* AM235. Most of the tested microorganisms preferably formed the (‒)-isomer of the hydroxylactone. Only *F. avenaceum* AM11 for lactone **4a**, *F. oxysporum* AM13 for lactone **5a** and *F. solani* AM203 for all three lactones used as substrates formed the (+)-isomer of hydroxylactone **7a**.

#### 2.4.2. Characterization of Compounds with 3-Methylcyclohexane Ring (**3b**–**6b**)

**Figure 4:**
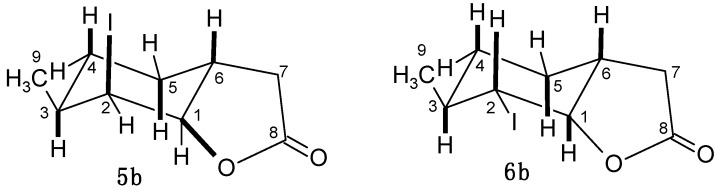
Structure of iodolactones **5b** and **6b**.

Acid **2b** was a diastereoisomeric 79:21 *cis-trans* mixture similar to acid **2a**. In this case we also observed formation of only one pure compound during chloro- or bromolactonization-**3b** and **4b**, and the mixture of two iodolactones **5b** and **6b**. This situation also can be explained by the different mechanisms of chloro-, bromo- and iodolactonization described above for compounds **3a**–**5a**. Analysis of the ^1^H-NMR spectra of lactones **3b**–**5b** revealed similarity between these lactones and the previous ones **3a**–**5a**. For these lactones, the cyclohexane ring also adopts the chair conformation. The signals of the H-1 proton (4.58, 4.71, 4.84 ppm), which appears as a multiplet with a small coupling constant, and the H-2 proton (4.39, 4.53, 4.73 ppm) being doublet of doublets with small coupling constants (3.3 and 3.2 Hz, 3.6 and 3.2 Hz, 3.6 and 3.0 Hz), indicated that these protons were located in the *trans-*diequatorial position. This also suggested that the halogen atom and C-O bond were in *trans*-diaxial positions. The structure of iodolactone **6b** was similar to the structure of its **6a** analog. In this case, the cyclohexane ring was in the chair conformation with H-1 (dd, *J =* 9.0 and 7.0 Hz) and H-2 (dd, *J =* 10.8 and 9.0 Hz) protons, located in the *trans*-diaxial position. The wide multiplet from the H-6 proton suggested its axial position (Figure 4).

As presented in Table 1, none of the tested microorganisms were capable of introducing a hydroxy group in the place of the halogen atom. This was probably due to the presence of a methyl group located at the C-3 carbon. It is well known that the mechanism of the hydrolytic dehalogenation is similar to a S_N_2 one, therefore, the presence of a methyl group at C-3 probably prevents activation of this mechanism.

#### 2.4.3. The Assessment of the Effects of Lactones on the Growth of Tested Microorganisms

Analysis of the results obtained during the biological tests leads to the conclusion that filamentous fungi and yeast were the most susceptible species, and bacteria the most resistant, to the tested lactones. Chlorolactones **3a**, **3b**, bromolactone **4a** and iodolactones **5a**, **5b** were able to completely inhibit the growth of some yeast and fungi, and the other halolactones also exhibited a limiting effect. Surprisingly hydroxylactone **7a** was the least active against the tested strains. It showed activity only against the *S. aureus* and *S. cerevisiae* strains. In the presence of lactone **7a** the population of *S. aureus* was similar to that of the control cells, but after some time the bacterial cells began to die off. In the second case, the complete inhibition of *S. cerevisiae* growth was observed. During the tests, biphasic growth of *B. subtilis* was noticed in the presence of most of the lactones. This could mean that these bacteria were capable of using the lactones as a growth-inducing factor. It is supported by a known ability of *B. subtilis* to degrade *N*-acyl-l-homoserine lactone [43].

**Figure 5:**
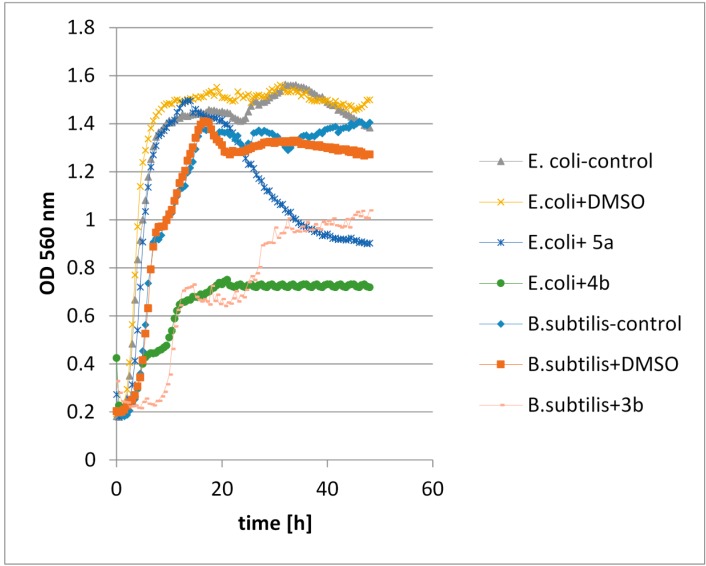
Examples of the effects of selected lactones on the chosen bacteria strains.

**Figure 6:**
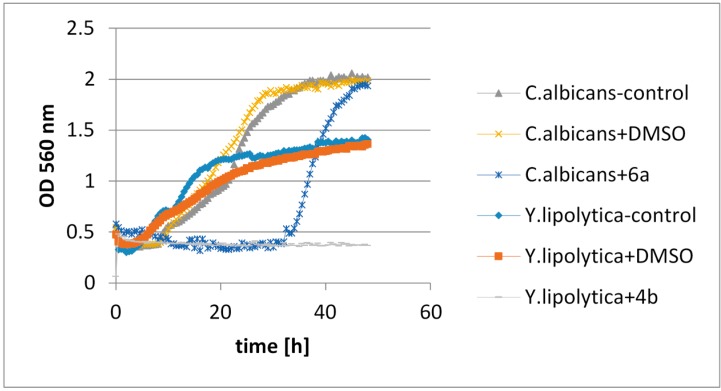
Examples of the effects of selected lactones on the chosen yeast strains.

The greatest inhibitory effect on bacterial growth was shown by halolactones with a methyl group located at the C-3 carbon (compounds **3b**, **4b**, **5b**). The same compounds showed a weak effect on yeast growth. In this case better results were obtained for halolactones with a methyl group located at the C-5 carbon (**3a**, **4a**, **5a**). The tests on fungal strains demonstrated that the position of the methyl group did not matter. In general, the compounds which were able to inhibit the growth of microorganisms to the greatest extent were the chlorolactones. It was also interesting that lactones with a halogen atom in an axial position showed stronger inhibitory effects against microorganisms than these with a halogen located in an equatorial position. Examples of the effects of selected lactones on the chosen microorganisms are presented in Figure 5 and Figure 6.

#### 2.4.4. An olfactory Analysis of Lactones

Knowing from previous experiments [36] that some derivatives obtained during the synthesis of hydroxylactones with the methylcyclohexane ring are characterized by specific smells, we decided to check the odor of the compounds described in this paper. It was found that an overwhelming majority of them had slight, uninteresting or unpleasant (decayed leftovers in the case of bromolactone **4b**) odors. Only three compounds gave off an interesting smell:

- acid **2a**—medium intensive, sweet-sour, dry pine scent;
- chlorolactone **3b**—slight dried fruit scent;
- iodolactone **5b**—slight scent of dust, reminiscent of an old pharmacy.

## 3. Experimental Section

### 3.1. General

Progress of all chemical reactions, biotransformations and purity of isolated products were checked by TLC on silica gel-coated aluminium plates (DC-Alufolien Kieselgel 60 F254, Merck, Dramstadt, Germany) and also by GC analysis performed on a CP-3380 instrument (Varian, Agilent Technologies, Santa Clara, CA, USA) using an DB-17 column (cross-linked methyl silicone gum, 30 m × 0.32 mm × 0.25 µm). Temperatures during GC analysis were as follows: injector 150 °C, detector (FID) 280 °C, column temperature: 100 °C (hold 1 min), 100–200 °C (rate 10 °C/min), 200–280 (rate 50 °C/min), 300 °C (hold 1 min). The structures of obtained compounds were also confirmed by gas chromatography-mass spectrometry (GC-MS) analysis using Varian SATURN 2000 instrument (*EI* ionization) with HP-1 column (cross-linked methyl silicone gum, 25 m × 0.32 mm × 0.52 µm) under the following conditions: injector 200 °C, detector 300 °C, column temperature: 120 °C (hold 2 min), 120–300 °C (rate 20 °C/min), 300 °C (hold 3 min). The enantiomeric excess of the products obtained during biotransformation were determined by GC analysis using chiral column CP-cyclodextrin-B-325 (30 m × 0.25 mm × 0.25 µm) under the following conditions: injector 200 °C, detector (FID) 220 °C, column temperature: 140 °C (hold 45 min), 140–200 °C (rate 20 °C/min), 200 °C (hold 1 min). All products were purified by using of preparative column chromatography on silica gel (Kieselgel 60, 230–400 mesh). Melting points were determined on a Boetius apparatus. Refractive index was measured on Carl Zeiss (Jena, Germany) Abbe and Pulfrich refractometers. Elemental analysis was done using a Vario EL III CHNS automatic analyzer from Elemental Analyzersysteme (Wigan, UK). ^1^H-NMR, ^13^C-NMR, ^1^H-^13^C COSY (HMQC) and ^1^H-^1^H COSY spectra were recorded in a CDCl_3_ solution on an Avance DRX 300 spectrometer (Bruker, Billerica, MA, USA). IR spectra were determined using FTIR on a Thermo-Mattson IR 300 spectrometer (Waltham, MA, USA). Optical rotations were measured on P-2000 polarimeter (Jasco, Easton, PA, USA).

### 3.2. Synthesis of Substrates

#### 3.2.1. (6–Methylcyclohex-2-en-1-yl)acetic Acid (**2a**)

Basic hydrolysis of ester **1a** (9.2 g, 0.046 mol), according to the known procedure [31] yielded 6.0 g (77%) of acid **2a** as a 82:18 diastereoisomeric *cis-trans* mixture with the following physical and spectral properties: n_D_ = 1.4694, ^1^H-NMR (CDCl_3_): 0.89 (d, *J =* 6.9 Hz, 3H, CH_3_-9 *trans*), 1.00 (d, *J =* 6.6 Hz, 3H, CH_3_-9 *cis*), 1.31–1.50 (m, 4H, CH_2_-5), 1.56–1.62 (m, 1H, H-6 *trans*), 1.64–1.72 (m, 1H, H-6 *cis*), 1.98–2.05 (m, 4H, CH_2_-4), 2.18–2.23 (m, 4H, one of CH_2_-7 and H-1), 2.53–2.62 (dd, *J =* 18.9 and 8.9 Hz, 2H, one of CH_2_-7), 5.53–5.59 (m, 2H, H-3), 5.67–5.74 (m, 2H, H-2), ^13^C-NMR (CDCl_3_): 19.85 (C-9), 24.19 (C-4), 29.27 (C-5), 32.96 (C-6), 38.95 (C-1), 39.20 (C-7), 127.71 (C-3), 129.15 (C-2), 178.43 (C-8), IR (KBr, cm^−1^): 2921 (s), 1709 (s), 1411 (s), 1284 (s). EI-MS *m/z* (%) = 154 (8) [M]^+^, 108 (11), 94 (100), 79 (81), 67 (46), 43 (34), 39 (45). EA for C_9_H_14_O_2_ (154.21) calculated 70.10% C, 9.15% H, found 70.19% C, 9.10% H.

#### 3.2.2. 2-Chloro-5-methyl-9-oxabicyclo[4.3.0]nonan-8-one (**3a**)

Chlorolactonization of a diastereoisomeric 82:18 *cis-trans* mixture of acid **2a** (2.5 g, 0.016 mol), according to the procedure of Grabarczyk and Białońska [31] gave 1.6 g (76%) of chlorolactone **3a** with the following physical and spectral properties: n_D_ = 1.4993, ^1^H-NMR (CDCl_3_): 1.01 (d, *J =* 6.5 Hz, 3H, CH_3_-9), 1.30 (m, 1H, H-5), 1.56 (m, 2H, CH_2_-4), 1.85 (m, 1H, one of CH_2_-3), 1.99 (m, 1H, CH_2_-3), 2.47 (m, 1H, H-6), 2.43 (d, *J =* 17.0 Hz, 1H, one of CH_2_-7), 2.67 (dd, *J =* 17.0 and 6.7 Hz, 1H, one of CH_2_-7), 4.53 (m, 2H, H-1 and H-2), ^13^C-NMR (CDCl_3_): 19.84 (C-9), 25.43 (C-4), 27.92 (C-3), 31.72 (C-5), 36.30 (C-7), 39.19 (C-6), 54.91 (C-2), 80.96 (C-1), 175.91 (C-8), IR (KBr, cm^−1^): 2952 (s), 1778 (s), 1454 (s), 1147 (s), 972 (s). EI-MS *m/z* (%): 153 (66) [M−HCl], 126 (17), 112 (68), 84 (68), 69 (100), 56 (18), 43 (50), 39 (33). EA for C_9_H_13_ClO_2_ (188.66), 57.30% C, 6.95% H, found 57.20% C, 6.90% H.

#### 3.2.3. 2-Bromo-5-methyl-9-oxabicyclo[4.3.0]nonan-8-one (**4a**)

After bromolactonization acid **2a** (1.7 g, 0.011 mol of an 82:18 diastereoisomeric *cis-trans* mixture), according to the known method [31], 1.6 g (61%) of bromolactone **3** was obtained. The physical and spectral data of this product are as follows: m.p. = 47–48 °C, ^1^H-NMR (CDCl_3_): 1.03 (d, *J =* 6.5 Hz, 3H, CH_3_-9), 1.32 (m, 1H, H-5), 1.61 (m, 2H, CH_2_-4), 2.06 (m, 2H, CH_2_-3), 2.32 (m, 1H, H-6), 2.45 (d, *J =* 17.0 Hz,1H, one of CH_2_-7), 2.66 (dd, *J =* 17.0 and 6.6 Hz,1H, one of CH_2_-7), 4.64 (s, 1H, H-2), 4.65 (s, 1H, H-1), ^13^C-NMR (CDCl_3_): 19.85 (C-9), 26.48 (C-4), 28.33 (C-3), 31.82 (C-5), 36.95 (C-7), 39.19 (C-6), 47.64 (C-2), 81.30 (C-1), 176.00 (C-8), IR (KBr, cm^−1^): 2957 (s), 1784 (s), 1427 (s), 1153 (s), 966 (s). EI-MS *m/z* (%): 153 (11) [M−HBr], 111 (39), 95 (22), 85 (100), 67 (33), 55 (42), 41 (26), 39 (46). EA for C_9_H_13_BrO_2_ (233.11) calculated 46.37% C, 5.62% H, found 46.29% C, 5.66% H.

#### 3.2.4. 2-Iodo-5-methyl-9-oxabicyclo[4.3.0]nonan-8-one (**5a**) and 2-Iodo-5-methyl-9-oxabicyclo[4.3.0]-nonan-8-one (**6a**)

Iodolactonization of 1.8 g (0.012 mol) of acid **2a** (82:18 diastereoisomeric *cis-trans* mixture), according to the known procedure [39] gave iodolactone **5a** (1.9 g, 58%) and iodolactone **6a** (0.3 g, 9.4%) with the following physical and spectral properties: **5a**: m.p.=67–68 °C, ^1^H-NMR (CDCl_3_): 1.04 (d, *J =* 6.5 Hz, 3H, CH_3_-9), 1.31 (m, 1H, H-5), 1.61 (m, 2H, CH_2_-4), 1.85 (m, 1H, one of CH_2_-3), 2.00 (dd, *J =* 15.3 and 3.1 Hz, 1H, one of CH_2_-3), 2.40 (m, 1H, H-6), 2.45 (d, *J =* 16.9 Hz, 1H, one of CH_2_-7), 2.65 (dd, *J =* 16.9 and 6.6 Hz, 1H, one of CH_2_-7), 4.74 (s, 1H, H-1), 4.82 (m, 1H, H-2), ^13^C-NMR (CDCl_3_): 19.88 (C-9), 27.66 (C-2), 28.56 (C-4), 29.43 (C-3), 32.03 (C-5), 37.08 (C-7), 39.26 (C-6), 83.09 (C-1), 176.27 (C-8), IR (KBr, cm^−1^): 2924 (s), 1786 (s), 1457 (s), 1148 (s), 960 (s). EI-MS *m/z* (%): 153 (100) [M-HI], 112 (26), 84 (25), 69 (37), 43 (21), 39 (13). EA for C_9_H_13_IO_2_ (280.11) calculated 38.59% C, 4.68% H, found 38.48% C, 4.57% H. **6a**: m.p. = 48–49 °C, ^1^H-NMR (CDCl_3_): 0.98 (d, *J =* 7.2 Hz, 3H, CH_3_-9), 1.22 (m, 1H, one of CH_2_-4), 1.47 (m, 1H, one of CH_2_-4), 1.95 (m, 2H, one of CH_2_-3 and H-5), 2.25 (dd, *J =* 17.2 and 8.2 Hz, 1H, one of CH_2_-7), 2.33 (dd, *J =* 17.2 and 13.7 Hz, 1H, one of CH_2_-7), 2.38 (m, 1H, one of CH_2_-3), 2.70 (ddd, *J =* 13.7, 7.4 and 7.4 Hz, 1H, H-6), 3.80 (ddd, *J =* 13.7, 9.9 and 4.4 Hz, 1H, H-2), 4.70 (dd, *J =* 9.9 and 7.3 Hz, 1H, H-1), ^13^C-NMR (CDCl_3_): 19.49 (C-9), 27.17 (C-2), 27.40 (C-7), 30.48 (C-4), 31.01 (C-5), 36.59 (C-3), 42.80 (C-6), 86.24 (C-1), 175.13 (C-8), IR (KBr, cm^−1^): 2936 (s), 1784 (s), 1454 (s), 1177 (s), 1016 (s). EI-MS *m/z* (%): 153 (100) [M−HI], 135 (26), 127 (13), 107 (20), 93 (28), 81 (36), 67 (21), 55 (34), 39 (31). EA for C_9_H_13_IO_2_ (280.11) calculated 38.59% C, 4.68% H, found 38.51% C, 4.60% H.

#### 3.2.5. (4–Methylcyclohex-2-en-1-yl)acetic Acid (**2b**)

After basic hydrolysis of ester **1b** (1.5 g, 0.0075 mol), according to the known procedure [31], acid **2b** (1.1 g, 95%) was obtained as a diastereoisomeric 79:21 *cis-trans* mixture. The physical and spectral data of this product are as follows: n_D_ = 1.4654, ^1^H-NMR (CDCl_3_): 0.96 (d, *J =* 6.9 Hz, 3H, CH_3_-9 *cis*), 0.97 (d, *J =* 6.9 Hz, 3H, CH_3_-9 *trans*), 1.16-1.23 (m, 4H, CH_2_-6), 1.69–1.72 (m, 2H, CH_2_-5 *cis*), 1.81–1.93 (m, 2H, CH_2_-5 *trans*), 2.12–2.17 (m, 2H, H-4), 2.29–2.37 (m, 4H, CH_2_-7), 2.50–2.60 (m, 2H, H-1), 5.48–5.62 (m, 4H, H-2 and H-3), ^13^C-NMR (CDCl_3_): 21.31 and 21.64 (C-9), 27.88 and 28.89 (C-6), 29.69 and 30.44 (C-4), 30.86 and 31.50 (C-5), 32.51 and 33.04 (C-1), 40.19 and 40.67 (C-7), 128.82 and 128.97 (C-3), 134.65 and 134.72 (C-2), 178.64 (C-8), IR (KBr, cm^−1^): 2928 (sb), 1709 (s), 1411 (s), 1292 (s). EI-MS *m/z* (%): 154 (8) [M]^+^, 136 (5), 108 (9), 94 (100), 79 (61), 67 (39), 53 (8), 39 (31). EA for C_9_H_14_O_2_ (154.21) calculated 70.10% C, 9.15% H. Found: 70.04% C, 9.10% H.

#### 3.2.6. 2-Chloro-3-methyl-9-oxabicyclo[4.3.0]nonan-8-one (**3b**)

Chlorolactonization of a 79:21 diastereoisomeric *cis-trans* mixture of acid **2b** (0.5 g, 0.0032 mol) according to the known procedure [31] yielded chlorolactone **3b** (0.32 g, 53%) with the following physical and spectral properties: n_D_ = 1.4914, ^1^H-NMR (CDCl_3_): 1.09 (d, *J =* 6.7 Hz, 3H, CH_3_-9), 1.24 (dd, *J =* 11.7 and 3.6 Hz, 1H, one of CH_2_-4), 1.39 (dd, *J =* 13.7 and 3.4 Hz, 1H, one of CH_2_-5), 1.56 (m, 1H, one of CH_2_-5), 1.85 (m, 1H, one of CH_2_-4), 2.05 (m, 1H, H-5), 2.26 (d, *J =* 15.9 Hz,1H, one of CH_2_-7), 2.66–2.71 (m, 2H, H-6 and one of CH_2_-7), 4.43 (m, 1H, H-2), 4.61 (dd, *J =* 3.3 and 3.2 Hz, 1H, H-1), ^13^C-NMR (CDCl_3_): 18.50 (C-9), 24.72 (C-5), 27.24 (C-4), 30.80 (C-3), 30.99 (C-6), 37.92 (C-7), 61.81 (C-2), 81.47 (C-1), 176.00 (C-8), IR (KBr, cm^−1^): 2936 (s), 1783 (s), 1455 (s), 1144 (s), 981 (s). EI-MS *m/z* (%): 189 (27) [M]^+^, 167 (12), 159 (22), 153 (68), 109 (45), 93 (89), 81 (43), 67 (62), 55 (53), 39 (100). EA for C_9_H_13_ClO_2_ (188.66), 57.30% C, 6.95% H, found 57.24% C, 6.88% H.

#### 3.2.7. 2-Bromo-3-methyl-9-oxabicyclo[4.3.0]nonan-8-one (**4b**)

Using the previously described method [31] 0.28 g (62%) of bromolactone **4b** was obtained from 0.3 g (0.0019 mol) of 79:21 diastereoisomeric *cis-trans* mixture of acid **2b**. The physical and spectral data of the product are as follows: n_D_ = 1.5181, ^1^H-NMR (CDCl_3_): 1.03 (d, *J =* 6.6 Hz, 3H, CH_3_-9), 1.21 (m, 1H, one of CH_2_-4), 1.37 (m, 1H, one of CH_2_-5), 1.52 (m, 1H, one of CH_2_-5), 1.80 (m, 2H, H-3 and one of CH_2_-4), 2.23 (d, *J =* 16.1 Hz,1H, one of CH_2_-7), 2.65 (dd, *J =* 16.1 and 6.4 Hz,1H, one of CH_2_-7), 2.75 (m, 1H, H-6), 4.53 (m, 1H, H-2), 4.71 (dd, *J =* 3.6 and 3.2 Hz, 1H, H-1), ^13^C-NMR (CDCl_3_): 20.26 (C-9), 25.83 (C-5), 27.37 (C-4), 30.66 (C-3), 31.00 (C-6), 38.23 (C-7), 57.08 (C-2), 81.88 (C-1), 176.13 (C-8), IR (KBr, cm^−1^): 2930 (s), 1788 (s), 1423 (s), 1138 (s), 950 (s). EI-MS *m/z* (%): 153 (100) [M−HBr], 135 (23), 109 (43), 93 (37), 81 (16), 67 (39), 55 (17), 39 (35). EA for C_9_H_13_BrO_2_ (233.11) calculated 46.37% C, 5.62% H, found 46.42% C, 5.70% H.

#### 3.2.8. 2-Iodo-3-methyl-9-oxabicyclo[4.3.0]nonan-8-one (**5b**) and 2-Iodo-3-methyl-9-oxabicyclo-[4.3.0]nonan-8-one (**6b**)

Iodolactonization of acid **2b** (0.3 g, 0.0019 mol of 79:21 diastereoisomeric *cis-trans* mixture), according to a known procedure [39] gave iodolactones **5b** (0.29 g, 53%) and **6b** (0.05 g, 9%) with the following physical and spectral properties: **5a**: n_D_ = 1.5525, ^1^H-NMR (CDCl_3_): 0.96 (d, *J =* 5.0 Hz, 3H, CH_3_-9), 1.22 (m, 2H, CH_2_-4), 1.37 (m, 1H, H-3), 1.83 (ddd, *J =* 10.1, 6.4 and 3.3 Hz, 1H, H-5), 2.27 (d, *J =* 16.7 Hz, 1H, one of CH_2_-7), 2.66 (dd, *J =* 16.7 and 6.4 Hz, 1H, one of CH_2_-7), 2.82 (m, 1H, H-6), 4.73 (m, 1H, H-2), 4.84 (dd, *J =* 3.6 and 3.0 Hz, 1H, H-1), ^13^C-NMR (CDCl_3_): 23.54 (C-9), 27.61 (C-5), 28.10 (C-3), 30.41 (C-4), 31.10 (C-6), 38.78 (C-7), 41.82 (C-2), 83.83 (C-1), 176.44 (C-8), IR (KBr, cm^−1^): 2925 (s), 1784 (s), 1454 (s), 1146 (s), 974 (s). EI-MS *m/z* (%): 153 (100) [M−HI], 135 (27), 107 (28), 93 (50), 79 (10), 67 (9), 55 (11), 39 (25). EA for C_9_H_13_IO_2_ (280.11) calculated 38.59% C, 4.68% H, found 38.71% C, 4.61% H. **5b**: oil, ^1^H-NMR (CDCl_3_): 1.12 (d, *J =* 6.5 Hz, 3H, CH_3_-9), 1.72–1.78 (m, 5H, H-3, CH_2_-4, CH_2_-5), 2.31 (dd, *J =* 17.2 and 8.4 Hz, 1H, one of CH_2_-7), 2.45 (dd, *J =* 17.2 and 11.8 Hz, 1H, one of CH_2_-7), 2.67 (m, 1H, H-6), 3.70 (dd, *J =* 10.8 and 9.0 Hz, 1H, H-2), 4.78 (dd, *J =* 9.0 and 7.0 Hz, 1H, H-1), ^13^C-NMR (CDCl_3_): 23.77 (C-9), 27.78 (C-5), 27.90 (C-4), 31.59 (C-7), 35.58 (C-6), 38.02 (C-3), 39.44 (C-2), 86.25 (C-1), 175.32 (C-8), IR (KBr, cm^−1^): 2954 (s), 1772 (s), 1464 (s), 1178 (s), 952 (s). EA for C_9_H_13_IO_2_ (280.11) calculated 38.59% C, 4.68% H, found 38.53% C, 4.63% H.

### 3.3. Biotransformations

#### 3.3.1. Microorganisms

The fungal strains used in all biotransformations came from the collection of the Institute of Biology and Botany, Medical University, Wrocław, Poland (*Fusarium culmorum* AM10, *Fusarium avenaceum* AM11, *Fusarium oxysporum* AM13*, Fusarium tricinctum* AM16, *Fusarium semitectum* AM20, *Fusarium equiseti* AM22, *Fusarium scirpi* AM1199*, Fusarium solani* AM203, *Syncephalastrum racemosum* AM105 and *Botrytis cinerea* AM235). These strains were cultivated on Sabouraud’s agar containing 0.5% of aminobac, 0.5% of peptone, 4% of glucose and 1.5% of agar dissolved in distilled water at 28 °C and stored in refrigerator at 4 °C. Screening and preparative biotransformations were performed as described before [34]. The physical and spectral data of *2-hydroxy-5-methyl-9-oxabicyclo[4.3.0]nonan-8-one* (**7a**) are as follows: m.p. = 89–90 °C, ^1^H-NMR (CDCl_3_): 0.93 (d, *J =* 6.4 Hz, 3H, CH_3_-9), 1.06 (m, 1H, one of CH_2_-4), 1.26 (m, 1H, H-5), 1.53 (m, 1H, one of CH_2_-3), 1.73 (m, 1H, one of CH_2_-4), 1.88 (m, 1H, one of CH_2_-3), 2.00 (m, 1H, H-6), 2.10 (m, 1H, OH), 2.40 (d, *J =* 16.9 Hz, 1H, one of CH_2_-7), 2.68 (dd, *J =* 16.9 and 6.6 Hz, 1H, one of CH_2_-7), 3.72 (ddd, *J =* 11.6, 4.7 and 3.8 Hz, 1H, H-2), 4.57 (t, *J =* 3.8 Hz, 1H, H-1), ^13^C-NMR (CDCl_3_): 19.60 (C-9), 28.86 (C-3), 30.78 (C-4), 31.88 (C-5), 37.02 (C-7), 42.98 (C-6), 70.08 (C-1), 81.83 (C-2), 176.33 (C-8), IR (KBr, cm^−1^): 3305 (sb), 2927 (s), 1769 (s), 1160 (s). EI-MS *m/z* (%):170 (6) [M]^+^, 170, 153 (11), 123 (8), 111 (39), 85 (100), 67 (33), 39 (42). EA for C_9_H_14_O_3_ (170.21) calculated 63.51% C, 8.29% H, found 63.43% C, 8.18% H

#### 3.3.2. Bioassays

The tests were conducted on certain bacteria strains: *Escherichia coli*, *Staphylococcus aureus*, *Bacillus subtilis*, yeast strains*: Candida albicans*, *Saccharomyces cerevisiae*, *Yarrowia lipolytica* and also filamentous fungi strains: *Aspergillus niger* and *Fusarium linii*. The bacterial cultures were grown for 48 h in a liquid broth consisting of 15 g of dry bullion (Biocorp, Warszawa, Poland) and 10 g of glucose dissolved in 1 L of distilled water. Yeast and fungi cultures were growing in YPG medium which was composed of 10 g of yeast extract, 10 g of bacteriological peptone and 10 g of glucose dissolved in 1 L of distilled water for 48 and 96 h, respectively. The test were performed in the automated Bioscreen C system (Automated Growth Curve Analysis System, Lab systems, Helsinki, Finland).

Working volume in the wells of the Bioscreen plate was 300 µL, comprised of 280 µL of culture medium, 10 µL of cells or spore solution (final concentration). Tested lactones were dissolved in DMSO and used at the final concentration of 0.1% (w/v). Temperature was controlled at 30 °C (bacteria, yeasts) and 25 °C (filamentous fungi). The optical density of the cell suspensions was measured automatically at 560 nm in regular intervals of 30 min, for 3–4 days. The cell cultures were continuously shaken. Each culture was performed in three replicates. Data was analyzed by using spreadsheet software (Excel 97) and were calculated as the averages from the tree replicate analysis for each type of culture medium. The averages were used to generate the growth curves for each strain studied, constructed as a function of the incubation time and the absorbancy of the culture medium. The resulting microbial growth curves were compared to control cultures in medium supplemented with DMSO.

#### 3.3.3. Odour Evaluation

The odour evaluation was performed for ethanolic solutions (10%) of samples with the use of a strip blotter.

## 4. Conclusions

Two-step chemical synthesis yielded eight halolactones with methylcyclohexane rings. Six of them were subjected to a screening biotransformation by ten fungal strains. Most of these strains were capable of transforming the halolactones with the methyl group at the C-4 position into the corresponding hydroxylactone. The selected microorganisms showed very high regioselectivity, because the hydroxy group was introduced at C-2 in the equatorial position due to the operative S_N_2 mechanism. The tested strains were also characterized by substrate specificity—iodolactone **5a** was transformed at the greatest rate (eight strains) and chlorolactone **3a** at the lowest rate (only three strains). The best results were obtained when *F. equiseti* AM22, *F. avenaceum* AM11, *F. oxysporum* AM13 and *S. racemosum* AM105 were used as biocatalysts. Degrees of conversion of the substrates **3a**–**5a** were over 80%. Most of the microorganisms formed the (‒)-isomer of the hydroxylactone and the (+)-isomer was produced only by two microrganisms. The halolactones **3a**–**6a** and **3b**–**5b** and hydroxylactone **7a** were tested for their ability to inhibit the growth of some bacteria, yeast and fungal strains. The biological tests indicated the ability of these compounds to inhibit or limit growth of most microorganisms. Odor evaluation of all the described compounds revealed that most of them were characterized by uninteresting smells, except for acid **2a**, chlorolactone **3b** and iodolactone **5b**. The smell of acid **2a** (sweet sour, dry pine) was particularly interesting. Chlorolactone **3b** and iodolactone **5b** were characterized by a slight dried fruit scent and a slight dusty scent, respectively.

## Supplementary Materials

Supplementary materials can be accessed at: http://www.mdpi.com/1420-3049/20/02/3335/s1.
