# Supplementary material for: Lactones with Methylcyclohexane Systems Obtained by Chemical and Microbiological Methods and Their Antimicrobial Activity
Source: Molecules. 2015 Feb 16;20(2):3335–53. doi: 10.3390/molecules20023335 (PMC6272377; doi:10.3390/molecules20023335)
Supplement: Supplementary file 1 [file molecules-20-03335-s001.pdf]

Supplementary Materials

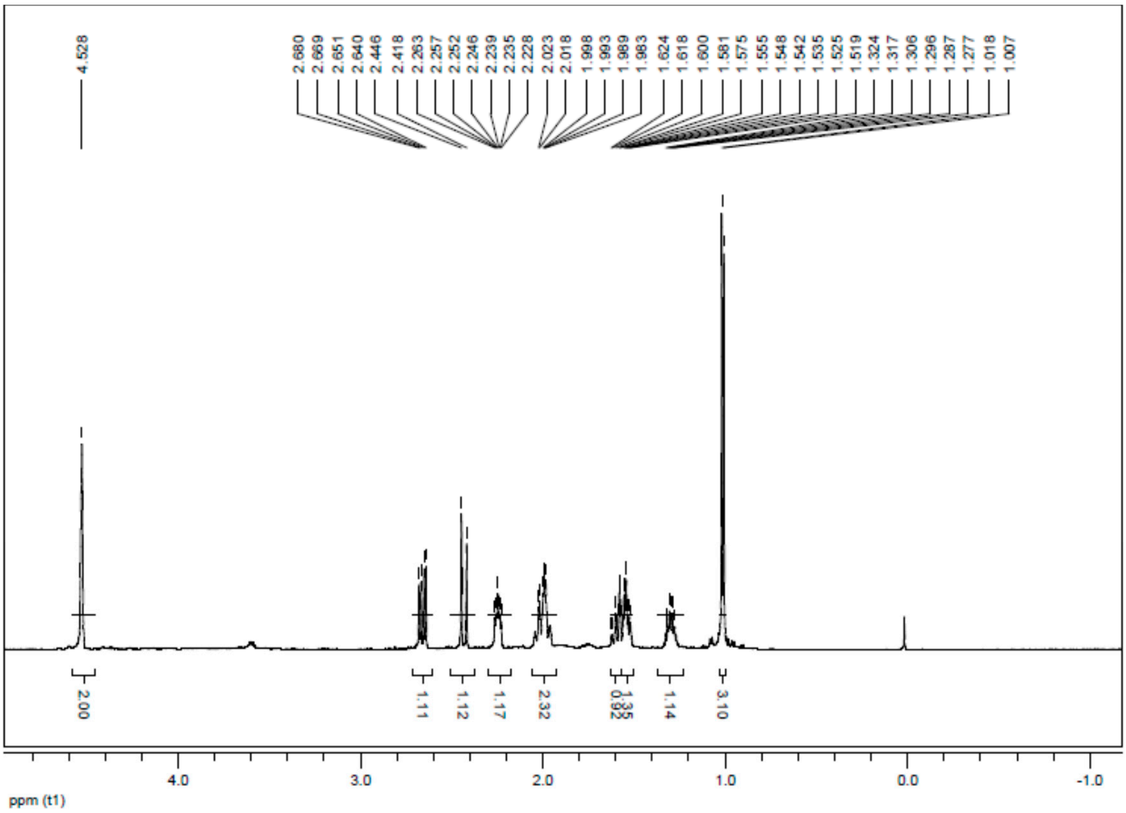

Figure S1. ^{1}H-NMR (300 MHz, $CDCl_3$) spectrum of chlorolactone **3a**.

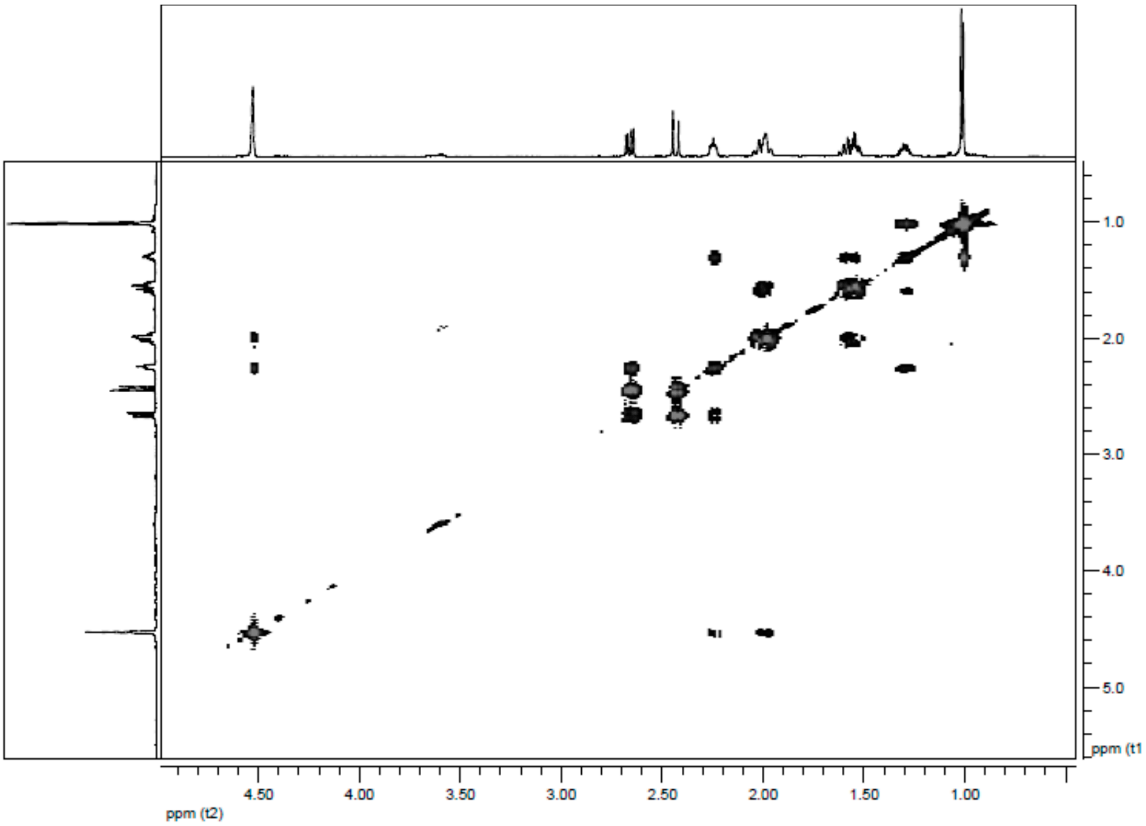

Figure S2. COSY (300 MHz, $CDCl_3$) spectrum of chlorolactone **3a**.

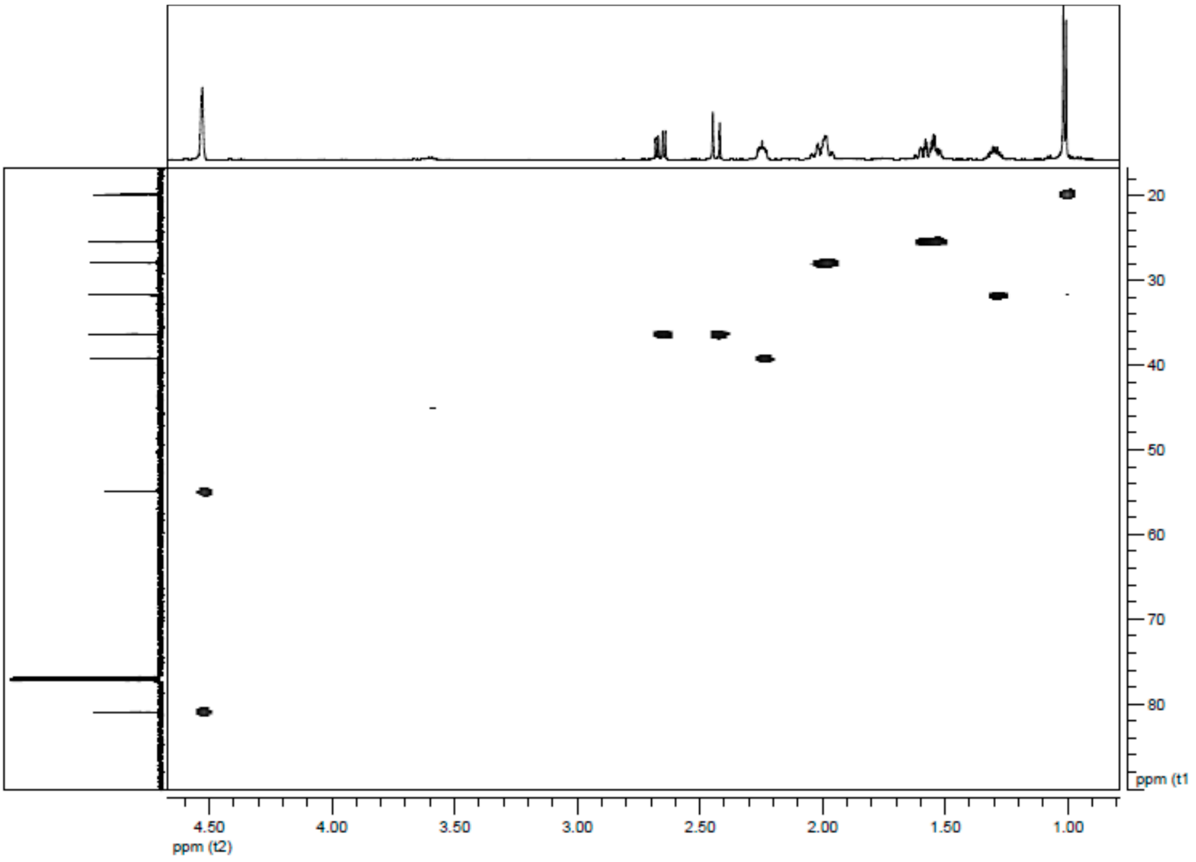

Figure S3. HMQC (300 MHz, $CDCl_3$) spectrum of chlorolactone **3a**.

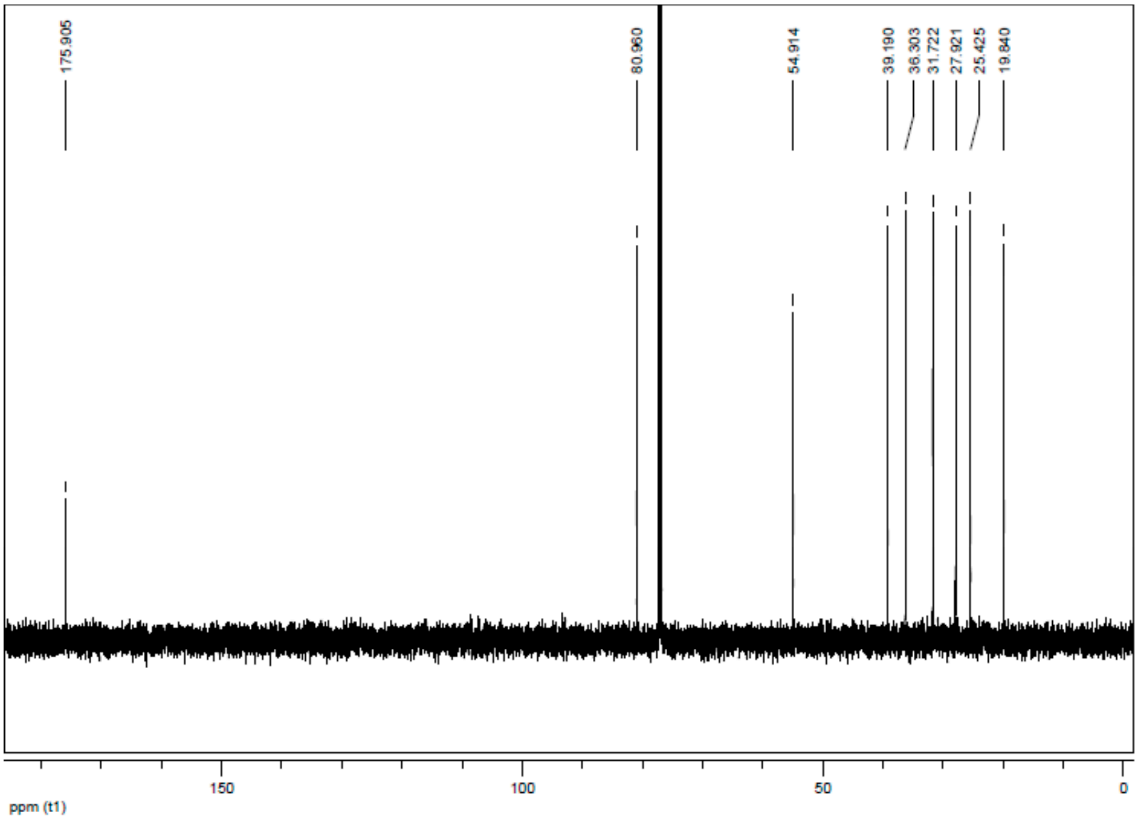

Figure S4. ^{13}C-NMR (75.5 MHz, $CDCl_3$) spectrum of chlorolactone **3a**.

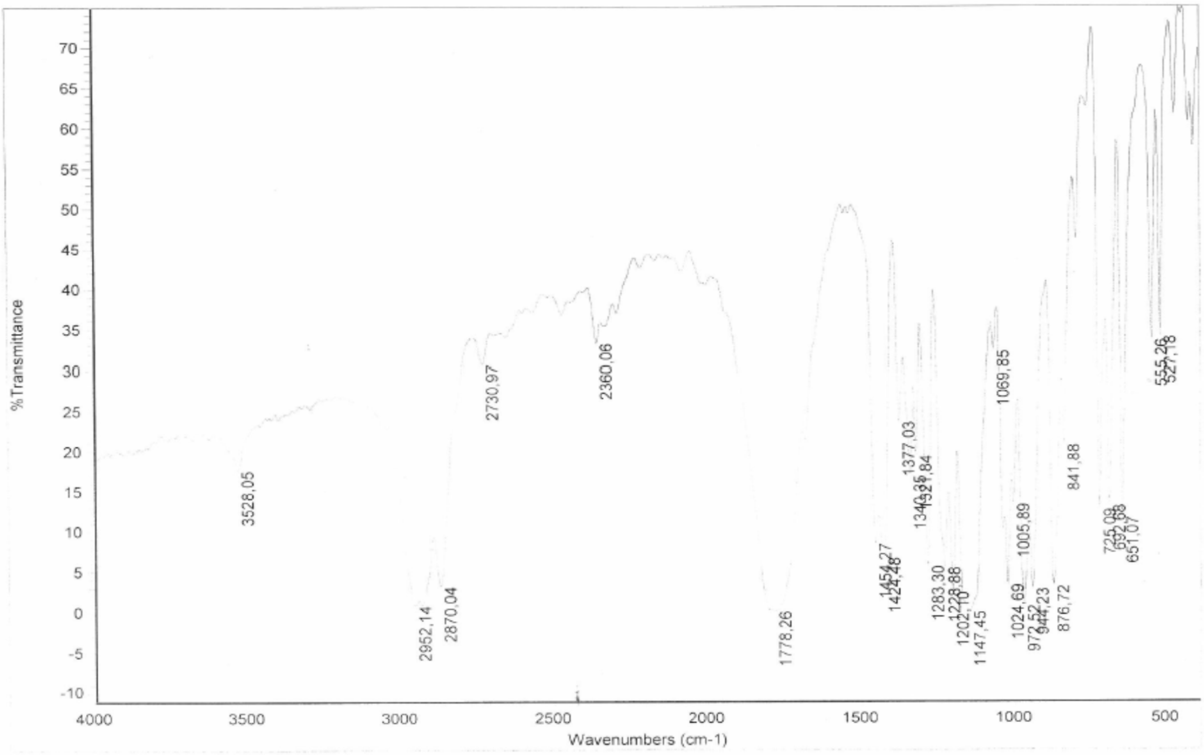

Figure S5. IR spectrum of chlorolactone **3a**.

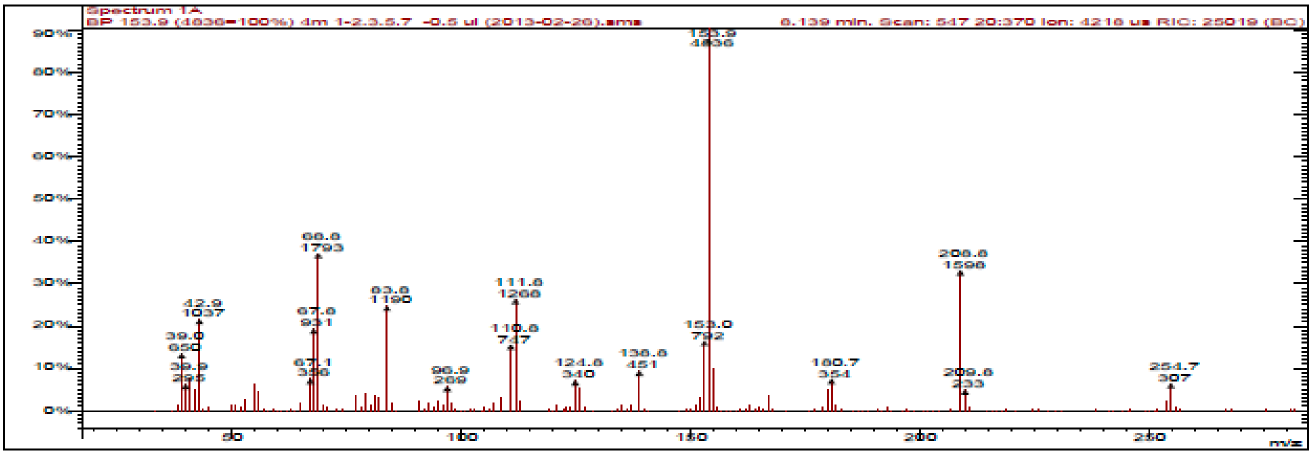

Figure S6. GC-MS spectrum of chlorolactone **3a**.

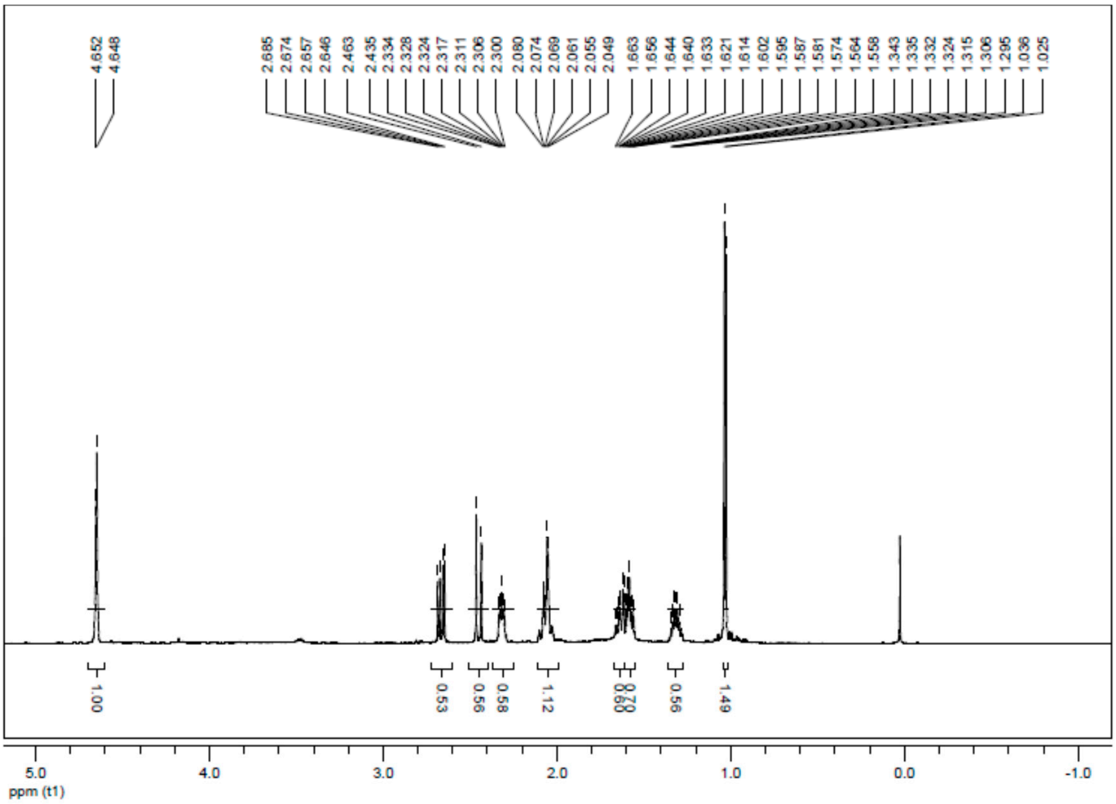

Figure S7. ^{1}H-NMR (300 MHz, $CDCl_3$) spectrum of bromolactone **4a**.

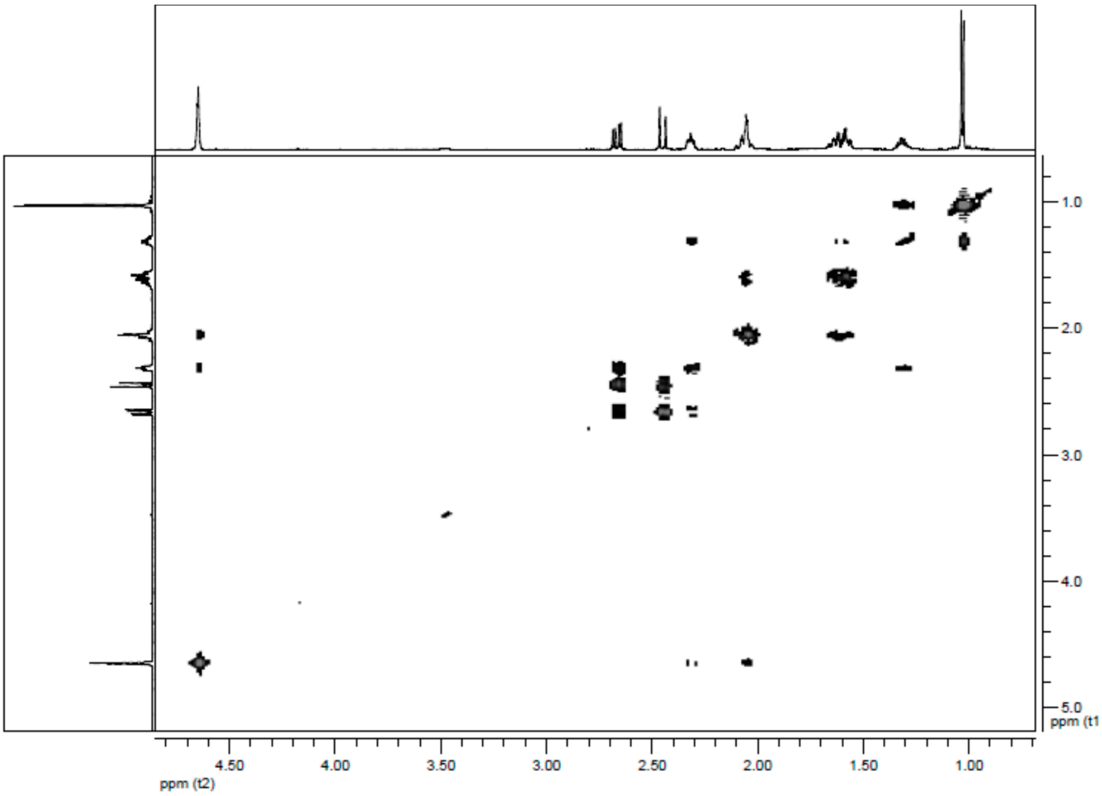

Figure S8. COSY (300 MHz, $CDCl_3$) spectrum of bromolactone **4a**.

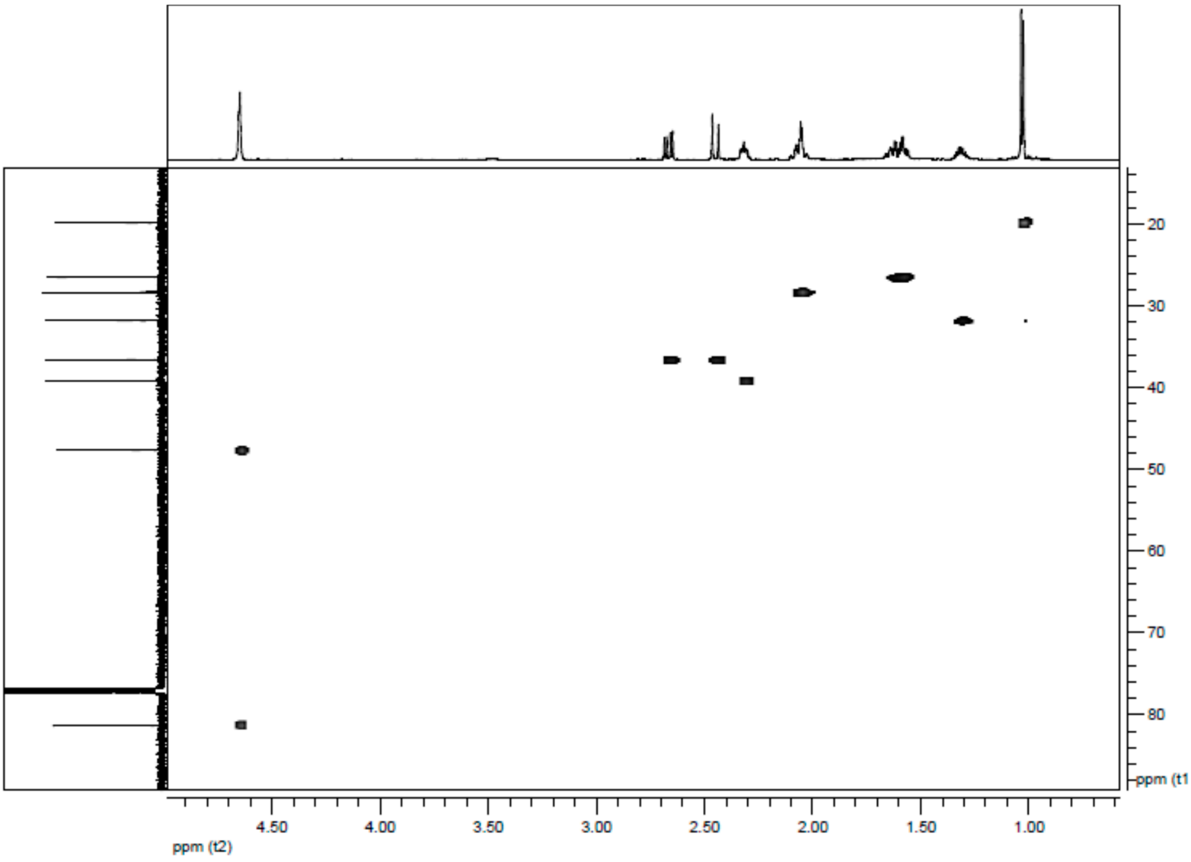

Figure S9. HMQC (300 MHz, $CDCl_3$) spectrum of bromolactone **4a**.

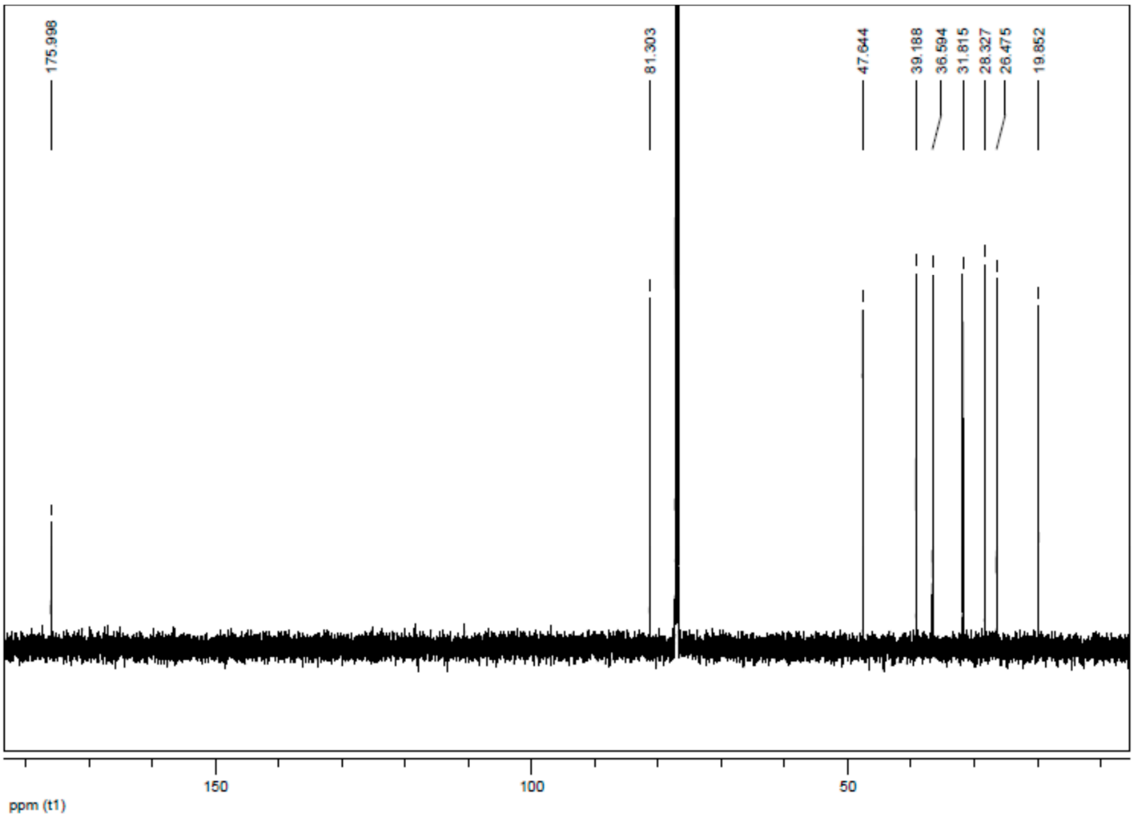

Figure S10. ^{13}C-NMR (75.5 MHz, $CDCl_3$) spectrum of bromolactone **4a**.

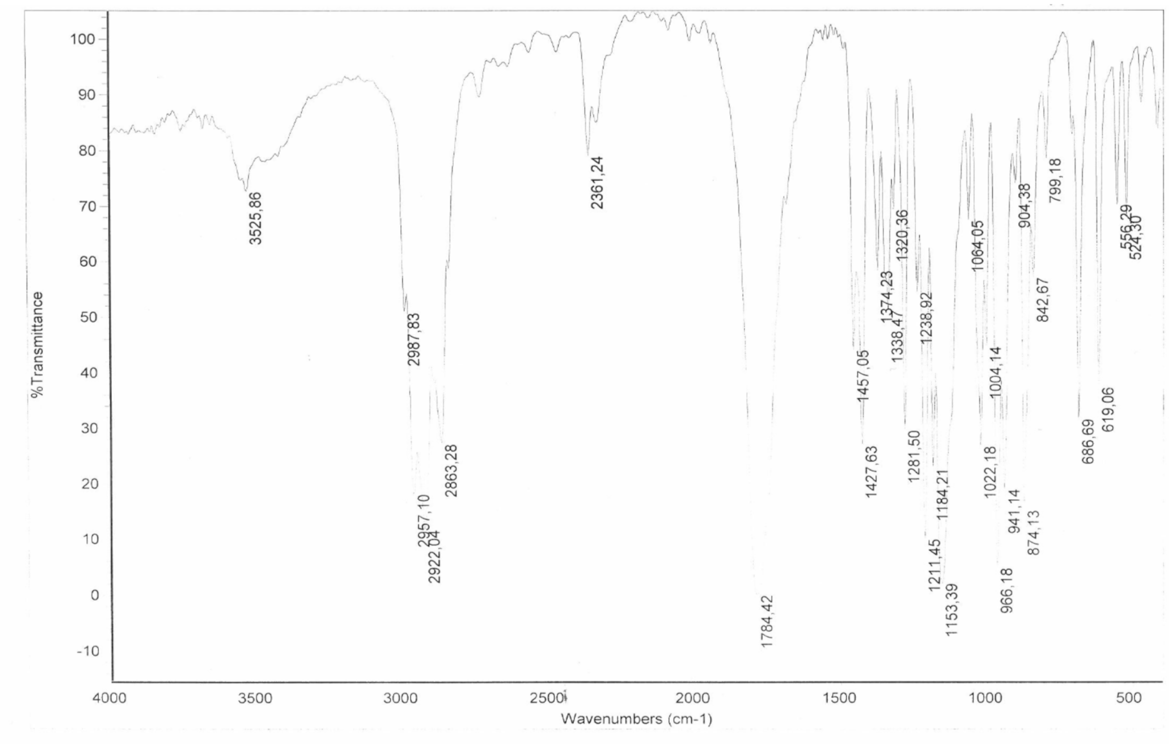

Figure S11. IR Spectrum of bromolactone **4a**.

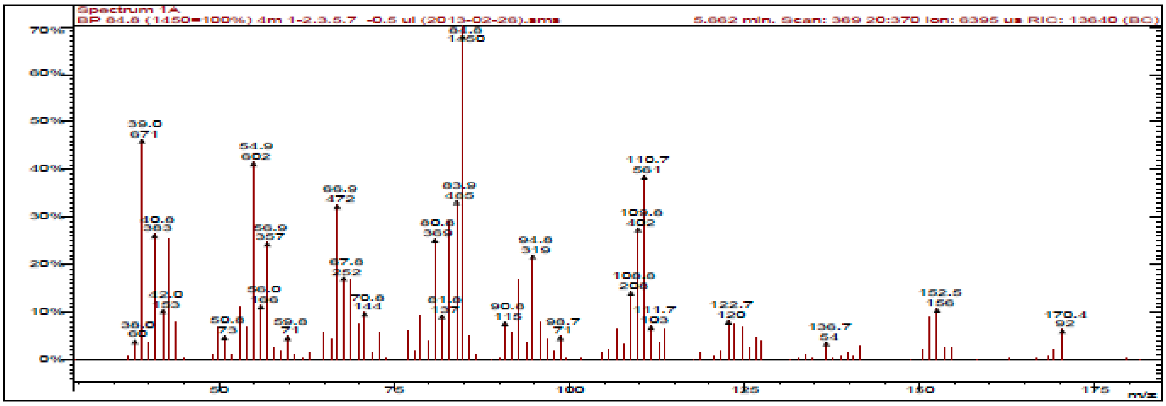

Figure S12. GC-MS spectrum of bromolactone **4a**.

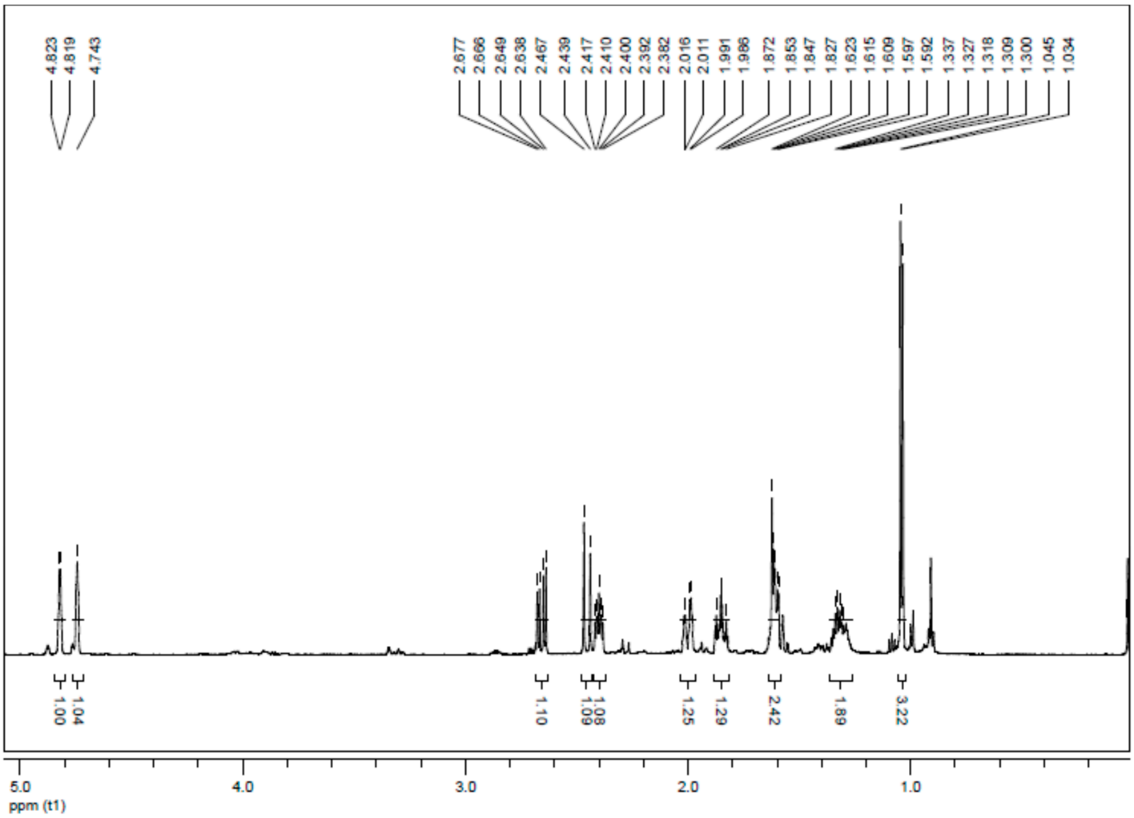

Figure S13. ^{1}H-NMR (300 MHz, $CDCl_3$) spectrum of iodolactone **5a**.

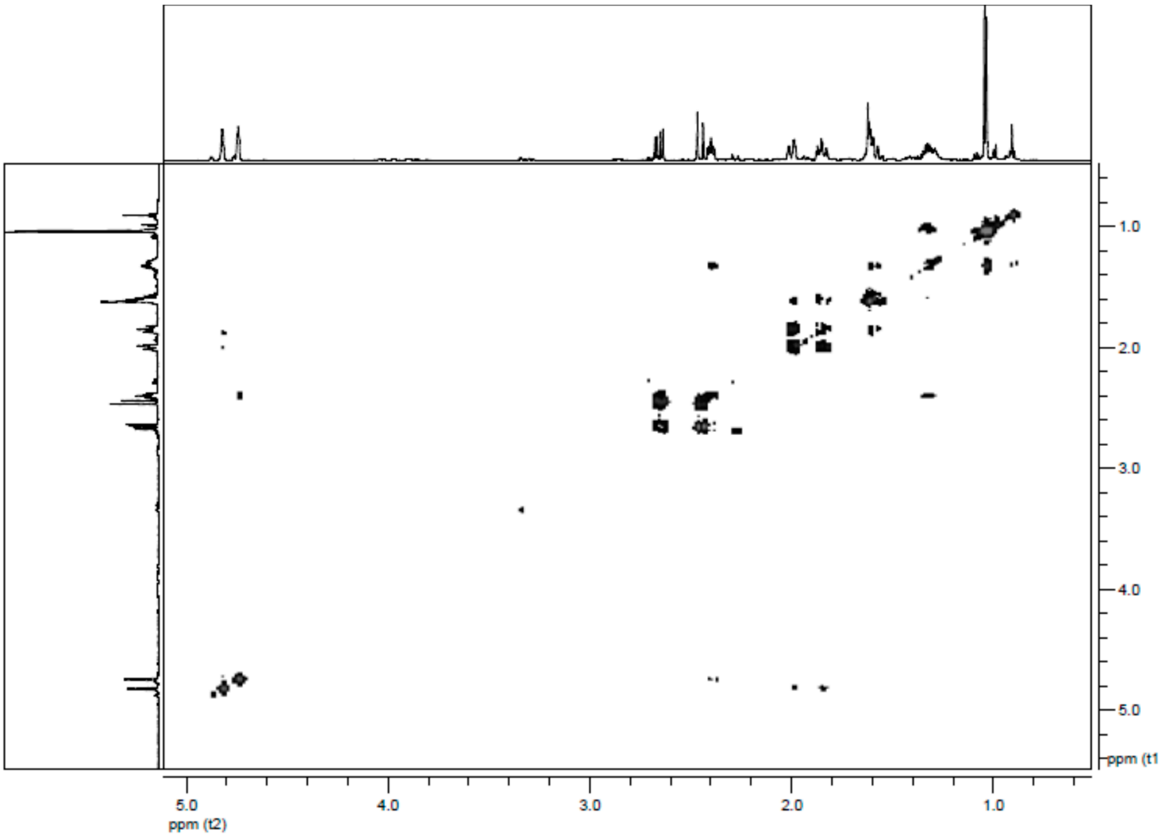

Figure S14. COSY (300 MHz, $CDCl_3$) spectrum of of iodolactone **5a**.

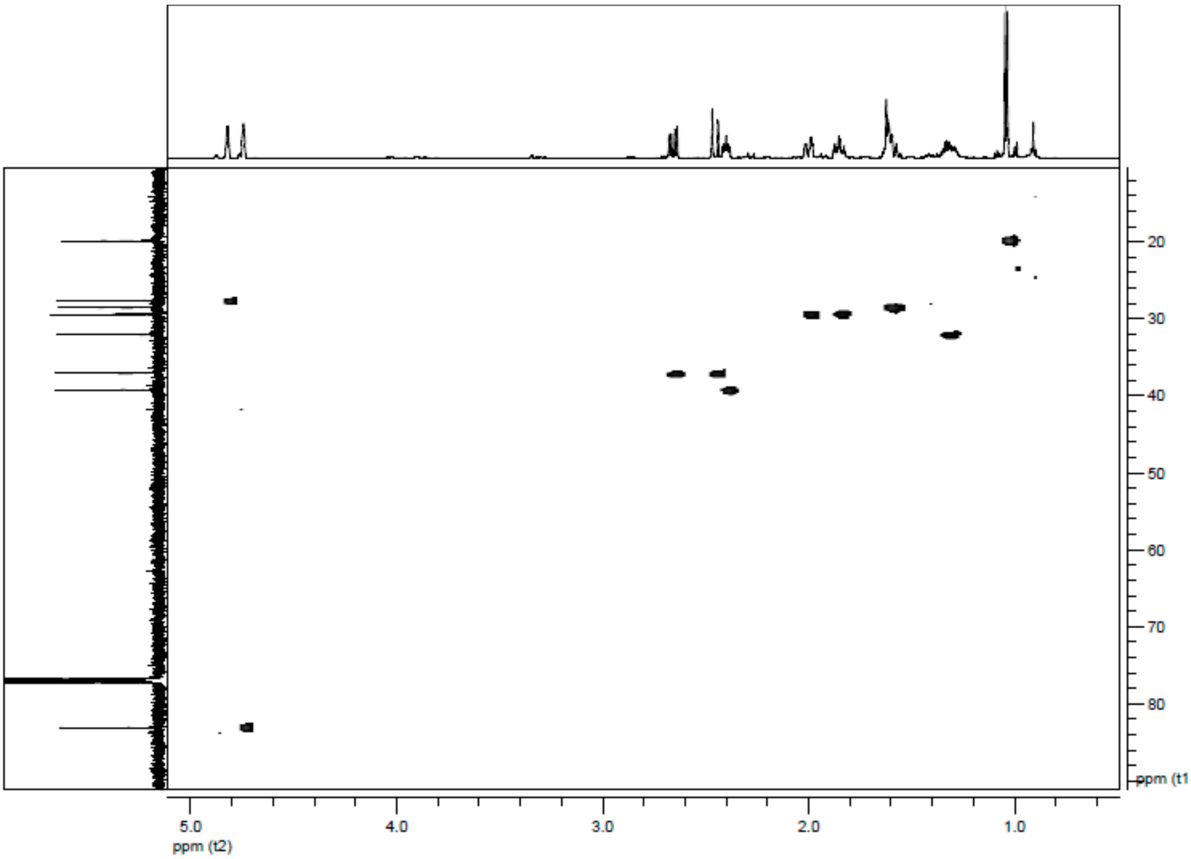

Figure S15. HMQC (300 MHz, $CDCl_3$) spectrum of of iodolactone **5a**.

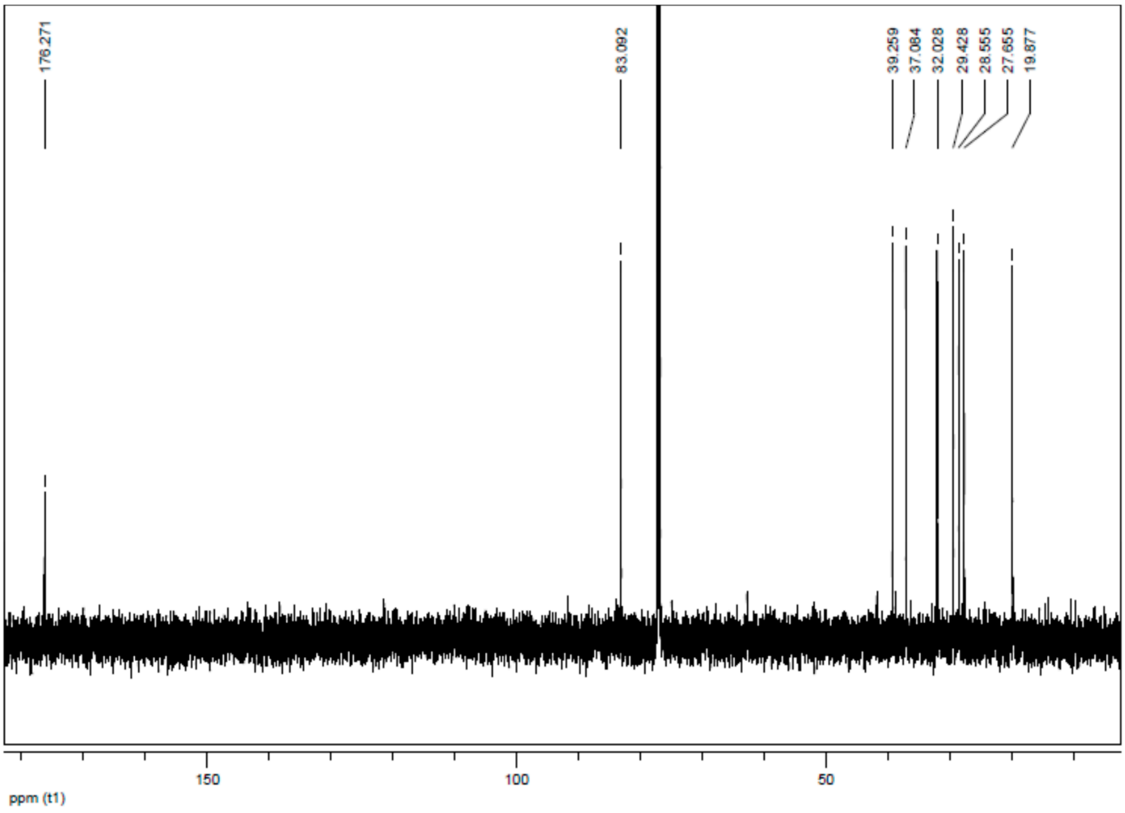

Figure S16. ^{13}C-NMR (75.5 MHz, $CDCl_3$) spectrum of of iodolactone **5a**.

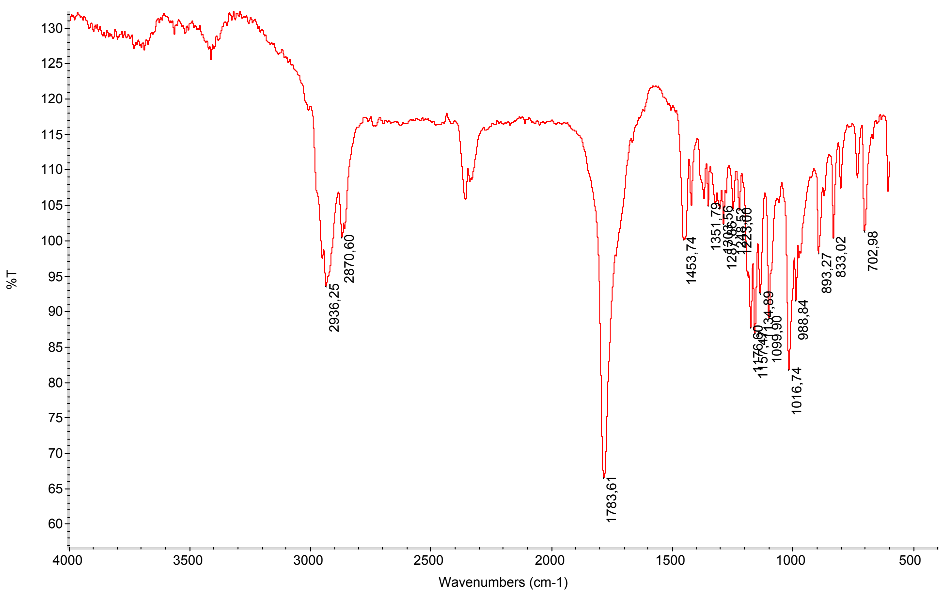

Figure S17. IR spectrum of of iodolactone **5a**.

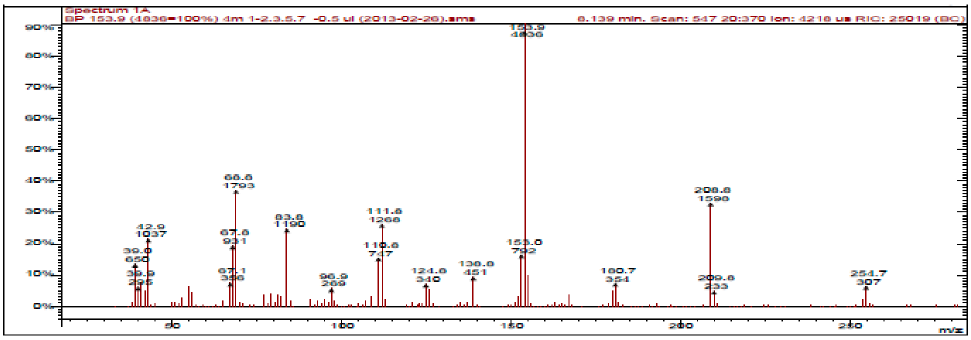

Figure S18. GC-MS spectrum of of iodolactone **5a**.

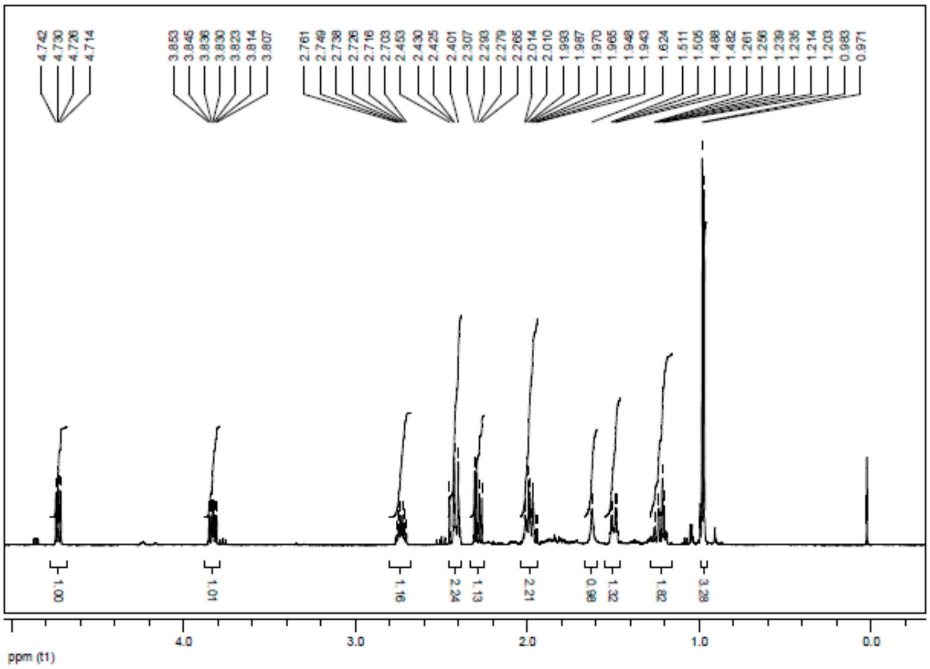

Figure S19. ^{1}H-NMR (300 MHz, $CDCl_3$) spectrum of iodolactone **6a**.

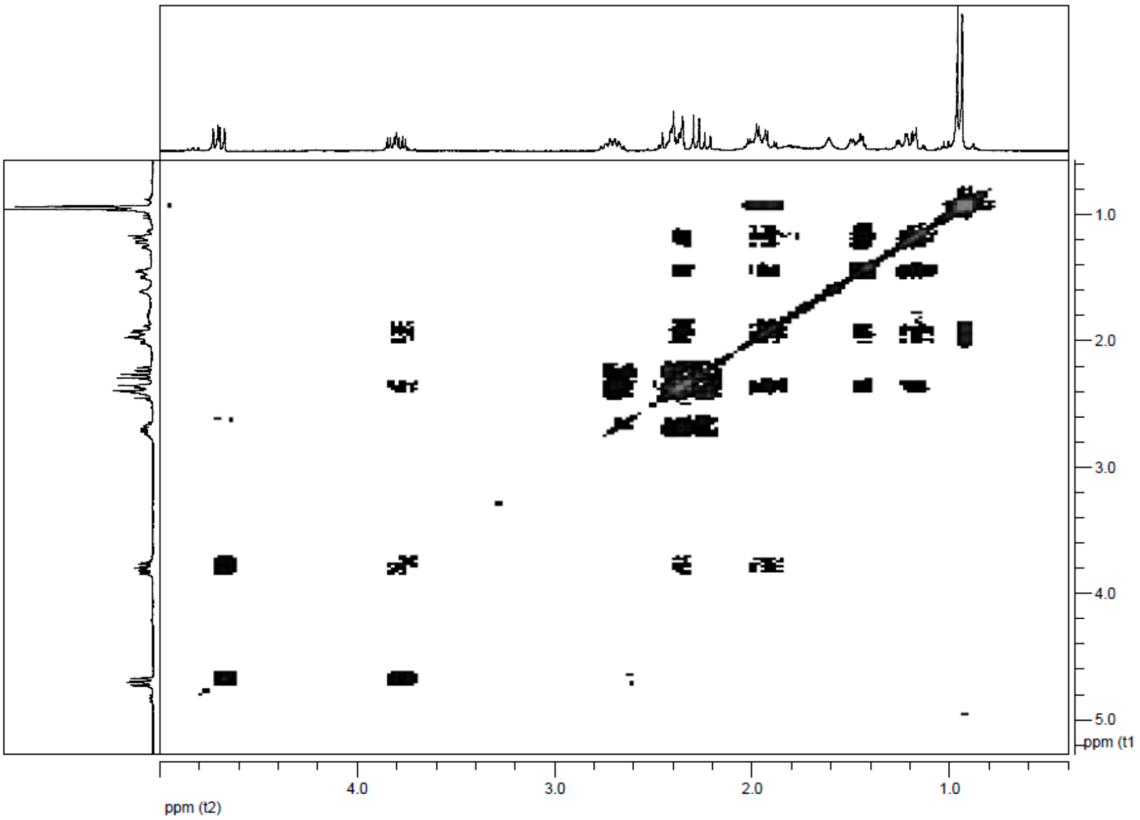

Figure S20. COSY (300 MHz, $CDCl_3$) spectrum of of iodolactone **6a**.

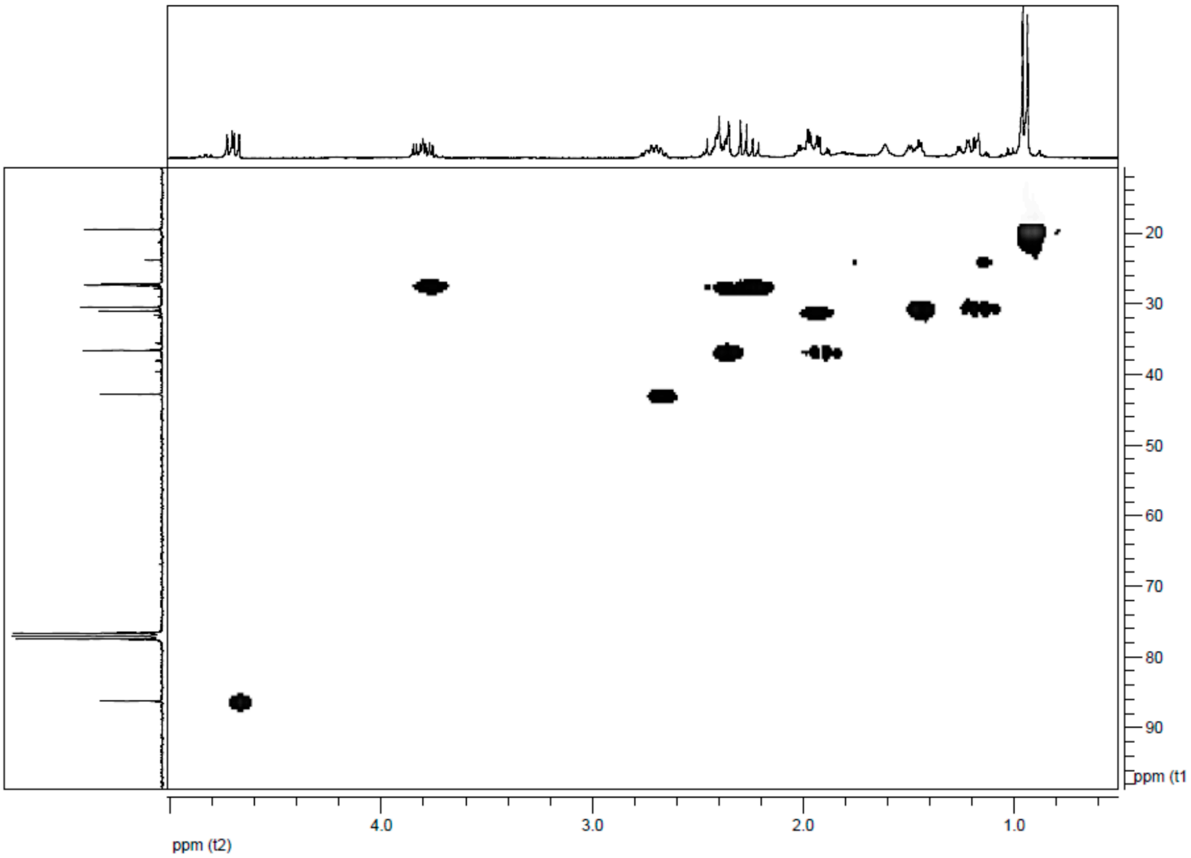

Figure S21. HMQC (300 MHz, $CDCl_3$) spectrum of of iodolactone **6a**.

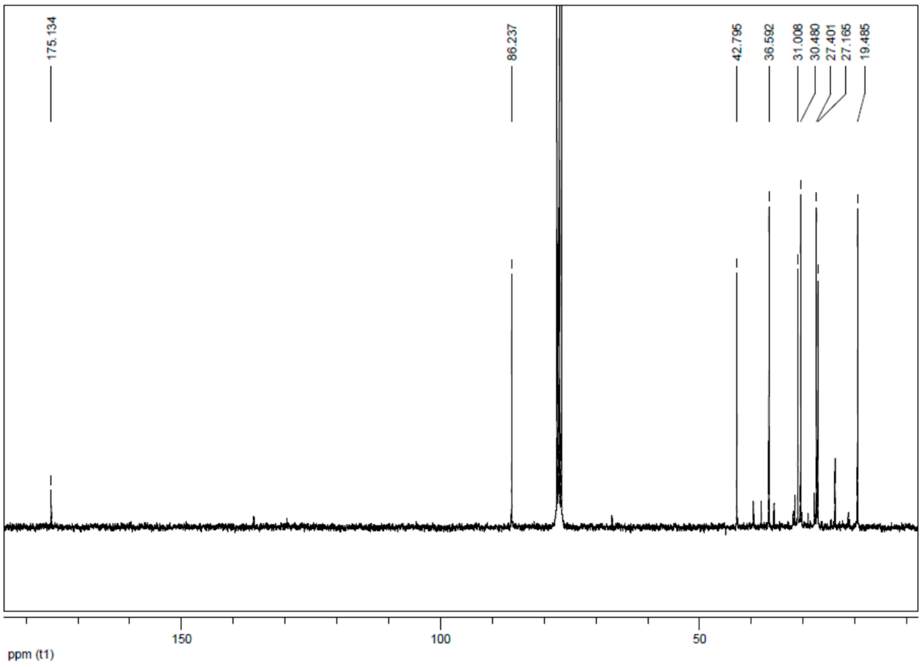

Figure S22. ^{13}C-NMR (75.5 MHz, $CDCl_3$) spectrum of iodolactone **6a**.

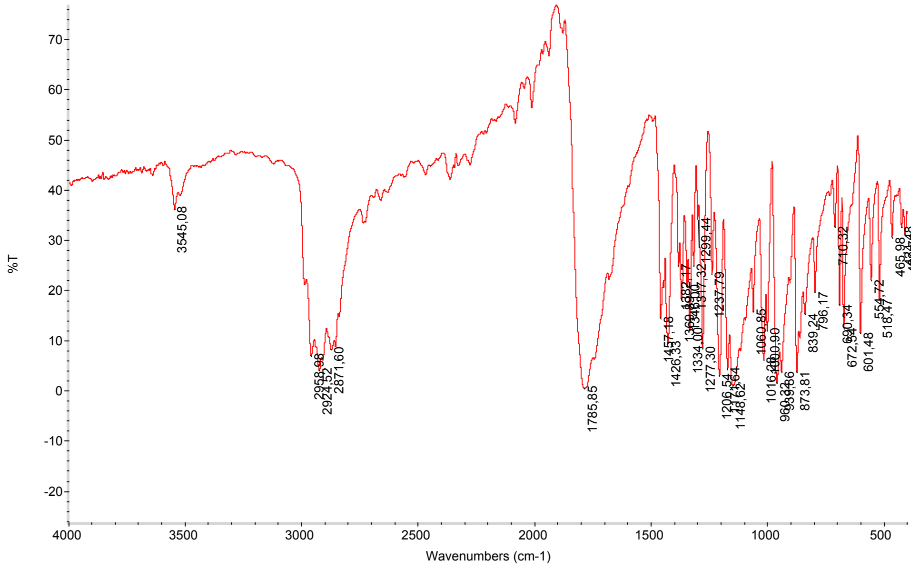

Figure S23. IR spectrum of iodolactone **6a**.

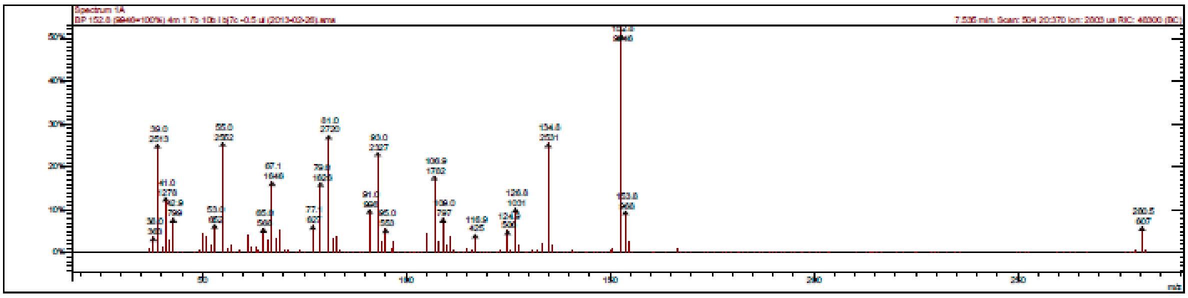

Figure S24. GC-MS spectrum of of iodolactone **6a**.

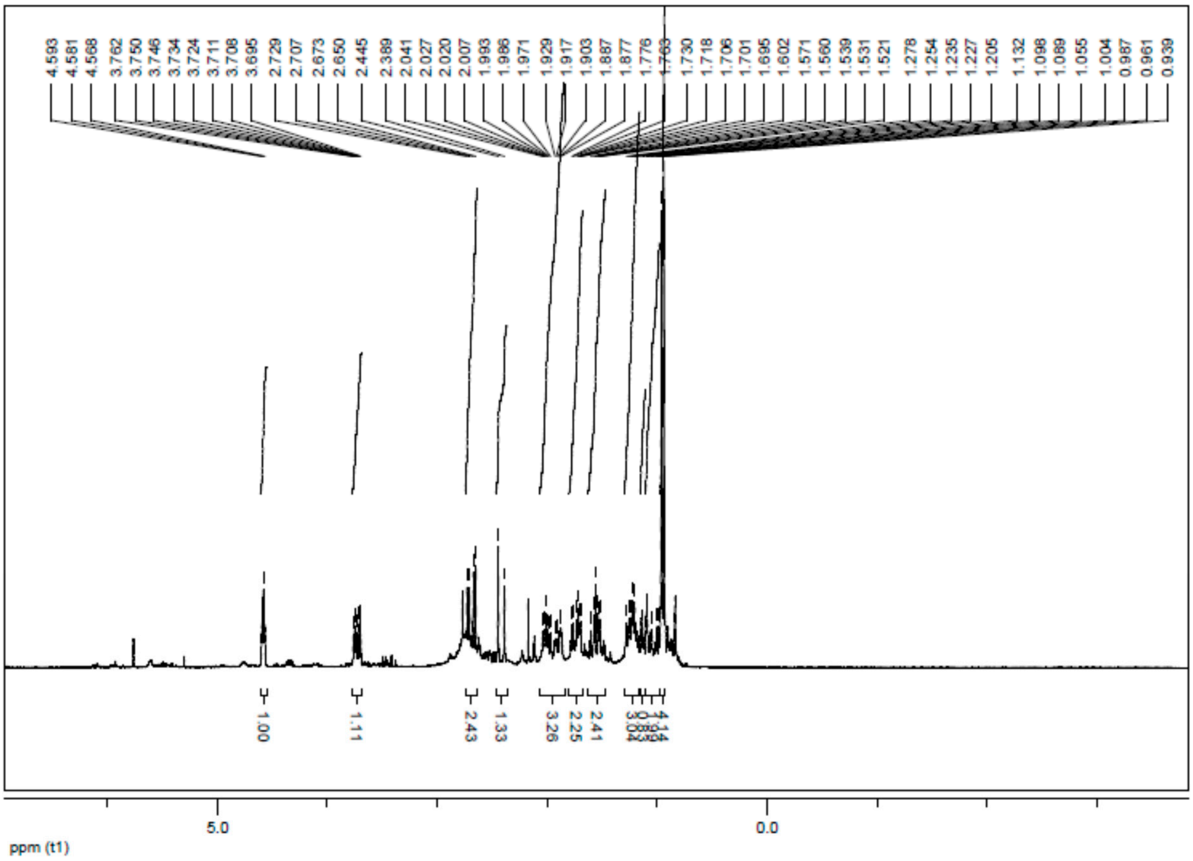

Figure S25. ^{1}H-NMR (300 MHz, $CDCl_3$) spectrum of hydroxylactone **7a**.

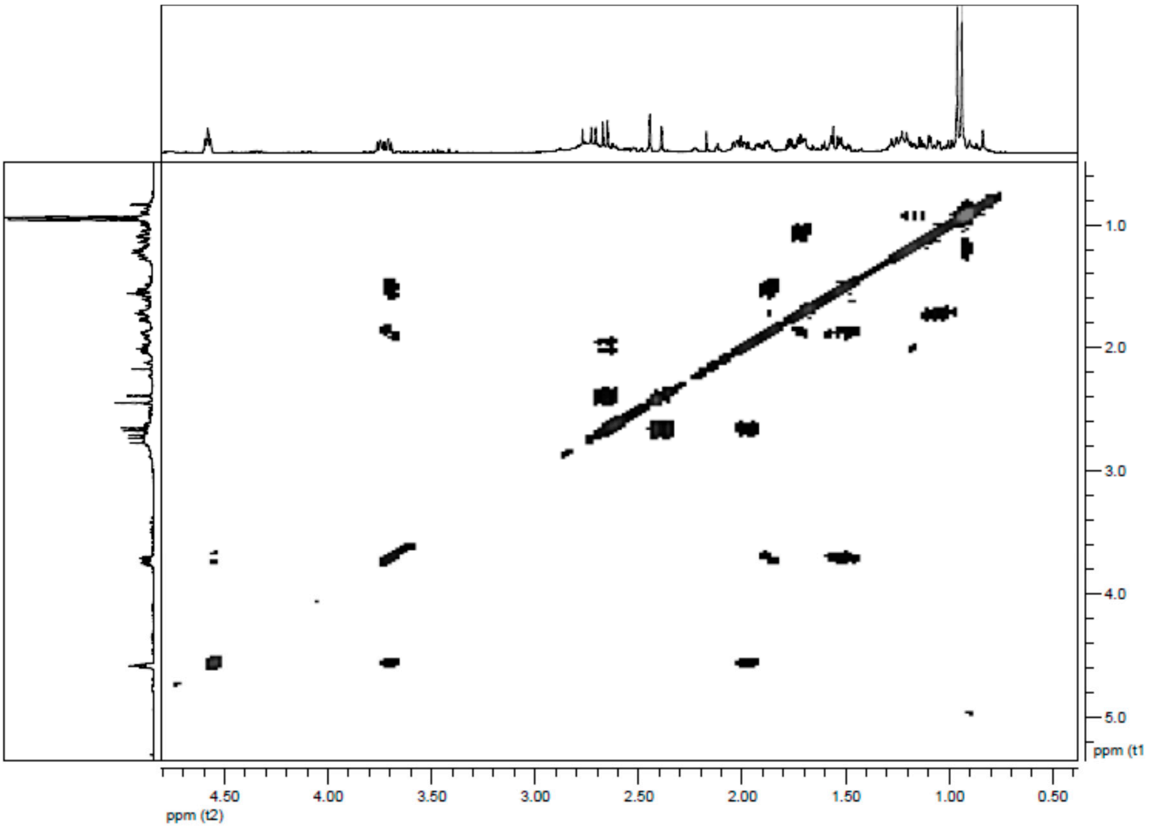

Figure S26. COSY (300 MHz, $CDCl_3$) spectrum of hydroxylactone **7a**.

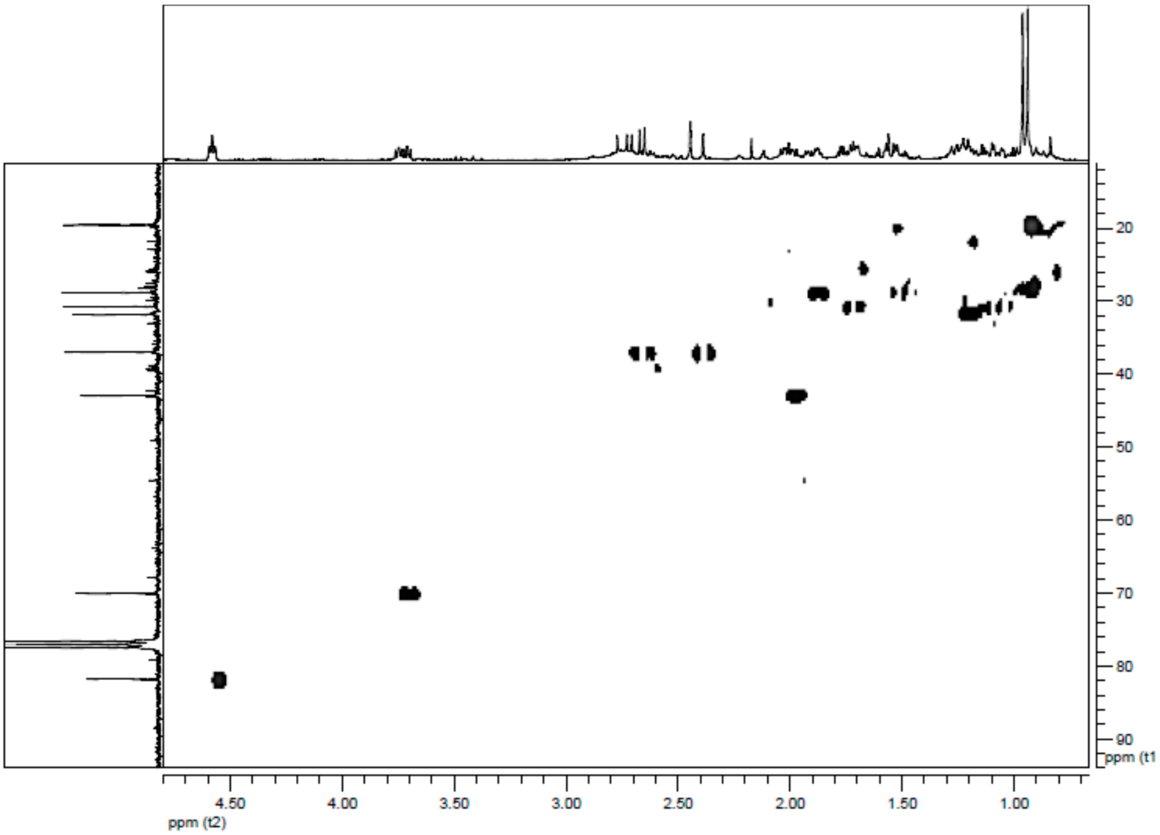

Figure S27. HMQC (300 MHz, $CDCl_3$) spectrum of hydroxylactone **7a**.

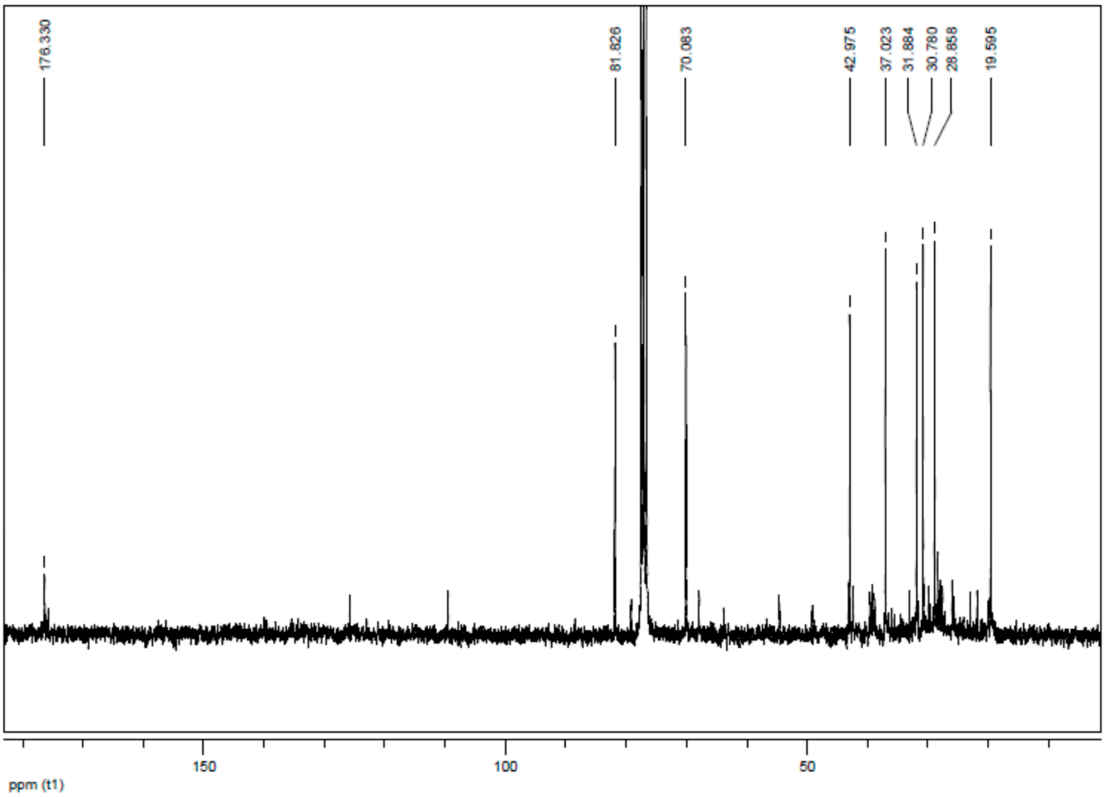

Figure S28. ^{13}C-NMR (75.5 MHz, $CDCl_3$) spectrum of iodolactone **7a**.

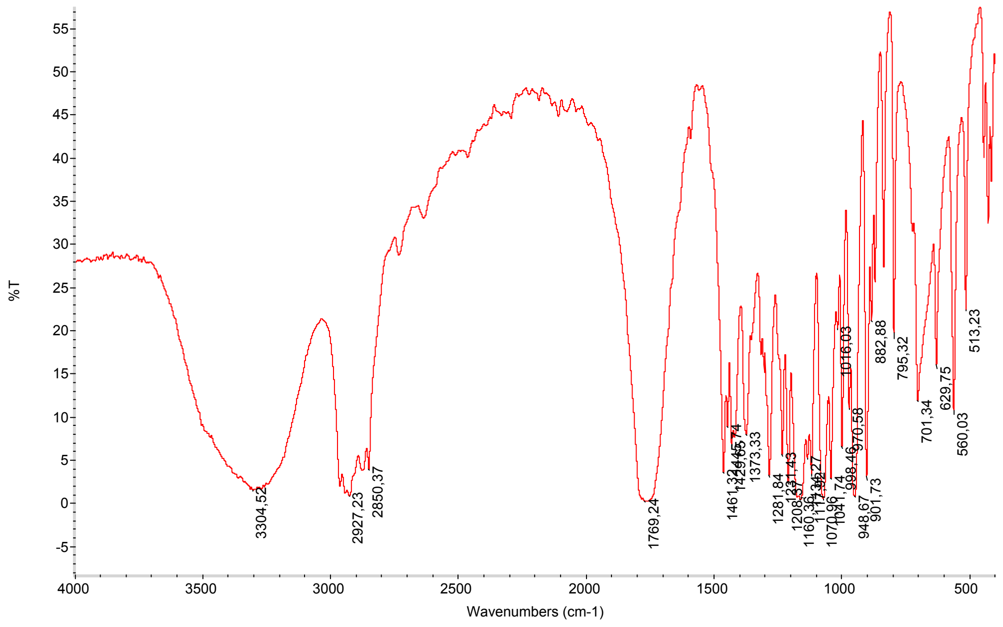

Figure S29. IR spectrum of hydroxylactone **7a**.

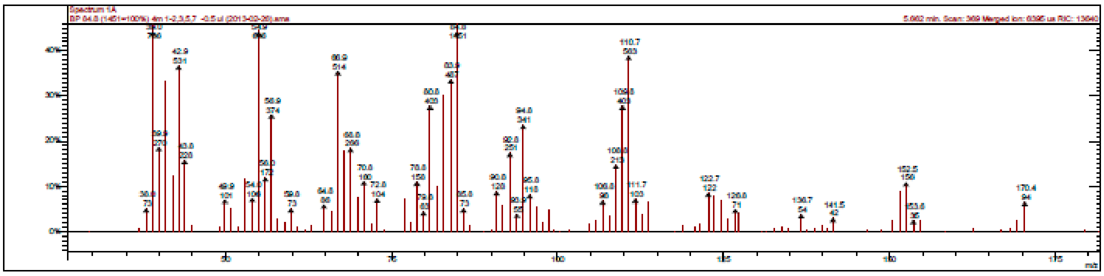

Figure S30. GC-MS spectrum of hydroxylactone **7a**.

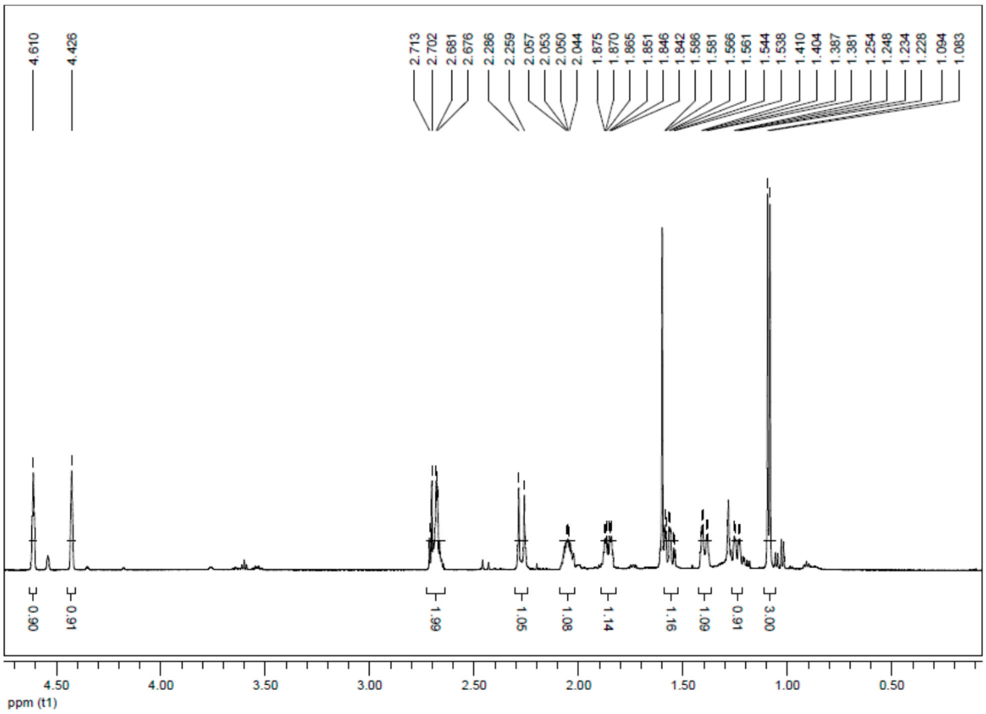

Figure S31. ^{1}H-NMR (300 MHz, $CDCl_3$) spectrum of chlorolactone **3b**.

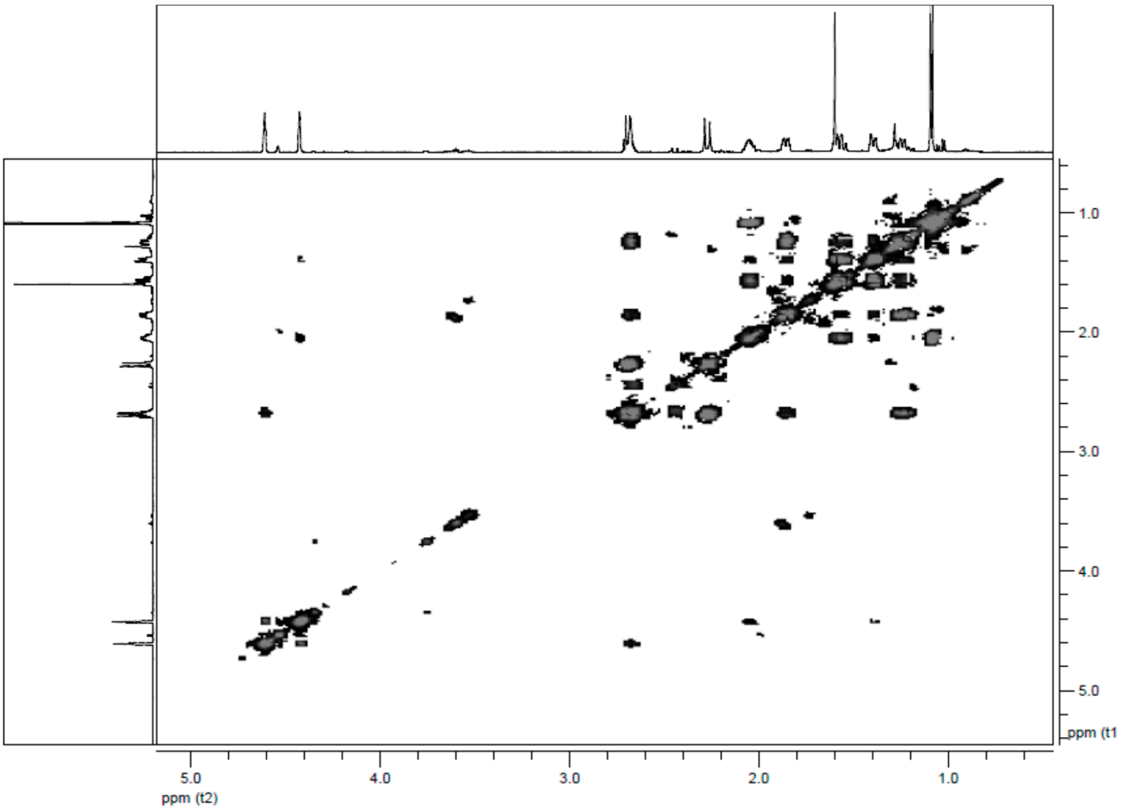

Figure S32. COSY (300 MHz, $CDCl_3$) spectrum of chlorolactone **3b**.

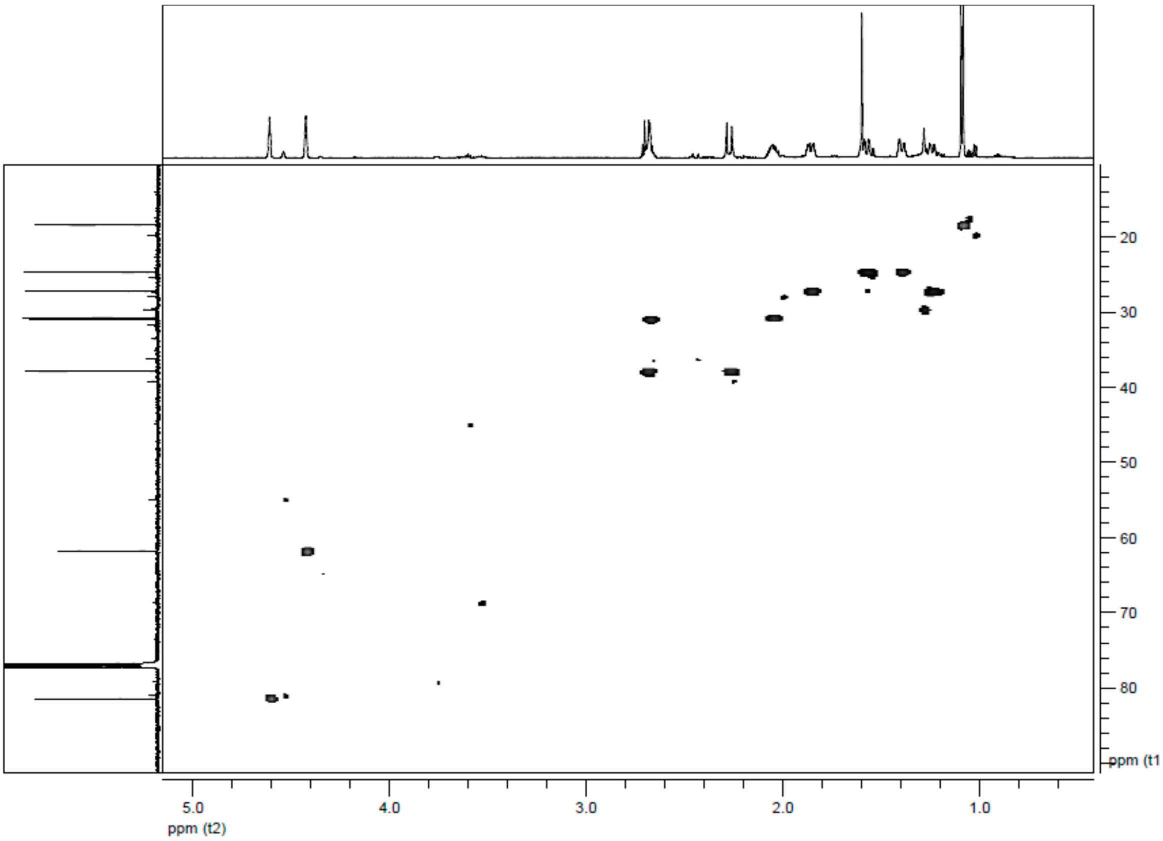

Figure S33. HMQC (300 MHz, $CDCl_3$) spectrum of chlorolactone **3b**.

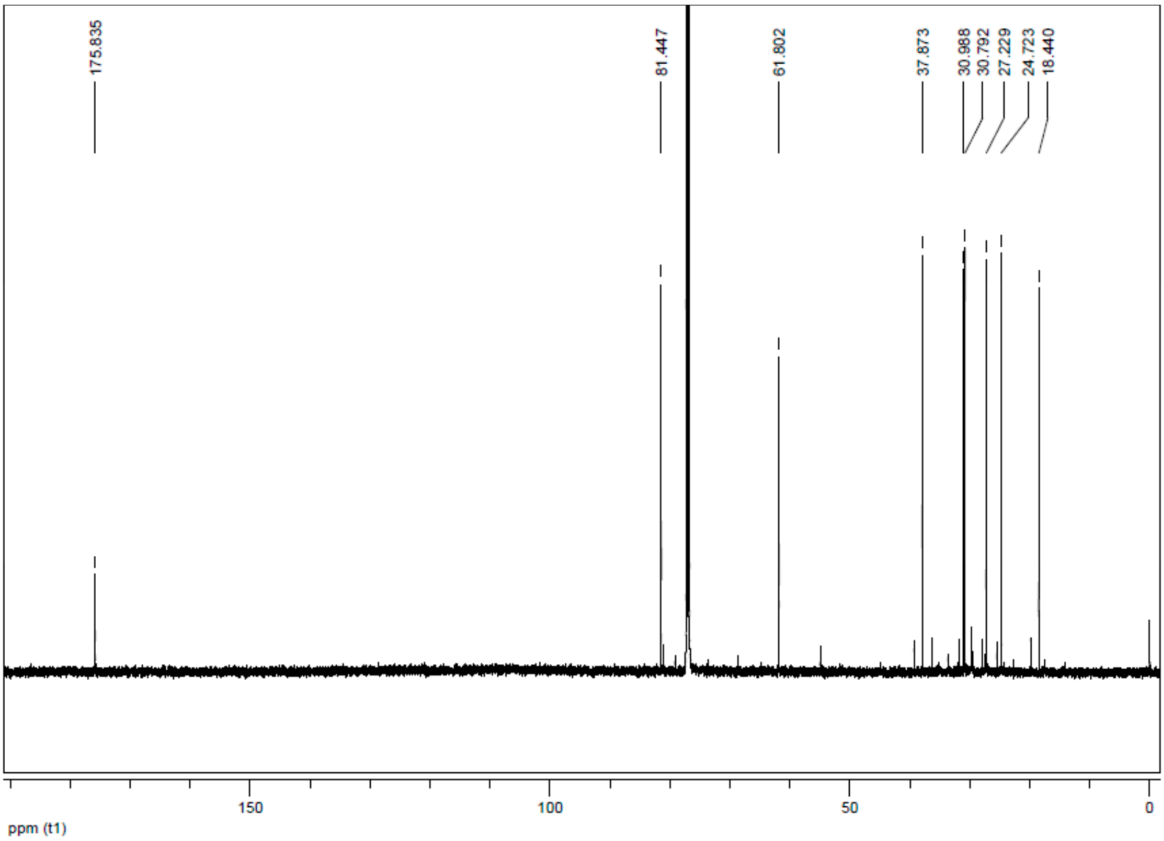

Figure S34. ^{13}C-NMR (75.5 MHz, $CDCl_3$) spectrum of chlorolactone **3b**.

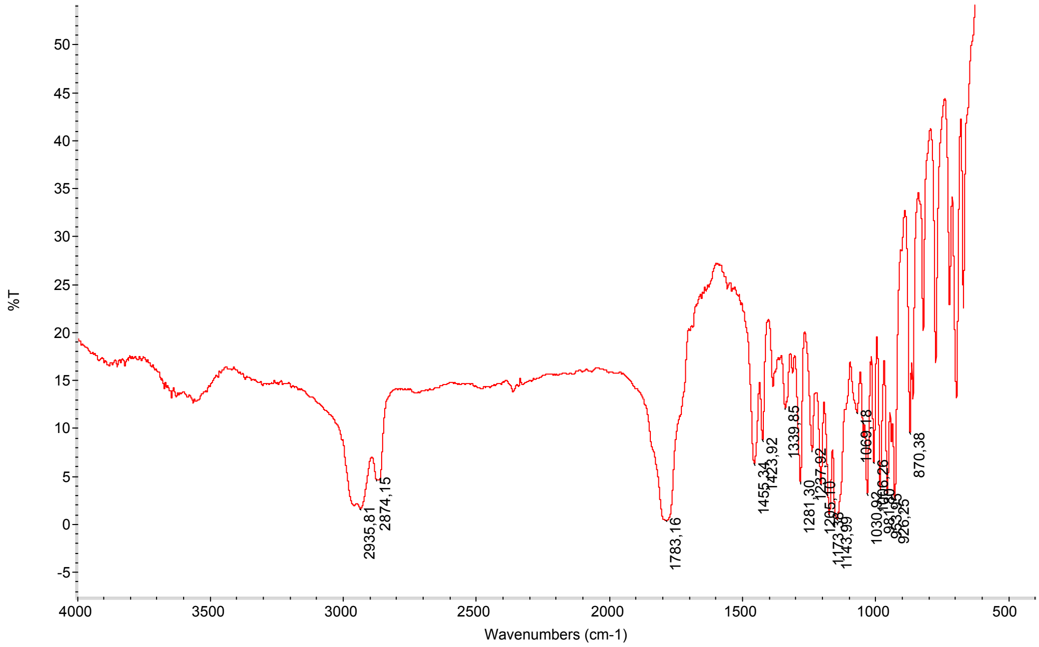

Figure S35. IR spectrum of chlorolactone **3b**.

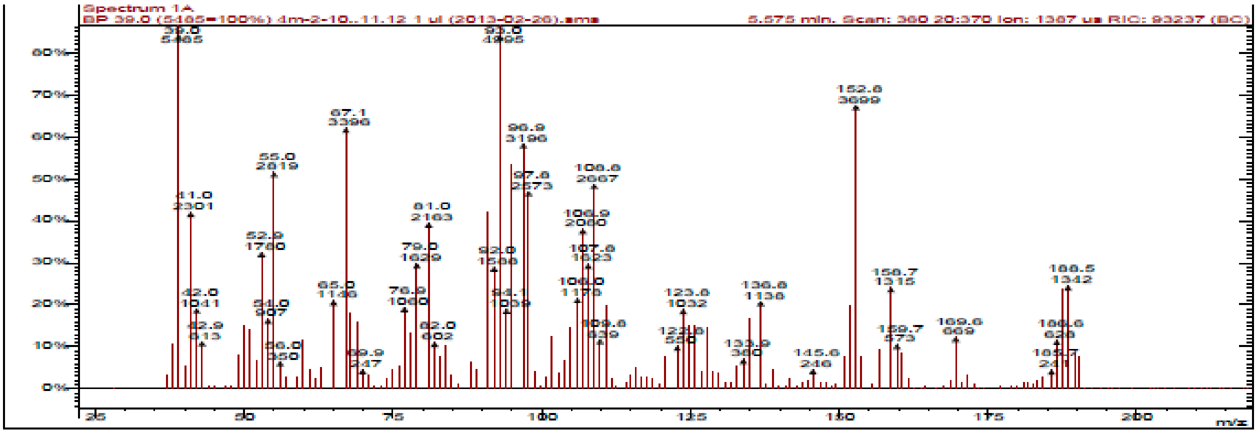

Figure S36. GC-MS spectrum of chlorolactone **3b**.

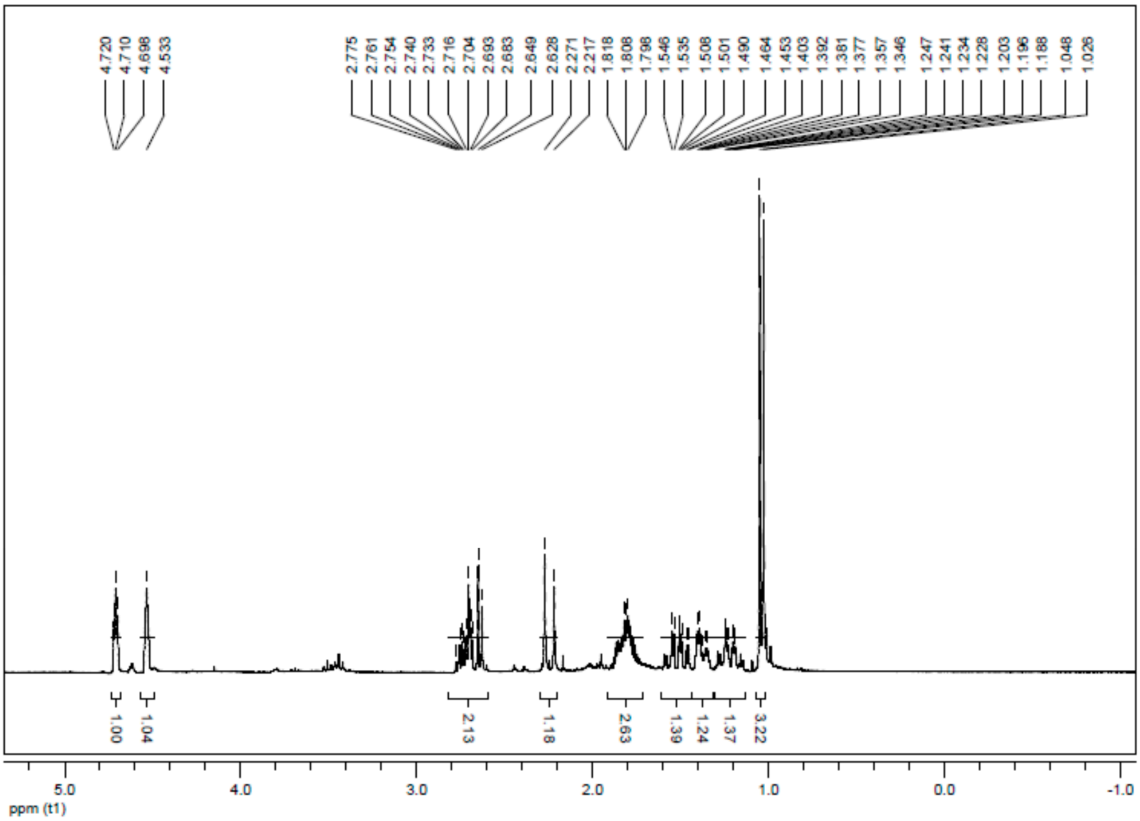

Figure S37. ^{1}H-NMR (300 MHz, $CDCl_3$) spectrum of bromolactone **4b**.

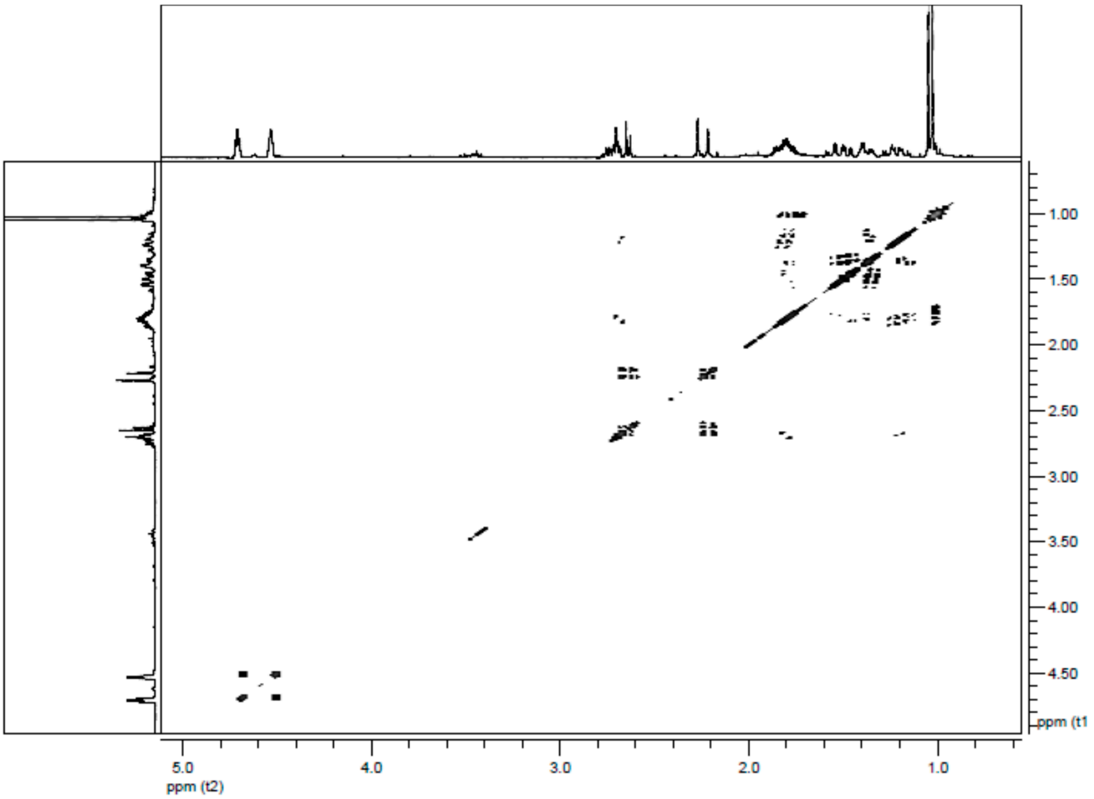

Figure S38. COSY (300 MHz, $CDCl_3$) spectrum of bromolactone **4b**.

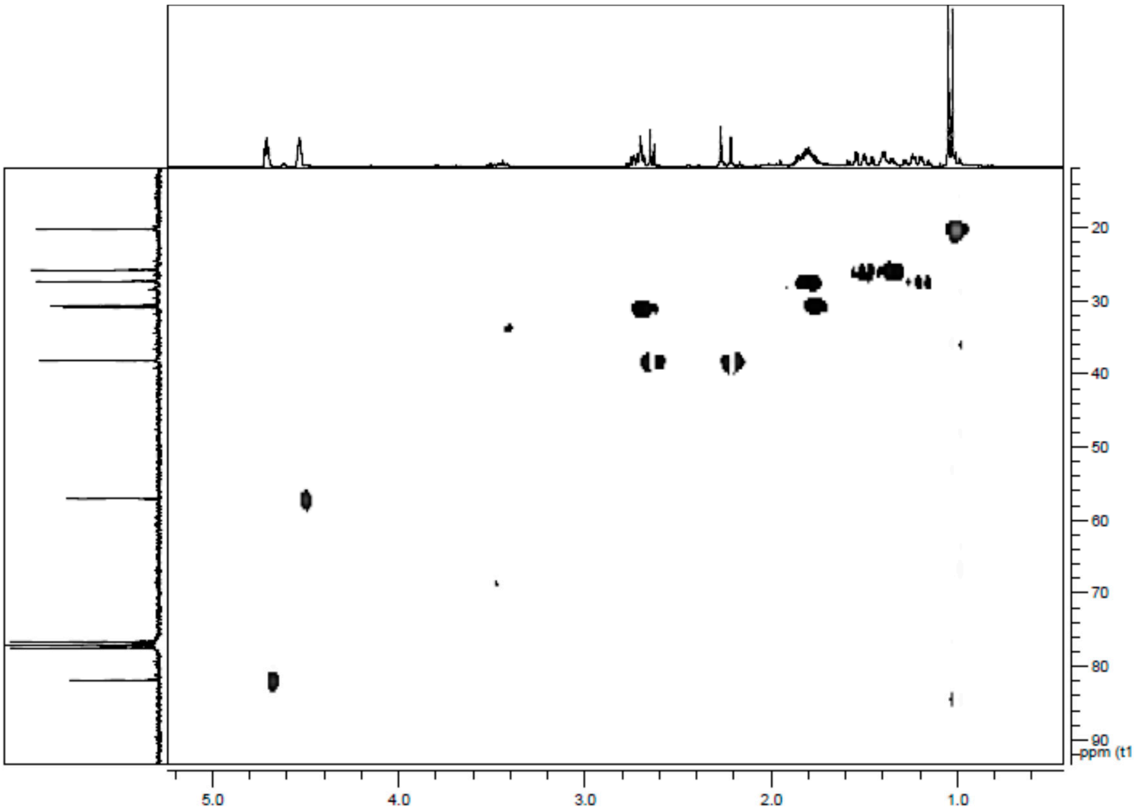

Figure S39. HMQC (300 MHz, $CDCl_3$) spectrum of bromolactone **4b**.

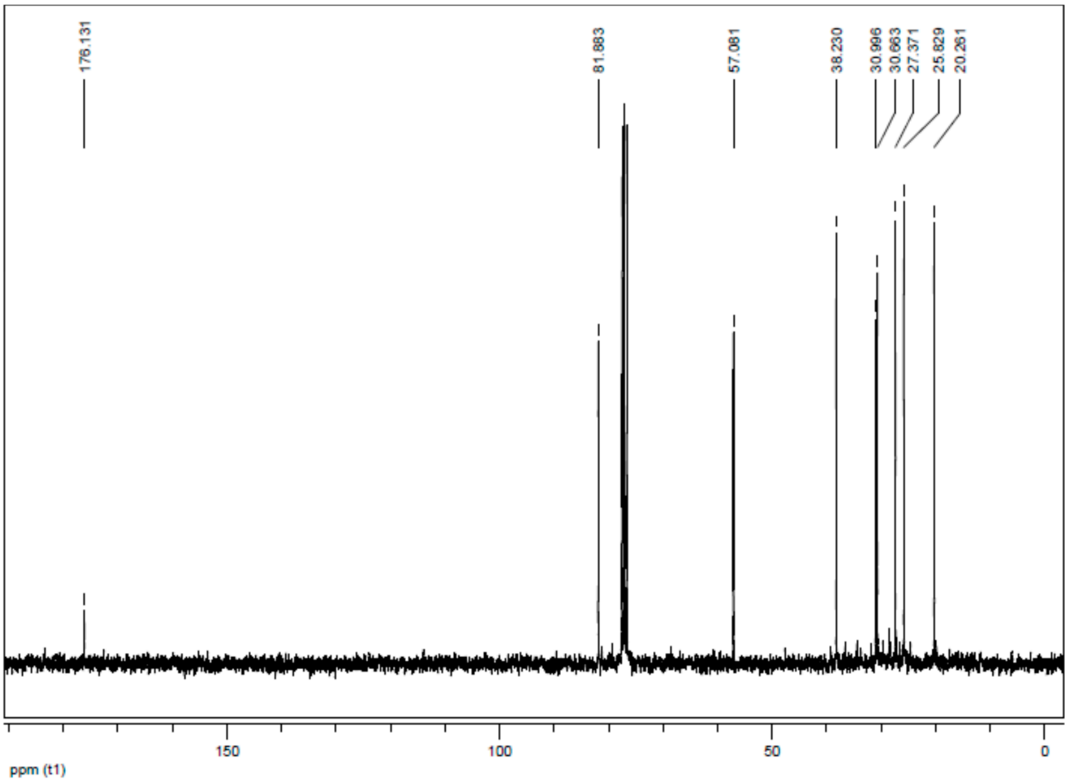

Figure S40. ^{13}C-NMR (75.5 MHz, $CDCl_3$) spectrum of bromolactone **4b**.

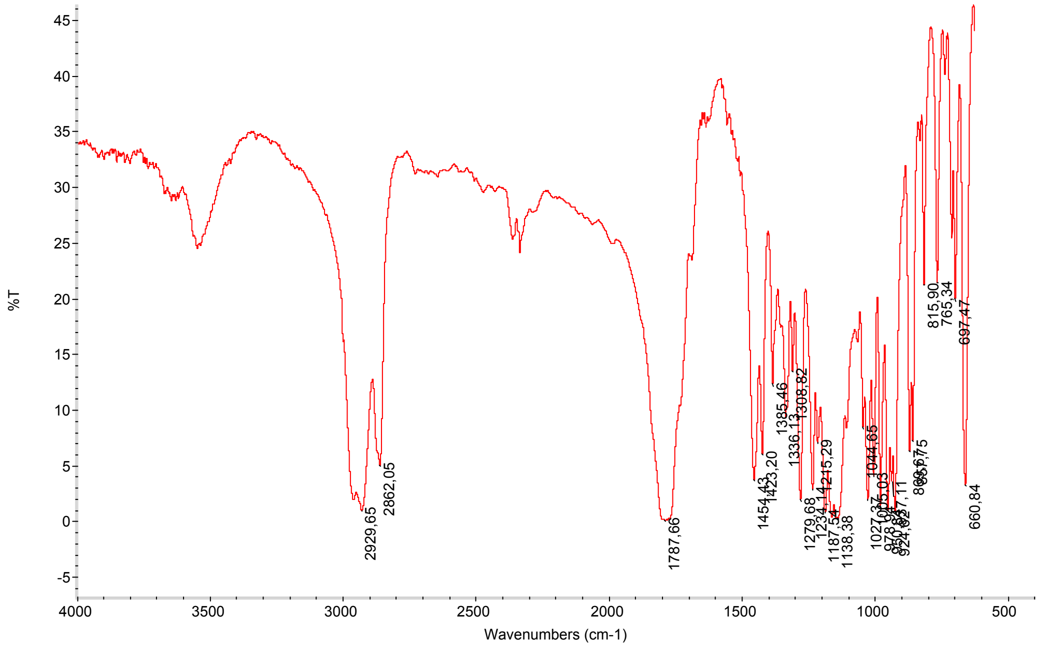

Figure S41. IR spectrum of bromolactone **4b**.

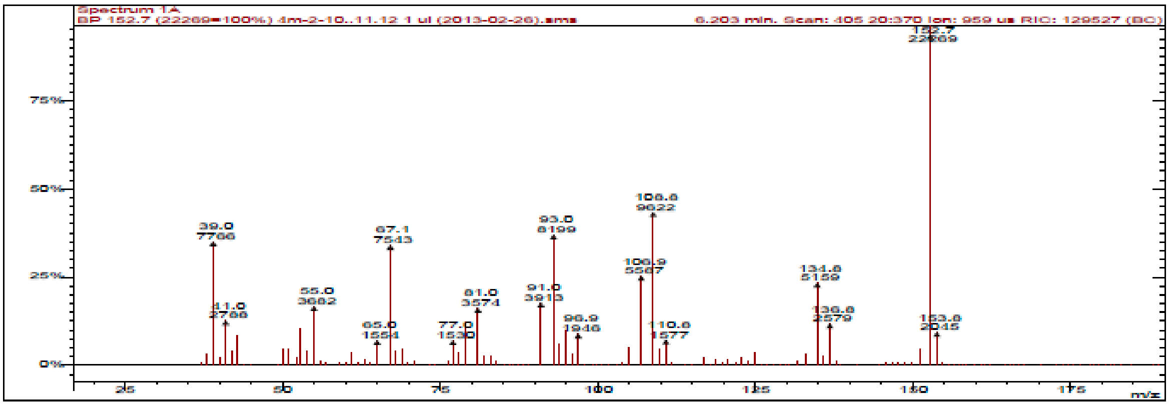

Figure S42. GC-MS spectrum of bromolactone **4b**.

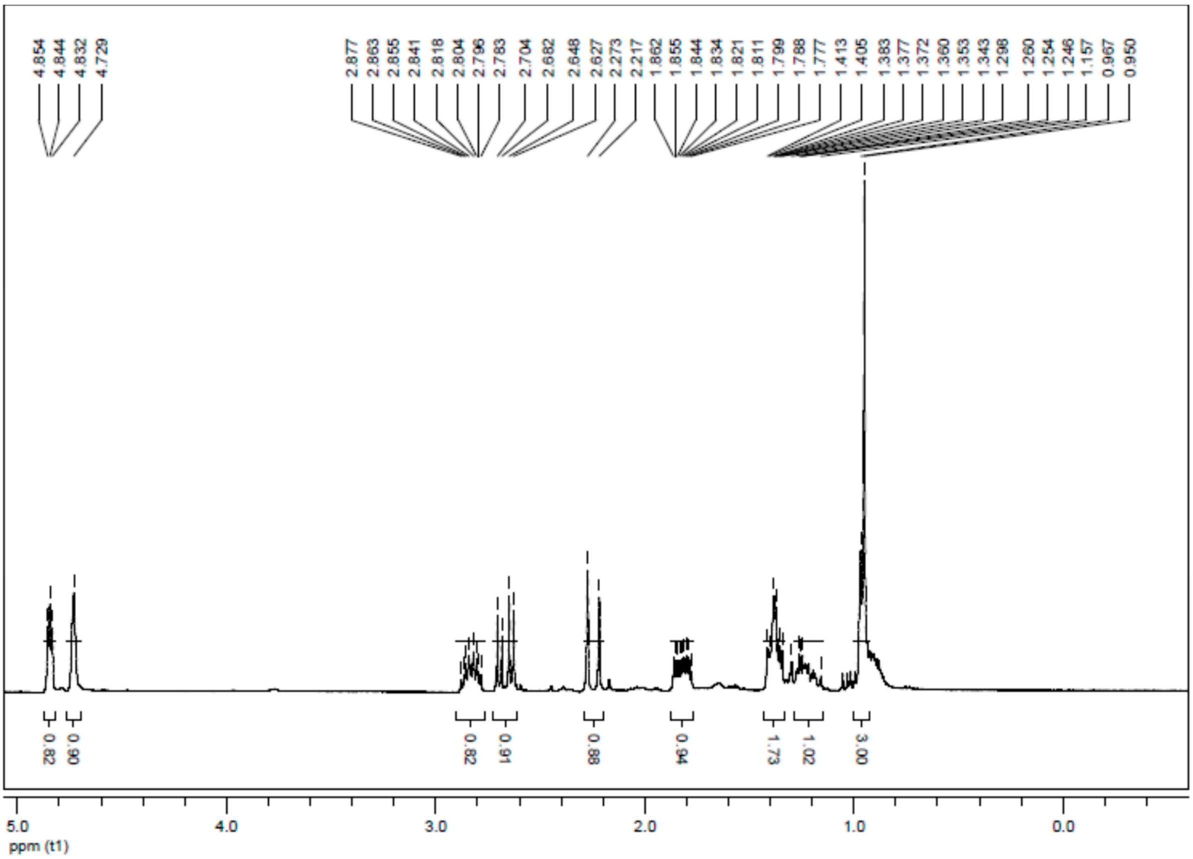

Figure S43. ^{1}H-NMR (300 MHz, $CDCl_3$) spectrum of iodolactone **5b**.

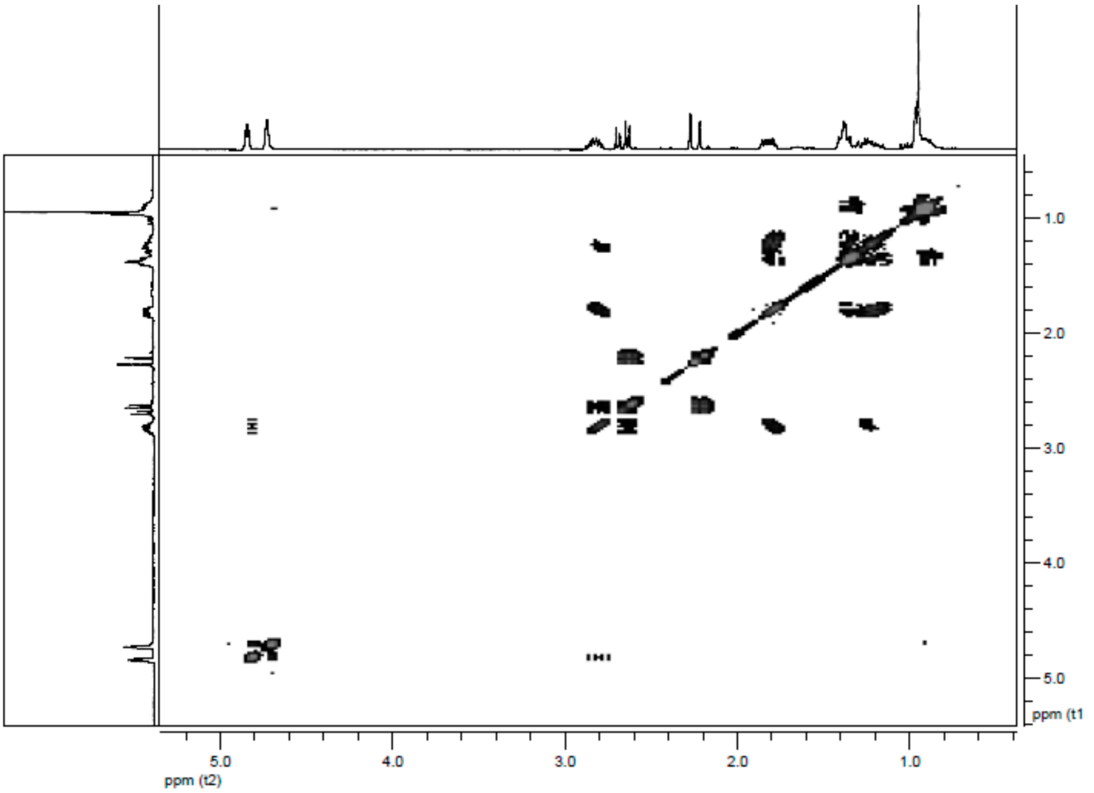

Figure S44. COSY (300 MHz, $CDCl_3$) spectrum of of iodolactone **5b**.

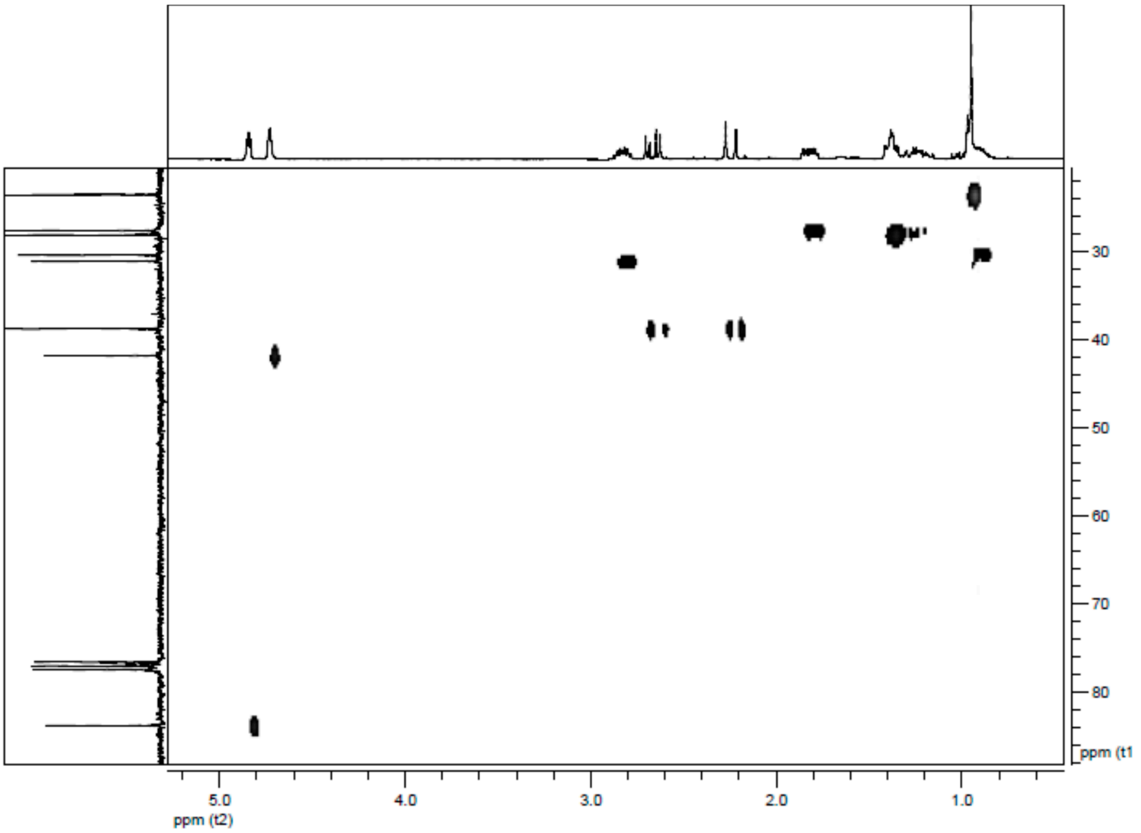

Figure S45. HMQC (300 MHz, $CDCl_3$) spectrum of of iodolactone **5b**.

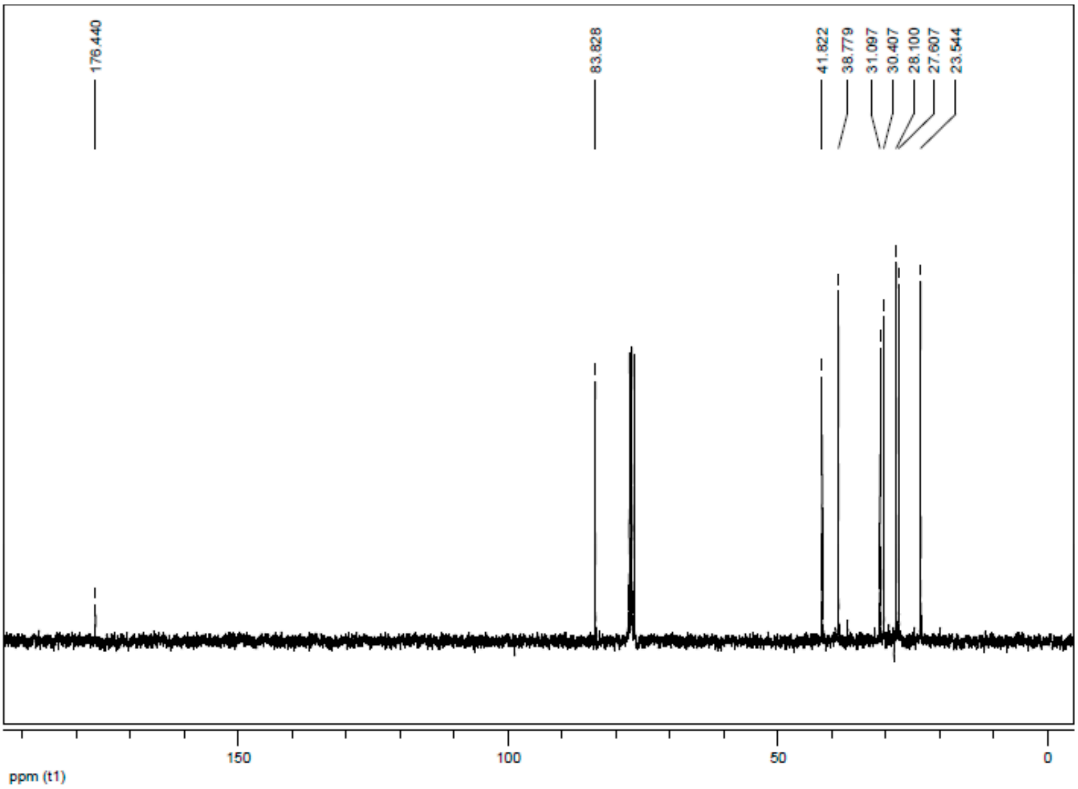

Figure S46. ^{13}C-NMR (75.5 MHz, $CDCl_3$) spectrum of of iodolactone **5b**.

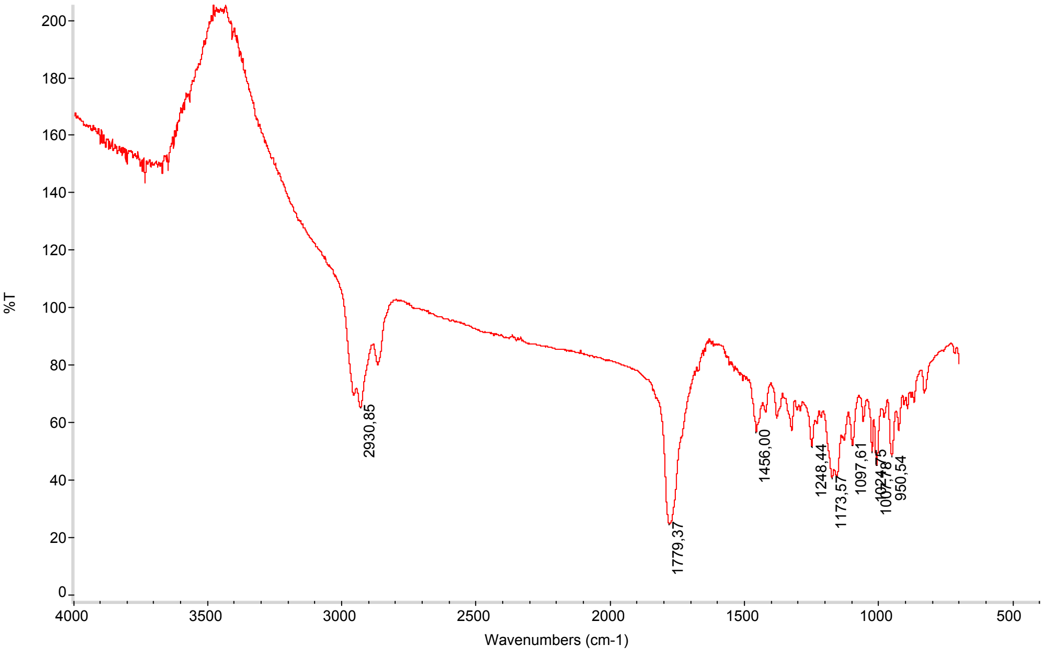

Figure S47. IR spectrum of of iodolactone **5b**.

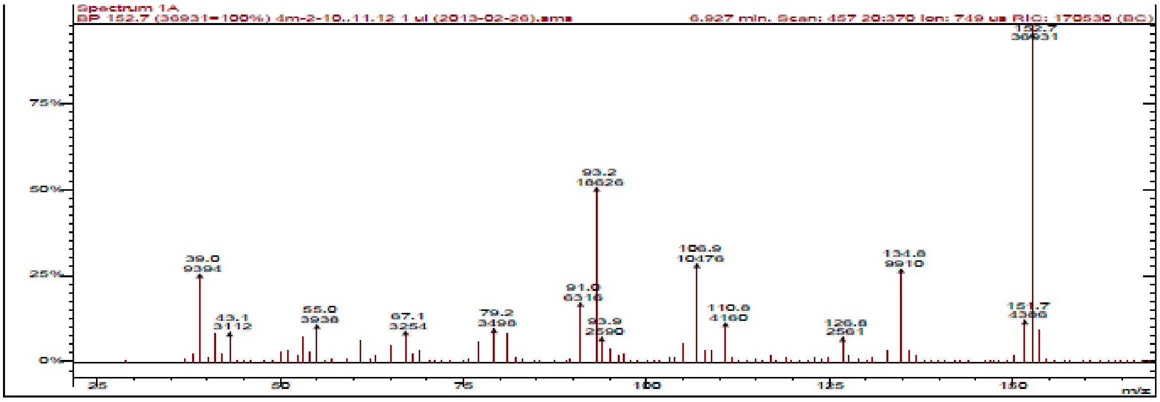

Figure S48. GC-MS spectrum of of iodolactone **5b**.

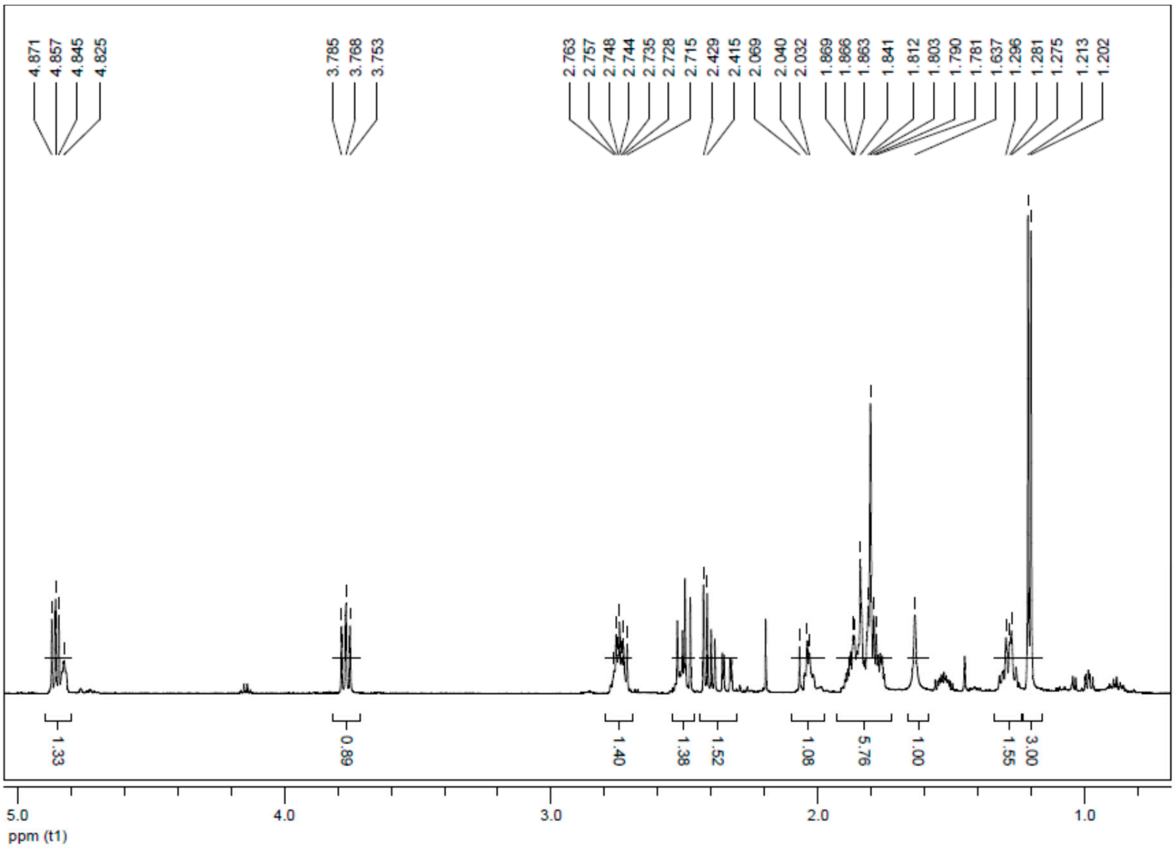

Figure S49. ^{1}H-NMR (300 MHz, $CDCl_3$) spectrum of iodolactone **6b**.

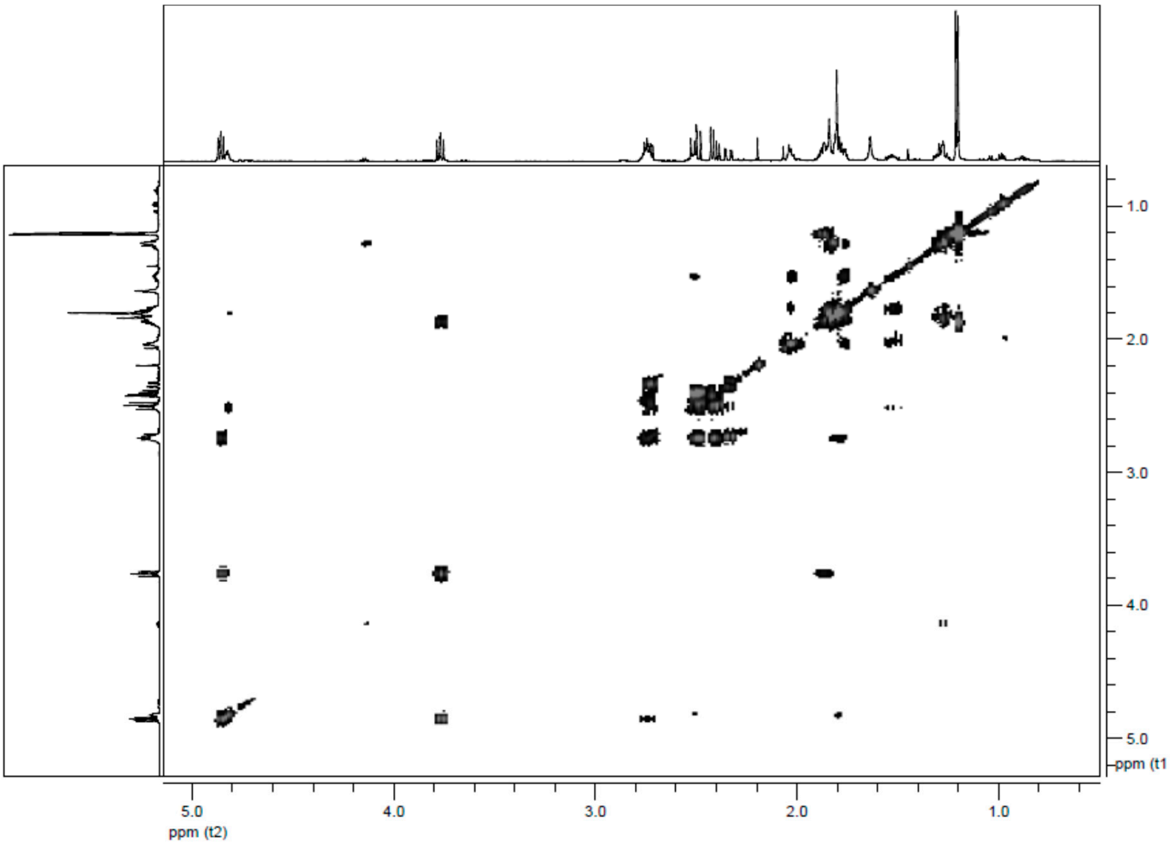

Figure S50. COSY (300 MHz, $CDCl_3$) spectrum of iodolactone **6b**.

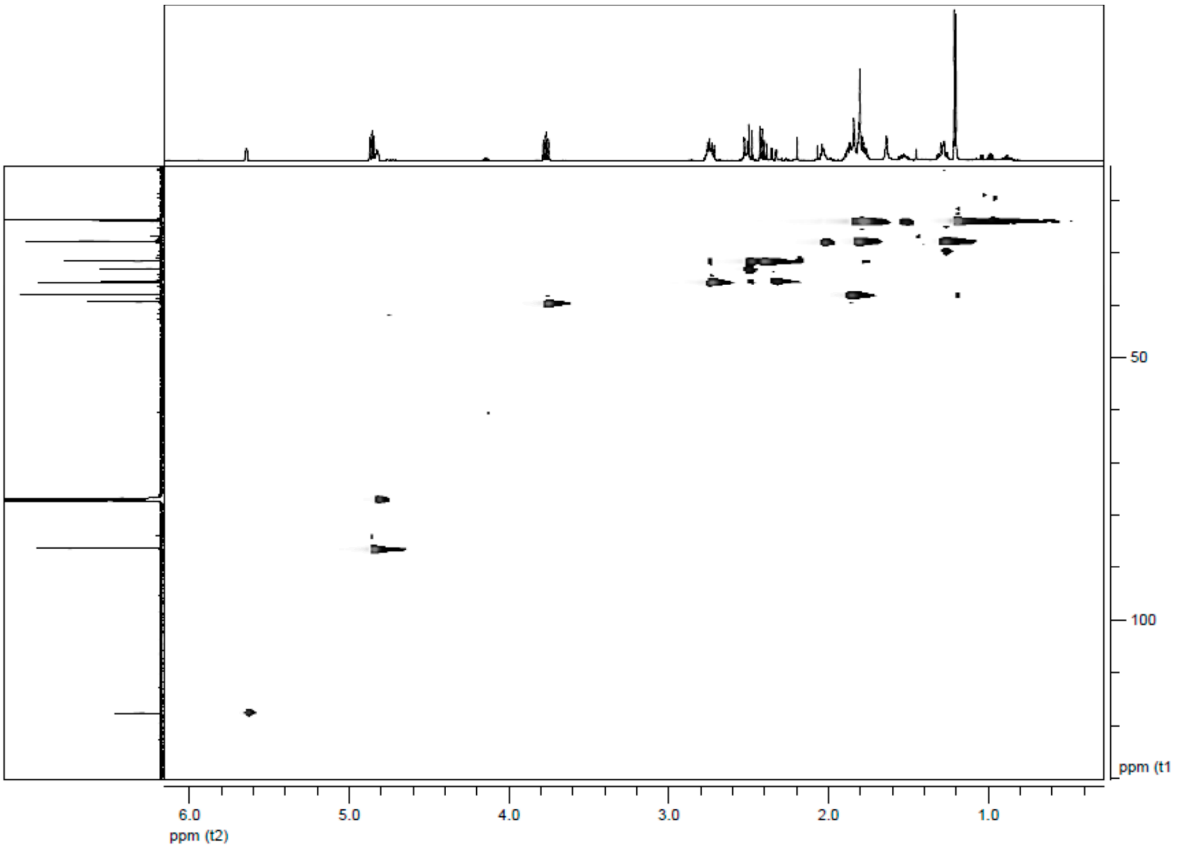

Figure S51. HMQC (300 MHz, $CDCl_3$) spectrum of iodolactone **6b**.

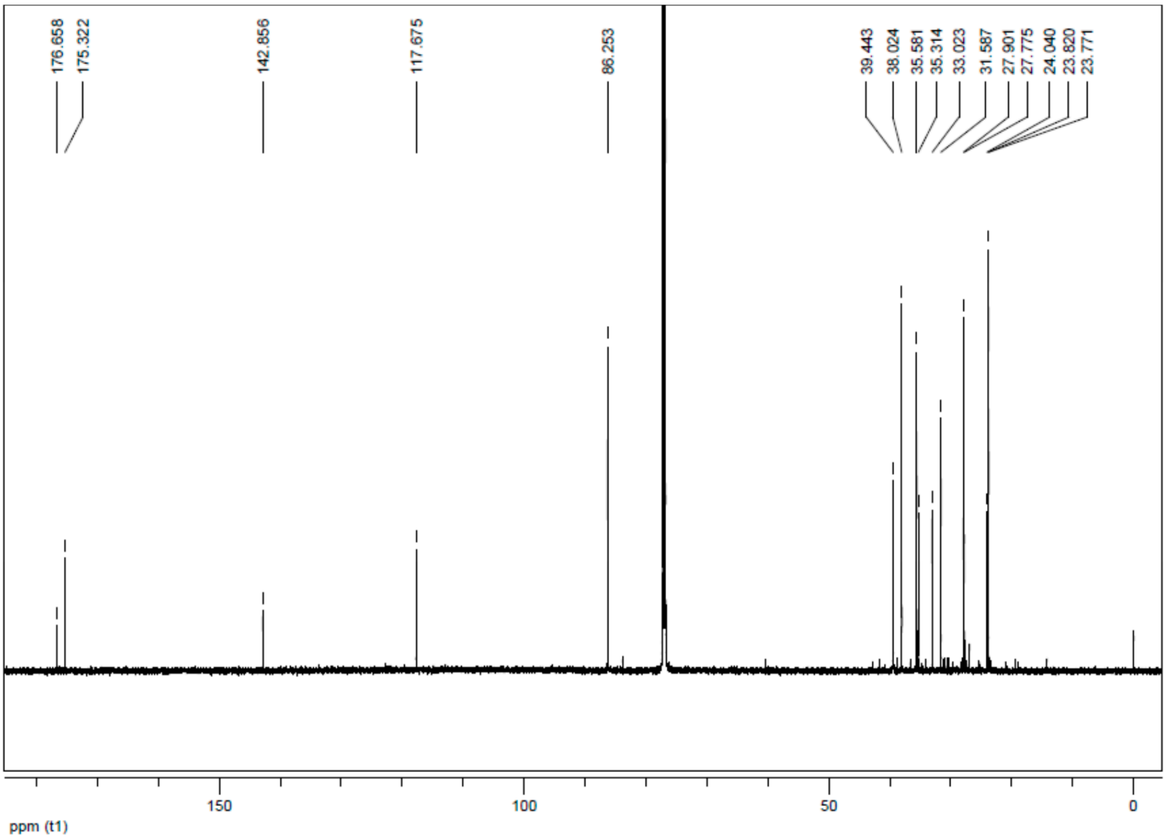

Figure S52. ^{13}C-NMR (75.5 MHz, $CDCl_3$) spectrum of of iodolactone **6b**.

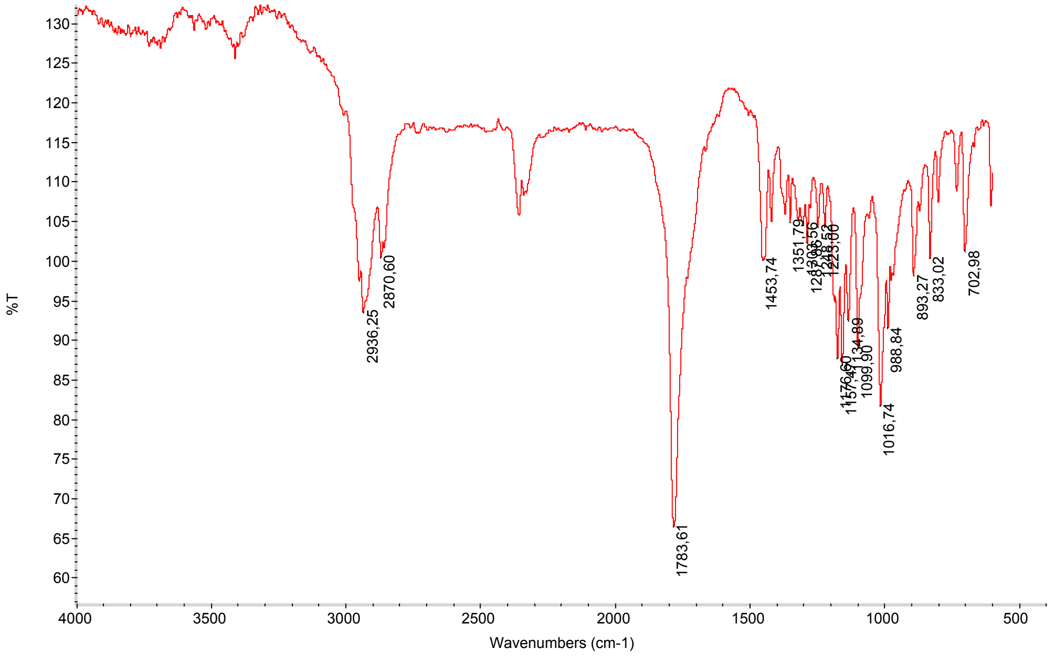

Figure S53. IR spectrum of of iodolactone **6b**.

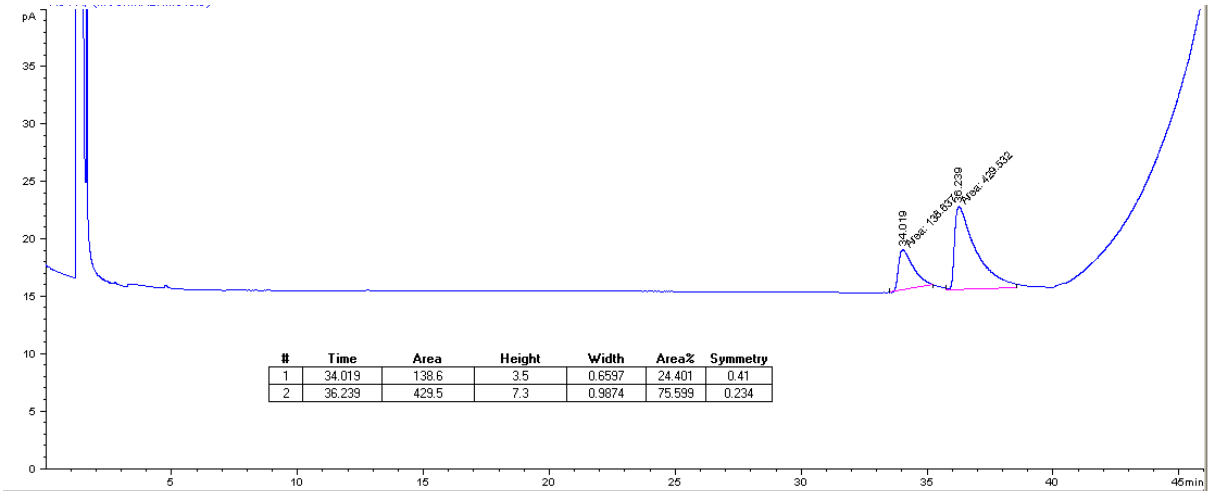

Figure S54. Chromatogram of hydroxylacton **7a** obtained from chlorolactone **3a** by F. culmorum AM10.

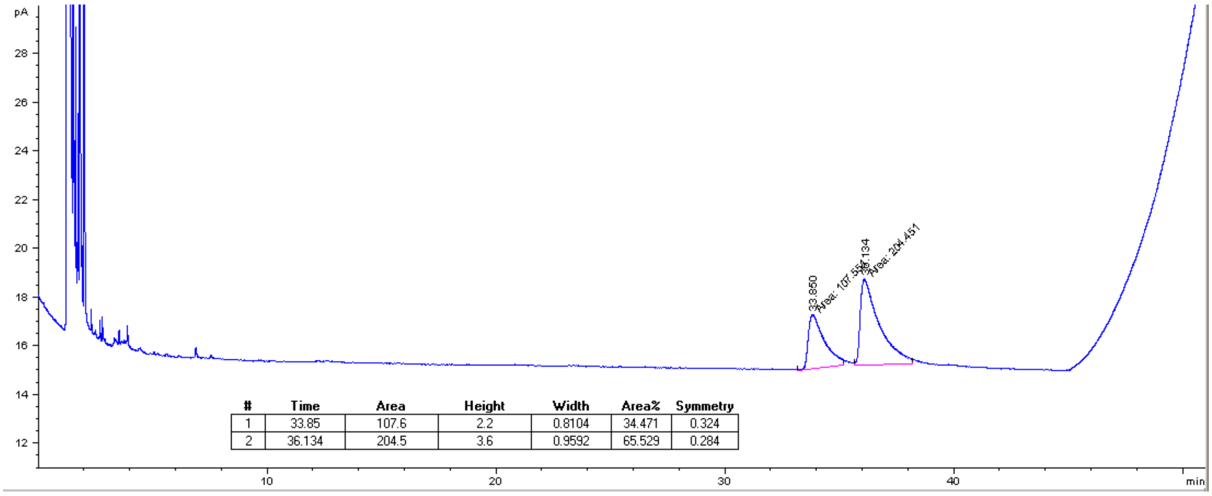

Figure S55. Chromatogram of hydroxylacton **7a** obtained from chlorolactone **3a** by F. culmorum AM22.

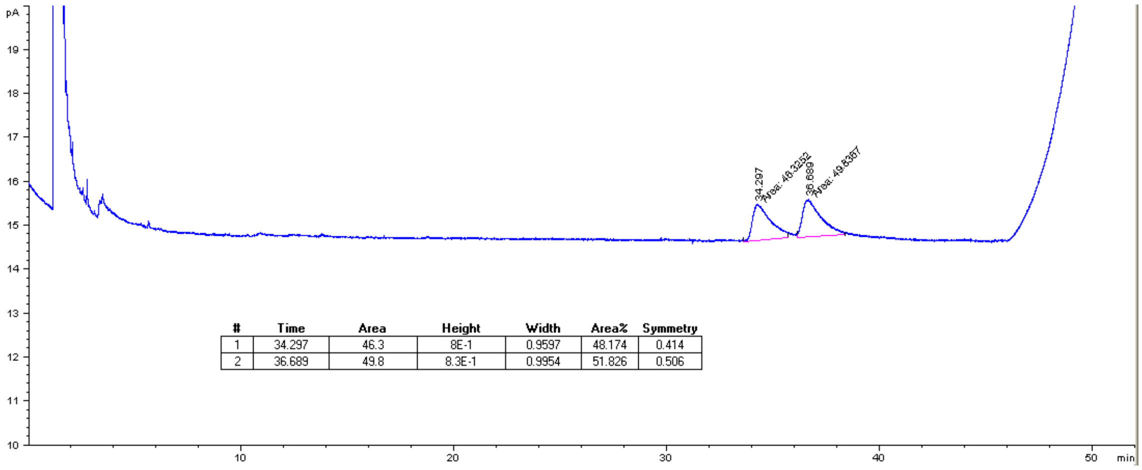

Figure S56. Chromatogram of hydroxylacton **7a** obtained from chlorolactone **3a** by F. culmorum AM203.

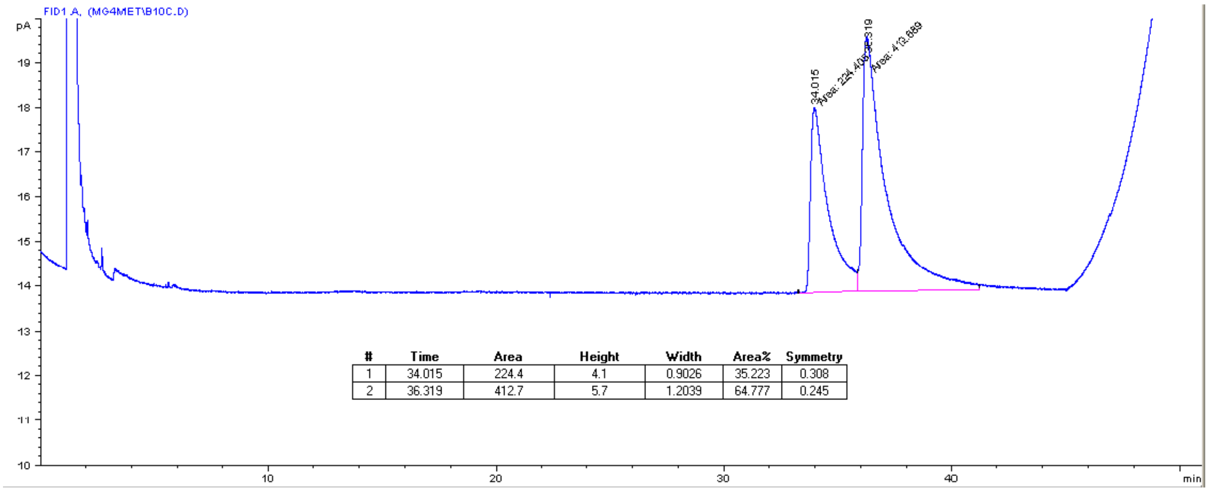

#	Time	Area	Height	Width	Area%	Symmetry
1	34.015	224.4	4.1	0.9026	35.223	0.308
2	36.319	412.7	5.7	1.2039	64.777	0.245

Figure S57. Chromatogram of hydroxylacton **7a** obtained from bromolactone **4a** by F. culmorum AM10.

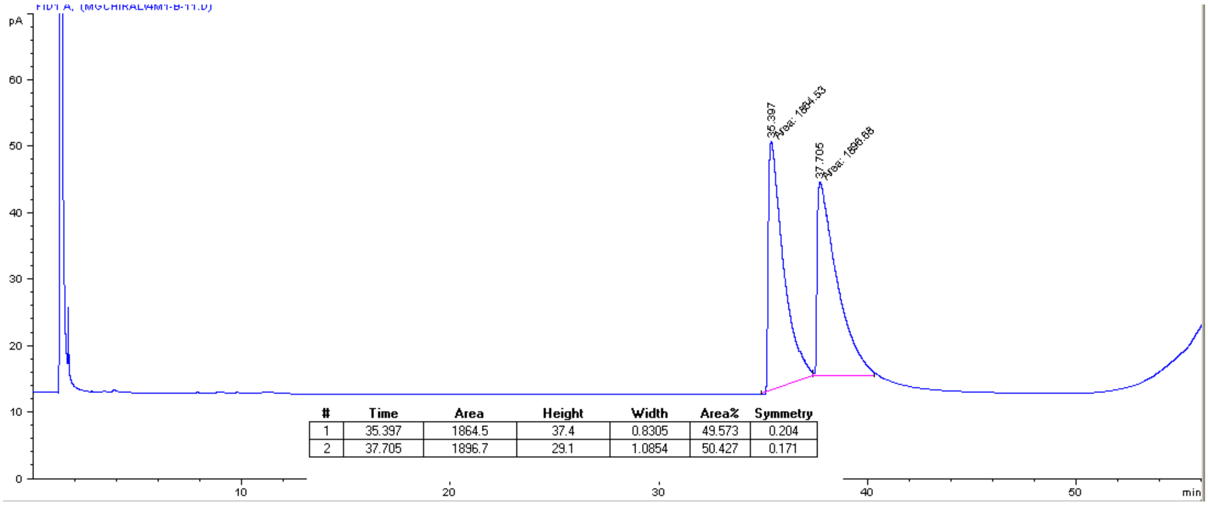

#	Time	Area	Height	Width	Area%	Symmetry
1	35.397	1864.5	37.4	0.8305	49.573	0.204
2	37.705	1896.7	29.1	1.0854	50.427	0.171

Figure S58. Chromatogram of hydroxylacton **7a** obtained from bromolactone **4a** by F. culmorum AM11.

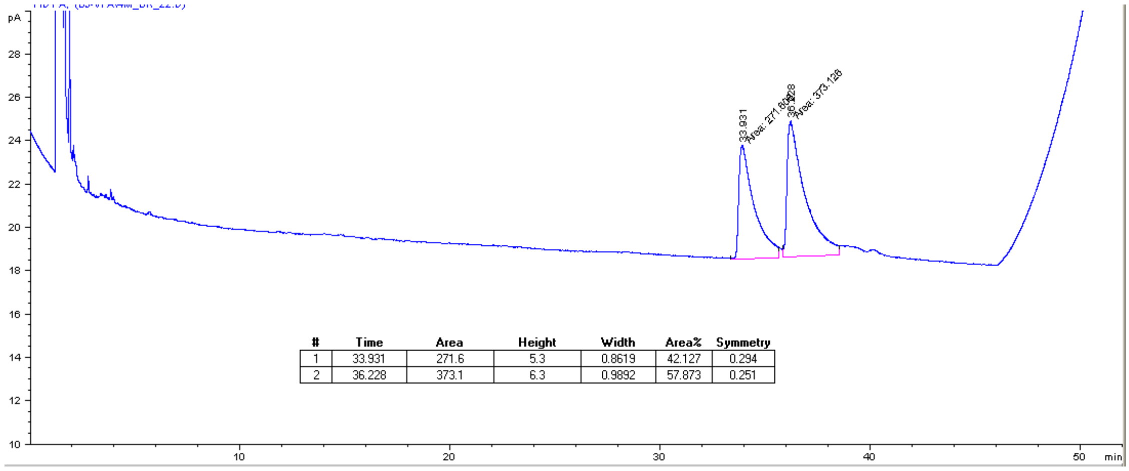

#	Time	Area	Height	Width	Area%	Symmetry
1	33.931	271.6	5.3	0.8619	42.127	0.294
2	36.228	373.1	6.3	0.9892	57.873	0.251

Figure S59. Chromatogram of hydroxylacton **7a** obtained from bromolactone **4a** by F. culmorum AM22.

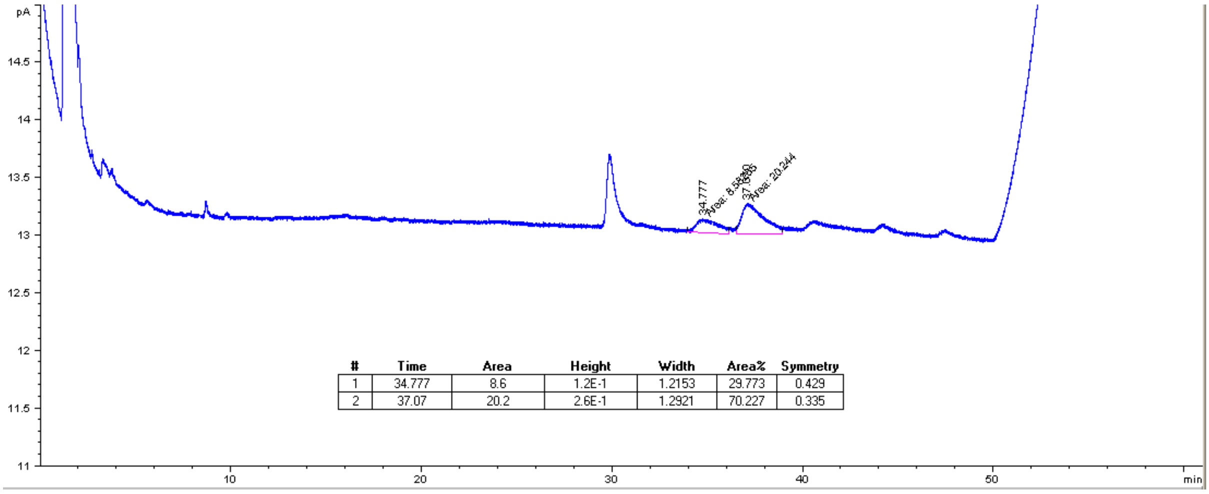

#	Time	Area	Height	Width	Area%	Symmetry
1	34.777	8.6	1.2E-1	1.2153	29.773	0.429
2	37.07	20.2	2.6E-1	1.2921	70.227	0.335

Figure S60. Chromatogram of hydroxylacton **7a** obtained from bromolactone **4a** by F. culmorum AM199.

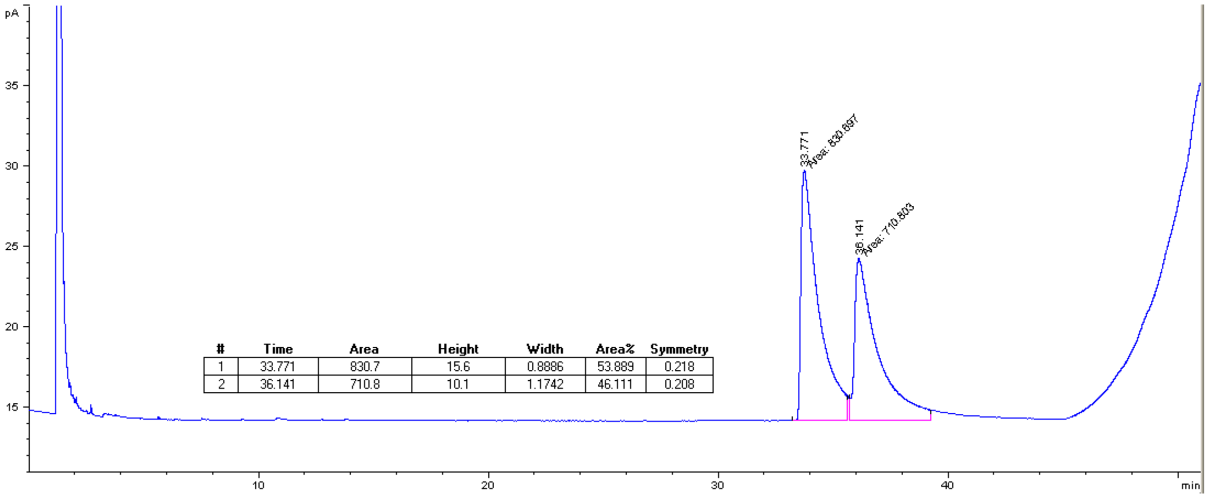

#	Time	Area	Height	Width	Area%	Symmetry
1	33.771	830.7	15.6	0.8886	53.889	0.218
2	36.141	710.8	10.1	1.1742	46.111	0.208

Figure S61. Chromatogram of hydroxylacton **7a** obtained from bromolactone **4a** by F. culmorum AM203.

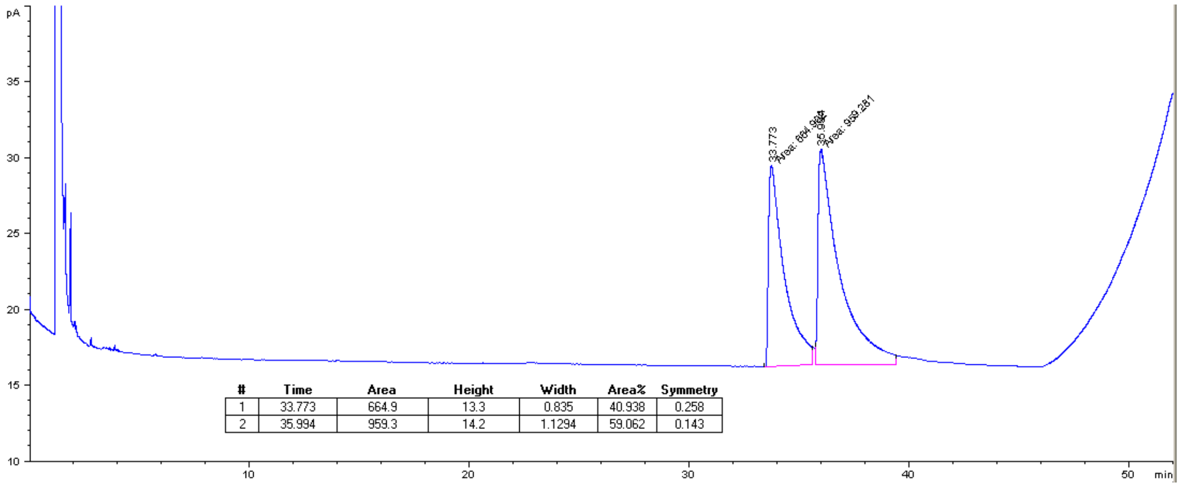

#	Time	Area	Height	Width	Area%	Symmetry
1	33.773	664.9	13.3	0.835	40.938	0.258
2	35.994	959.3	14.2	1.1294	59.062	0.143

Figure S62. Chromatogram of hydroxylacton **7a** obtained from bromolactone **4a** by F. culmorum AM235.

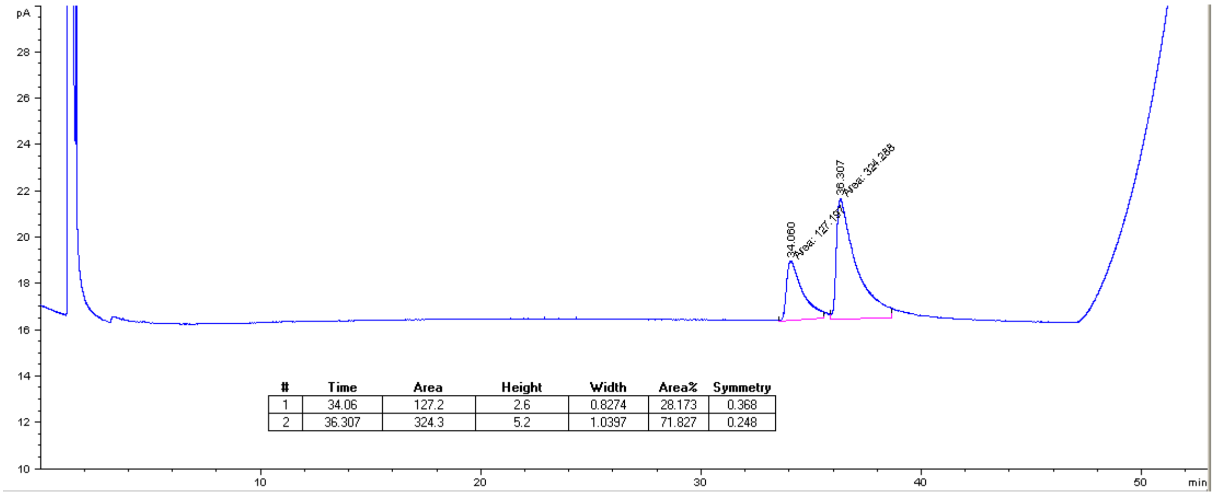

Figure S63. Chromatogram of hydroxylacton **7a** obtained from iodolactone **5a** by F. culmorum AM10.

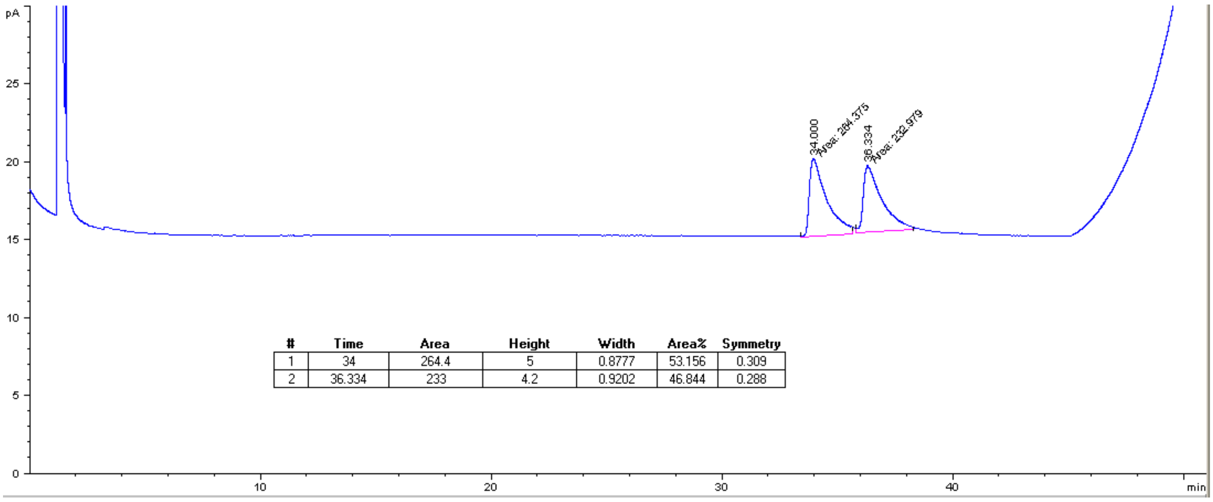

Figure S64. Chromatogram of hydroxylacton **7a** obtained from iodolactone **5a** by F. culmorum AM11.

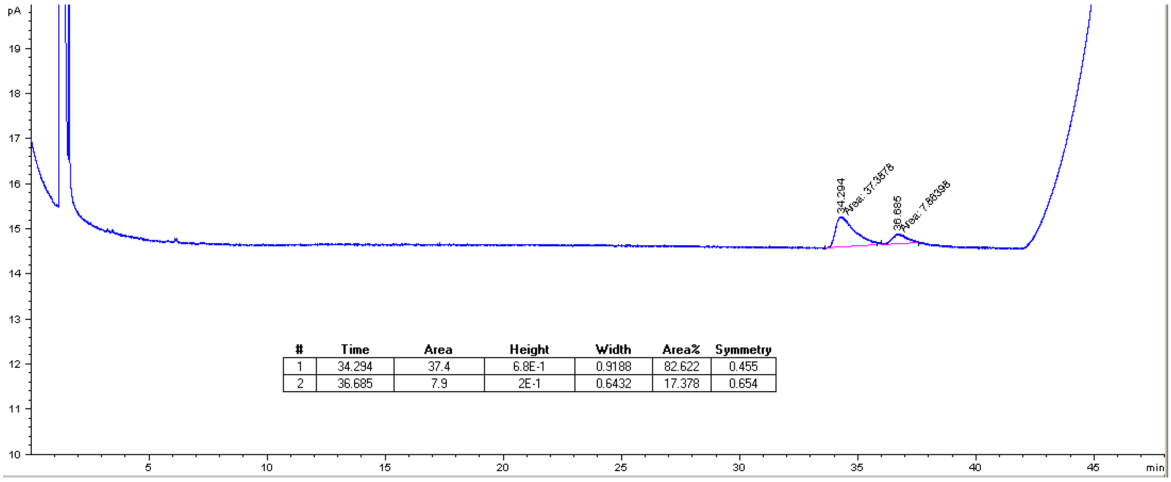

Figure S65. Chromatogram of hydroxylacton **7a** obtained from iodolactone **5a** by F. culmorum AM13.

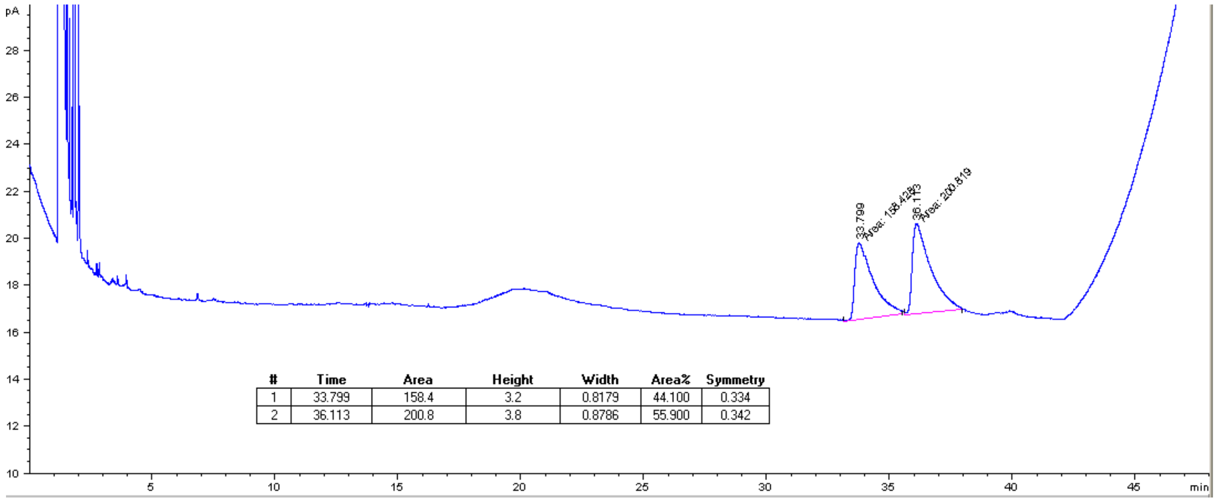

#	Time	Area	Height	Width	Area%	Symmetry
1	33.799	158.4	3.2	0.8179	44.100	0.334
2	36.113	200.8	3.8	0.8786	55.900	0.342

Figure S66. Chromatogram of hydroxylacton **7a** obtained from iodolactone **5a** by F. culmorum AM22.

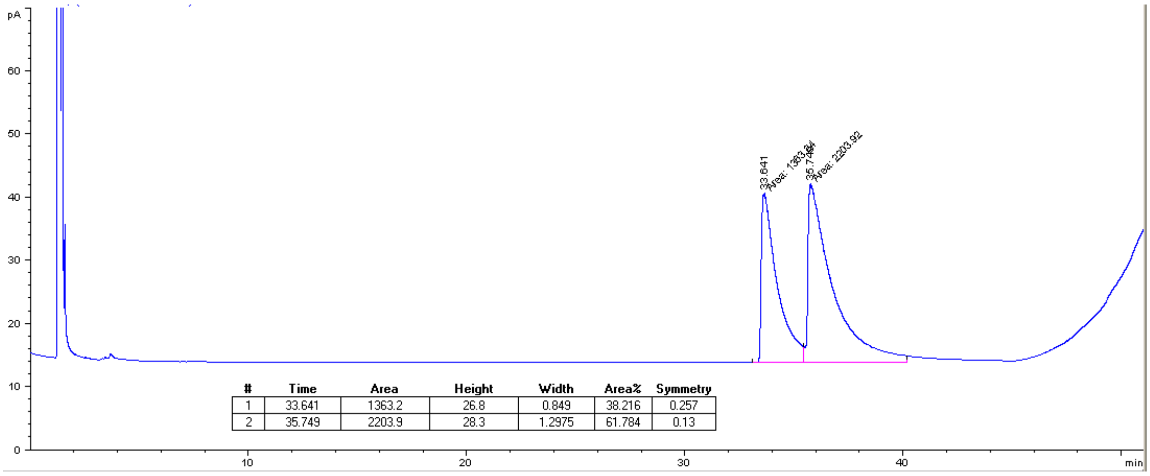

#	Time	Area	Height	Width	Area%	Symmetry
1	33.641	1363.2	26.8	0.849	38.216	0.257
2	35.749	2203.9	28.3	1.2975	61.784	0.13

Figure S67. Chromatogram of hydroxylacton **7a** obtained from iodolactone **5a** by F. culmorum AM105.

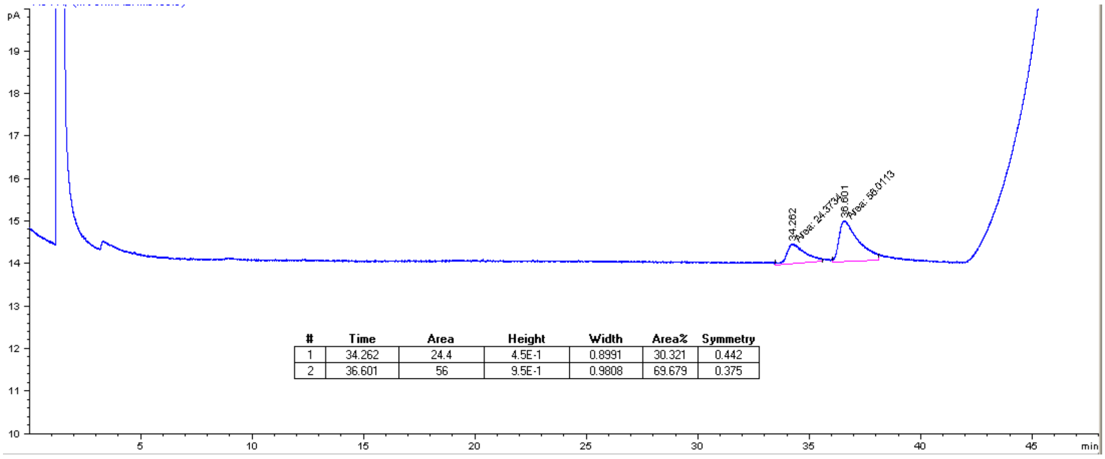

#	Time	Area	Height	Width	Area%	Symmetry
1	34.262	24.4	4.5E-1	0.8991	30.321	0.442
2	36.601	56	9.5E-1	0.9808	69.679	0.375

Figure S68. Chromatogram of hydroxylacton **7a** obtained from iodolactone **5a** by F. culmorum AM199.

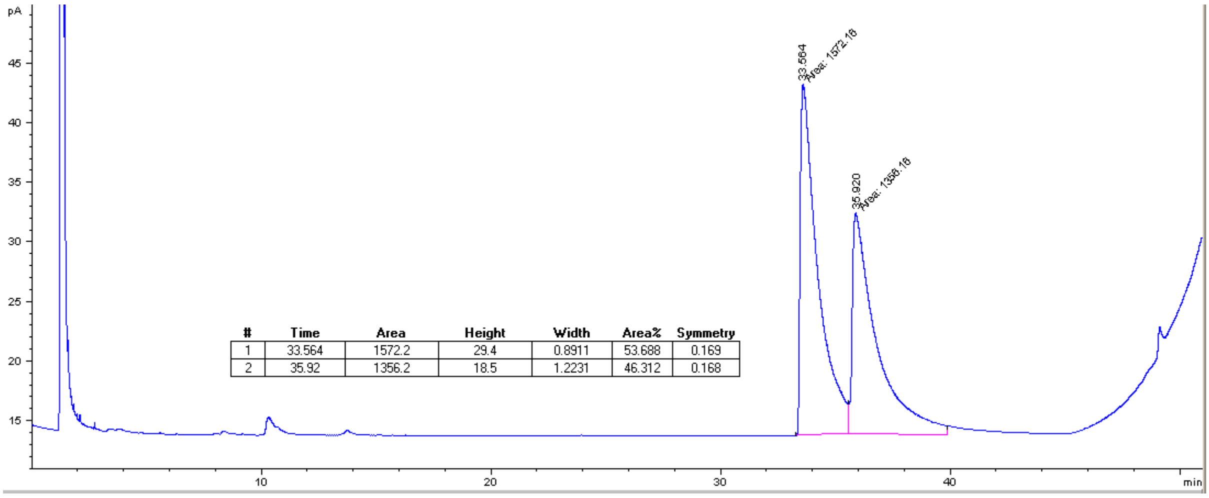

Figure S69. Chromatogram of hydroxylacton **7a** obtained from iodolactone **5a** by F. culmorum AM203.

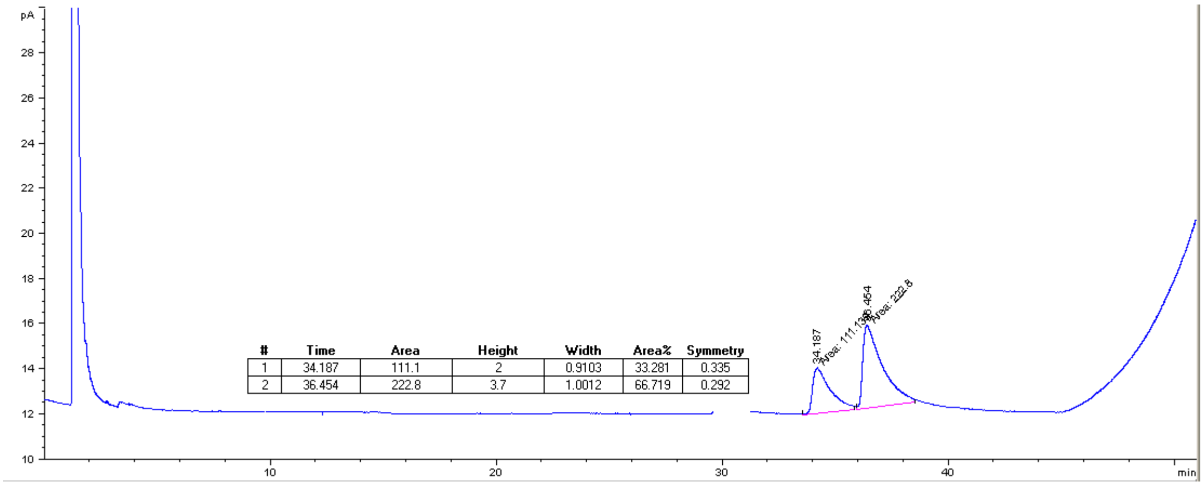

Figure S70. Chromatogram of hydroxylacton **7a** obtained from iodolactone **5a** by F. culmorum AM235.
